# Causal association between sleep traits and the risk of coronary artery disease in patients with diabetes

**DOI:** 10.3389/fcvm.2023.1132281

**Published:** 2023-03-03

**Authors:** Mengyun Tian, Hongchuang Ma, Jiaxi Shen, Teng Hu, Hanbin Cui, Ning Huangfu

**Affiliations:** ^1^ School of Medicine, Ningbo University, Ningbo, China; ^2^ Department of Cardiology, Ningbo First Hospital, Ningbo, China; ^3^ Department of Cardiology, Key Laboratory of Precision Medicine for Atherosclerotic Diseases of Zhejiang Province, Ningbo, China; ^4^ Clinical Medicine Research Centre for Cardiovascular Disease of Ningbo, Ningbo, China

**Keywords:** sleep traits, coronary artery disease, diabetes, causal association, insomnia

## Abstract

**Background and aims:**

The association between sleep traits and coronary artery disease (CAD) in patients with diabetes has been reported in previous observational studies. However, whether these potential relationships are causal remains unclear. We aim to assess the causal relationship between sleep traits and CAD in diabetic.

**Methods:**

Genetic instrumental variables associated with five sleep-related traits (insomnia, sleep duration, ease of getting up, morningness and snoring) were extracted from corresponding genome-wide association studies (GWAS). The associations of genetic variants with CAD were based on 15,666 individuals with diabetes (3,968 CAD cases and 11,696 controls). The primary analysis was derived using the inverse variance weighting method. Further sensitivity analysis was conducted to confirm the robustness and consistency of the main results.

**Results:**

Genetic liability to insomnia was significantly related to the increased risk of CAD in individuals with diabetes [odds ratio (OR): 1.163; 95% CI: 1.072–1.254; *p *= 0.001]. Suggestive evidence was found for the borderline associations between both sleep duration (OR: 0.629; 95% CI: 0.380–1.042, *p *= 0.072) and snoring (OR: 1.010, 95% CI: 1.000–1.020, *p *= 0.050) with CAD risk. However, no consistent evidence was found for the association between ease of getting up and morningness with the risk of CAD in diabetic. Similar results can be verified in most sensitivity analyses.

**Conclusions:**

We provide consistent evidence for the causal effect of insomnia on the increased risk of CAD in individuals with diabetes. The management of sleep health should be emphasized to prevent CAD in diabetic patients.

## Introduction

Cardiovascular disease (CVD) is the leading cause of global mortality and a principle contributor to disability (1). Meanwhile, CVD remains a major cause of death among patients with diabetes (2). The latest diabetes management guidelines published by ESC/ESAD elevated the status of cardiovascular risk in the management of type 2 diabetes mellitus (T2DM) (3). Several clinical studies have been published on cardiovascular risk management in adults with T2DM, involving lifestyle, blood pressure, blood glucose, cholesterol management and sleep in primary and secondary prevention of CVD (4–8).

Sleep disorders are increasingly prevalent modifiable risk factors for CVD (9). Plenty of epidemiological studies suggested that sleep duration was associated with an increased risk of CVD events and higher mortality risk (10–14). A recent prospective study including 18,876 patients with T2DM reported that short and long sleep durations were both independently associated with the increased incidence and mortality of CVD (15). Another study based on 36,058 Korean new-onset T2DM patients indicated that sleep disturbance was significantly associated with an increased risk of CVD mortality (16). Besides, a cross-sectional study with small samples found that the effect of poor sleep on the risk of CVD in patients with T2DM may be mediated by some inflammatory factors (17). Involvement of the endothelial function, autonomic nervous system, regulation of metabolism and inflammation have been proposed as possible mechanistic linked between sleep disorders and CVD (18). Some studies also suggested that sleep disorders contributed to the risk of T2DM by affecting insulin production (19–22). However, the causality between these associations remains unclear, especially in individuals with diabetes.

Mendelian randomization (MR) uses genetic variation as a natural experiment to study the causal relationship between potentially modifiable risk factors and health outcomes (23). As alleles follow random assignment during gamete formation, the estimations would not be affected by confounding factors and reverse causality compared to observational studies (24). Several previous studies have provided genetic evidence for a causal association of insomnia and sleep duration with increased risk of CAD in general population (13, 25). However, the causal association pattern in the diabetics remains unclear. Besides, the causal effect of other sleep characteristics, such as ease of getting up, morningness (being a morning person rather than an evening person) and snoring needs to be further investigated. As the sleep traits are driven by genetic risk, the MR study could provide long-term stable genetic evidence, which is not affected by lifestyle factors.

In the current study, we conducted a comprehensive MR study to evaluate the causal associations between five sleep traits and genetic susceptibility to CAD in individuals with diabetes (Table 1).

**Table 1:** Detailed information on data sources.

Trait	Data source	Sample size (case/controls)	Population
Insomnia	UKB,23andMe	397,959/933,051	European
Sleep duration	UKB	446,118	European
Easy to get up	UKB	385,949	European
Morningness	UKB,23andMe	432,835	European
Snoring	UKB	359,916	European
CAD in patients with diabetes	UKB	3,968/11,698	European

CAD, coronary artery disease; UKB, the UK biobank; 23andMe, 23andMe company; Easy to get up, ease of getting up in the morning.

## Methods

### Study design

**Figure 1:**
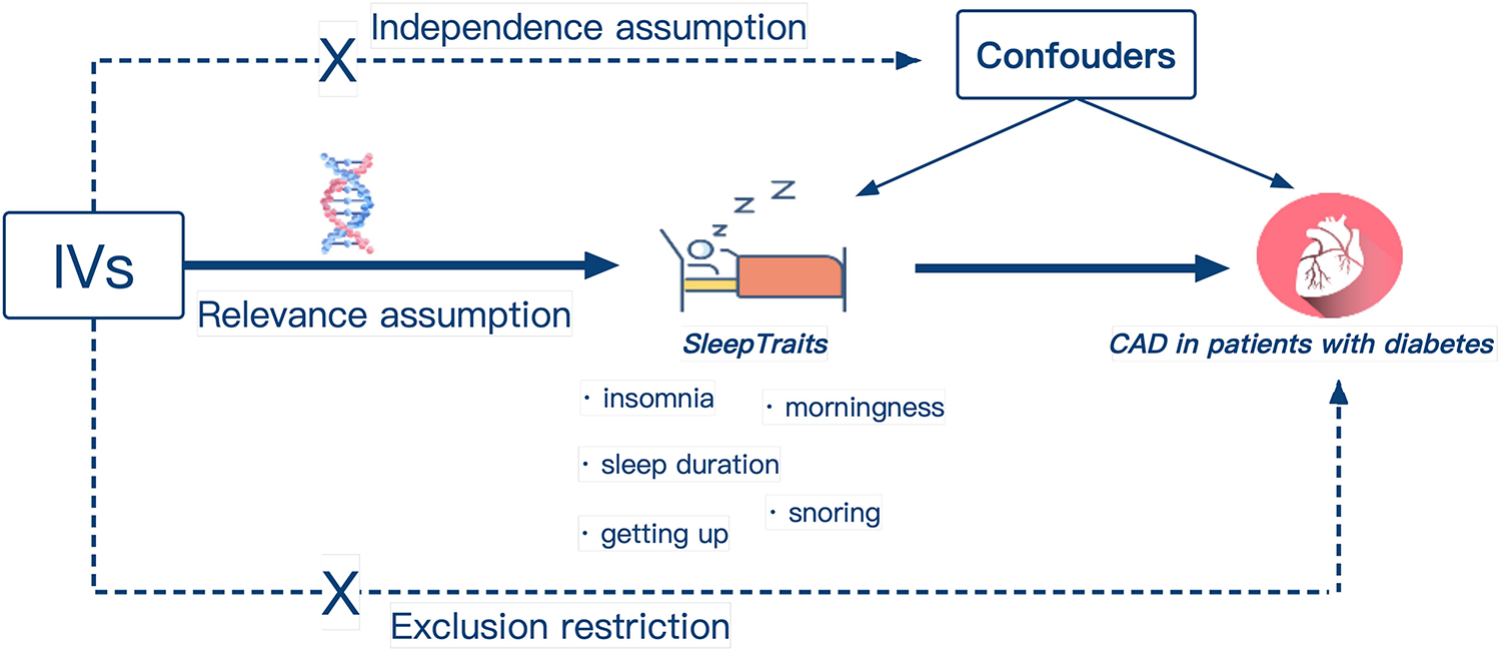
Design of the Mendelian randomization study. Three core assumptions were as follows: Relevance assumption, the genetic IVs must be associated with sleep traits; Independence assumption, IVs should not be associated with confounders; Exclusion restriction, IVs must influence CAD in patients with diabetes only *via* sleep traits. IVs, instrumental variables; Easy to get up, ease of getting up in the morning; CAD, coronary artery disease.

The study design overview was shown in Figure 1. The single nucleotide polymorphisms (SNPs) identified as genetic instrumental variables (IVs) for sleep traits depend on the following three core assumptions: (I) The genetic variation should be strongly related to sleep traits, (II) genetic variation is not influenced by potential confounding factors, as well as (III) genetic variation should only be related to the risk of CAD in diabetic patients through sleep traits (26). Ethical review approval and informed consent were obtained from participants for all included original studies.

### Genetic instrument selection

#### Insomnia

Genetic association with insomnia was obtained from the largest GWAS to date including 1,331,010 individuals of European ancestry (27). Insomnia was measured by a standardized question: “Do you often fall asleep late at night or wake up at midnight?”. There were four answer options: “never/rarely”, “sometimes”, “usually”, or “prefer not to answer”. Among them, the participants who answered “usually” were defined as suffering from insomnia, while the participants who answered “never or rarely” and “sometimes” were included in the control group.

#### Sleep duration

The sleep duration GWAS analysis identified 78 independent SNPs associated with sleep duration (*p *< 5 × 10^−8^), involving 446,118 individuals of European ancestry (28). The sleep duration was determined in the form of self-report. Subjects were asked “How long do you sleep every 24 h, including naps”, and the answers increased in hours. Subjects who responded to extreme values (less than 3 h or more than 18 h) or who reported using any sleep-regulating medication were excluded. Sleep duration was examined continuously.

### Ease of getting up in the morning

The ease of getting up was investigated in a study including 1,331,010 participants by asking: “On an average day, how easy do you find getting up in the morning?”. The possible responses included “not at all easy”, “not very easy”, “fairly easy” and “very easy”. The ease of getting up was divided into four categories and examined as a continuous scale. The corresponding GWAS extracted 70 independent pilot SNPs located in 62 different genomic loci (27).

#### Morningness

Morningness was evaluated by asking: “Do you consider yourself to be?”. There were five possible responses “Definitely a ‘morning’ person”, “More a ‘morning’ than ‘evening’ person”, “More an ‘evening’ than a ‘morning’ person”, “Definitely an ‘evening’ person”, and “Do not know”. The corresponding GWAS identified 274 independent pilot SNPs located in 207 different genomic loci (27).

#### Snoring

Snoring was evaluated based on asking: “Does your partner or a close relative or friend complain about your snoring?”. People would reply with “yes” or “no”. A total of 3,59,916 subjects were included in the genome-wide analysis of snoring. The corresponding GWAS analysis revealed 3,416 GWS SNPs (*p *< 5 × 10^−8^), resulting in the identification of 42 SNPs associated with snoring, which were located in 36 different genomic loci (27).

#### CAD in patients with diabetes

The summary statistical data of CAD in diabetic were obtained from the latest GWAS (29). That study was conducted on the basis of the UK Biobank cohorts in 2018, including 15,666 European individuals with diabetes (3,968 CAD cases and 11,696 non-cases). The diabetes and CAD were defined based on linked data from hospital admissions and death registries, and verbal health interview (see Supplementary Methods). The average age at visit was 62.7 ± 5.6 and 60.2 ± 7.0 for CAD group and non-CAD group, respectively. 74.0% of CAD group and 60.2% of non-CAD group were male. In the CAD group, 268 (6.8%) individuals were with type I diabetes, while in the non-CAD group 945 (8.1%) individuals were with type I diabetes. Linkage disequilibrium (LD) testing was performed based on 1,000 genomes LD reference panel of Europeans only (30).

### Statistical analysis

The inverse variance weighting (IVW) method was applied to evaluate the influence of genetically predicted sleep traits on the risk of CAD in diabetic populations in the main analysis (31). The Wald estimator was used to generate a causal estimate for each SNP and the standard error was obtained using the Delta method. The overall effect value was then obtained by combining these estimates (32).

Sensitivity analysis was performed using the maximum likelihood method, the weighted median method, the MR-Egger method, and the Mendelian Randomization Pleiotropy Residual Sum and Outlier (MR-PRESSO) method to evaluate the robustness of the results. The weighted median method allows accurate calculation of causal association effects, even when less than 50% of the genetic variation violates the core assumptions (33). The MR-PRESSO can exclude specific outliers to obtain estimates closer to the true value and detect horizontal gene pleiotropy (34). The MR-Egger intercept test was used to evaluate possible horizontal pleiotropic effects and to visually examine potential multidirectional effects by generating funnel plots (35). The causal association between exposure and health outcomes was described by using scatter plots. Subsequently, the leave-one-out sensitivity analyses were performed to assess whether the casual relationship was dramatically driven by any single SNP. All the statistical analyses were conducted using the TwoSampleMR and MR-PRESSO packages in the R software (Version 4.3.1).

## Results

### Insomnia

**Figure 2:**
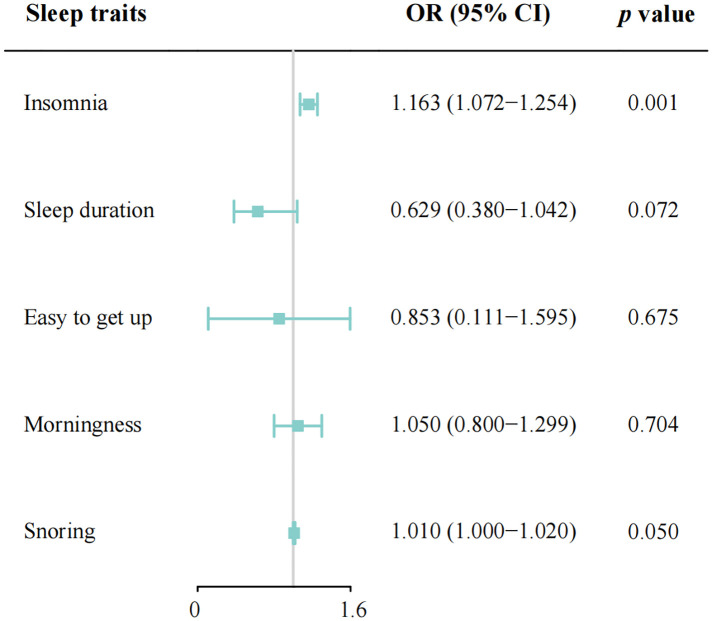
Mendelian randomization estimates of genetically predicted sleep disorders on coronary artery disease in patients with diabetes. Easy to get up, ease of getting up in the morning; OR, odds ratio; CI, confidence interval; IVW, inverse-variance weighted.

**Figure 3:**
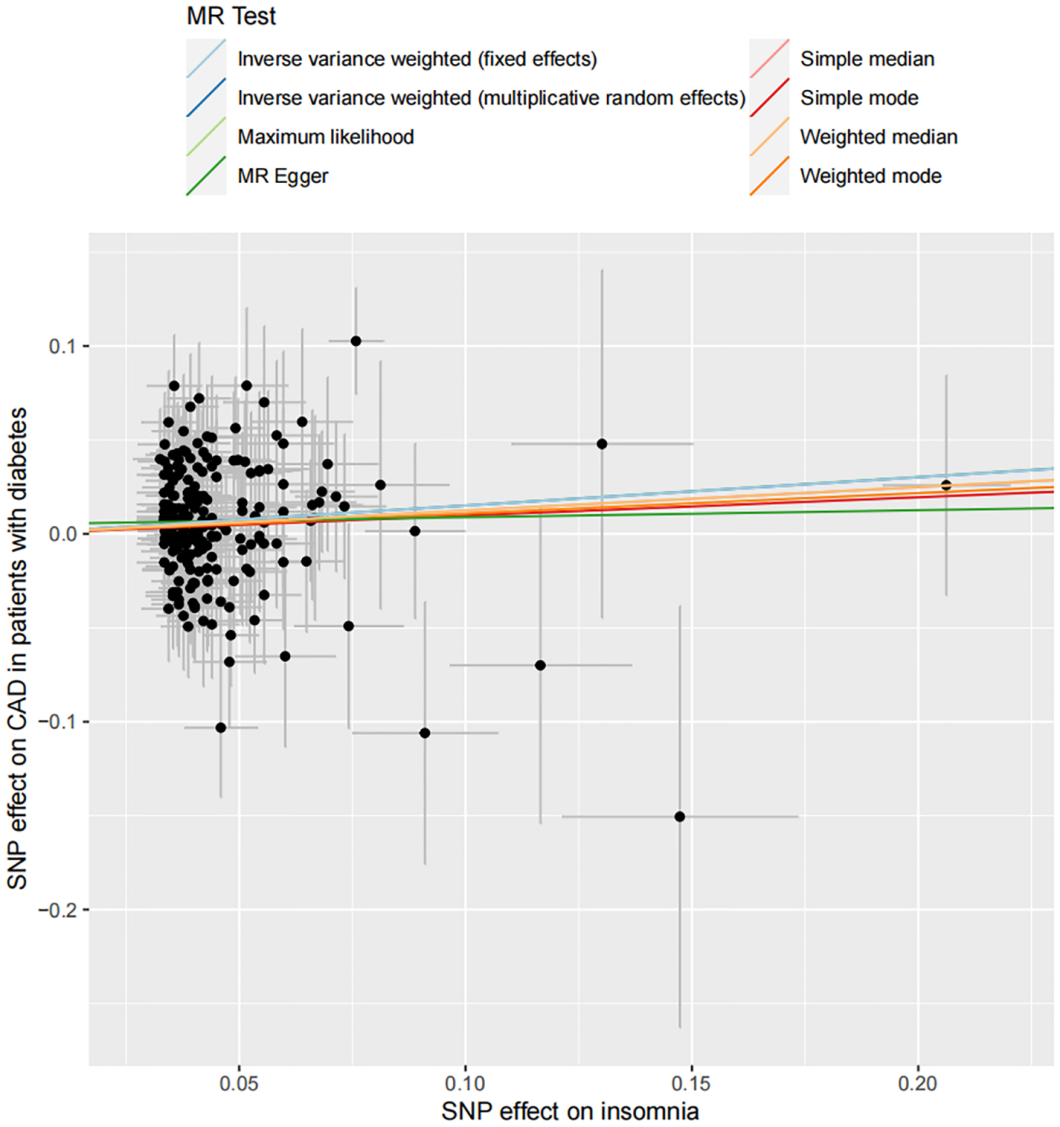
Scatter plot of the association between insomnia and the risk of CAD in patients with diabetes. Each dot indicates a SNP; each line indicates the estimate of association between insomnia and the risk of CAD in patients with diabetes using corresponding methods.

In total, 208 SNPs were identified and used as the genetic IVs for insomnia. The genetic liability to insomnia in diabetic patients showed a significant association with an increased risk of CAD in the IVW analysis (OR: 1.163; 95% CI: 1.072–1.254, *p* = 0.001; Figure 2). The main results remained consistent in the sensitivity analysis using the weighted median and maximum likelihood method (Supplementary Table S7). MR-Egger regression analysis showed no overall pleiotropy or heterogeneity between insomnia and CAD in diabetic patients (*p *> 0.05) (Table 2). In addition, scatter plots also showed that there was no directional polymorphism on CAD in diabetic patients (Figure 3). The leave-one-out sensitivity analysis indicated that the causal association was not greatly driven by any single SNP (Supplementary Figure S1).

**Table 2:** MR-Egger regression intercept tests.

Exposure	Outcome	Intercept (95% CI)	*p*-value
Insomnia	CAD in diabetic	0.005 (−0.011, −0.021)	0.539
Sleep duration	CAD in diabetic	0.004 (−0.024, −0.031)	0.790
Easy to get up	CAD in diabetic	0.016 (−0.017, −0.048)	0.346
Morningness	CAD in diabetic	0.008 (−0.006, −0.021)	0.268
Snoring	CAD in diabetic	−0.154 (−0.449, −0.140)	0.313

MR, mendelian randomization; CI, confidence interval; CAD, coronary artery disease; Easy to get up, ease of getting up in the morning.

#### Other sleep traits

With the IVW analysis, the current study found suggestive evidence for the borderline associations between both sleep duration (OR: 0.629; 95% CI: 0.380–1.042, *p *= 0.072; Figure 2) and snoring (OR: 1.010, 95% CI: 1.000–1.020, *p *= 0.050) with CAD risk in individuals with diabetes, which needed to be further investigated. Horizontal pleiotropy was not detected using MR-Egger intercept testing (Supplementary Table S7). However, no consistent evidence was found for the causal associations between ease of getting up (OR: 0.853; 95% CI: 0.111–1.595; *p *= 0.675) and morningness (OR: 1.050; 95% CI: 0.800–1.299; *p *= 0.704) with the risk of CAD in diabetic patients. Besides, in the MR-Egger analysis, no pleiotropy was detected between ease of getting up and morningness with CAD risk (Supplementary Table S7). The association patterns kept consistent and robust in the other three statistical models (Supplementary Table S7).

## Discussion

In this study, we investigated the causal effects of five sleep traits including insomnia, sleep duration, ease of getting up, morningness and snoring on CAD in patients with diabetes. Genetic liability to insomnia was closely related to the increased CAD risk in individuals with diabetes. Besides, suggestive evidence was found for the borderline associations between both sleep duration and snoring with the risk of CAD, which needed to be further investigated. However, no valid evidence for the causal effects of morningness and ease of getting up was found.

In regards to the directionality of the observed effects, we found a significant association between insomnia and increased CAD risk in patients with diabetes. Besides, the results suggested the borderline associations between sleep duration with reduced CAD risk and snoring with increased CAD risk, which were in line with previous observational studies. The easy to get up showed a trend of protective factors, however no consistent evidence was found. There were large differences in variation between the ORs of different sleep traits, partly due to the difference between binary variable (e.g., insomnia) and continuous variable (e.g., sleep duration). Besides, the causal effects of morningness and snoring on CAD risk may be relatively limited, compared to insomnia.

In the recent years, increasing observational evidence supported that insomnia was an important risk factor for the progression of CVD. Previous studies reported that people with significant insomnia symptoms have a 41%–55% increased risk of myocardial infarction and coronary heart disease, as well as a higher risk of cardiovascular and cerebrovascular related death. Suzanne M.Bertisch et al. found that insomnia or poor sleep quality combined with frequent short sleep increased the incidence of CVD events by 29% compared with controls in a propensity matching model (36). A large population cohort study showed that short or long sleep duration, insomnia and snoring were all related to an increased risk of CVD in a multivariate model (14). In a prospective cohort study involving 4,07,500 individuals, the incidence of CVD was significantly associated with a 7%, 26%, and 20% increased risk of snoring, insomnia, and narcolepsy, respectively (37).

Shorter sleep duration was independently associated with increased risk of subclinical multiple atherosclerosis (38). Among patients younger than 40 years of age, insomniacs had a higher risk of T2DM than controls (HR: 1.31; 95% CI: 1.14–1.55) (39). Insomnia symptoms may lead to an increase in glycated hemoglobin levels, suggesting a causal link between insomnia and T2DM (40). In addition, short and long sleep duration would increase the risk of T2DM (41). Another prospective cohort study also indicated that insomnia was associated with a higher risk of T2DM (42). And a meta-analysis of 13 prospective trials demonstrated that insomnia would increase the risk of CVD (43).

The pathophysiological basis and mechanisms underlying the association of insomnia with CVD have not been fully clarified. Possible mechanisms may include dysregulation of the hypothalamic-pituitary axis (HPA) (44), dysregulation of the autonomic nervous system (45), systemic inflammatory activation (46), and acceleration of atherosclerosis (47). First, sleep was linked to haematopoiesis and atherosclerosis in mice, and sleep fragmentation would lead to more Ly-6Chigh monocytes, larger atherosclerotic lesions and less hypocretin, which controls myelopoiesis. Second, insomnia may affect the endothelial function of coronary arteries through the autonomic nervous system, thereby accelerating coronary atherosclerosis (48). Third, sleep disturbance could induce increased NF-κB, proinflammatory cytokine production, and systemic inflammation through activation of β-adrenergic signaling pathways (49). Forth, sleep disorders can also lead to the occurrence of CAD by affecting the endocrine mechanism, inhibiting the metabolism of blood lipids and the regulation of blood glucose (50).

The main strength was the study design using multiple SNPs as genetic IVs for sleep characteristics, which minimized confounding and reverse causality. In the current study, sleep traits were driven by genetic risk and not modifiable, thus providing stable genetic evidence, which was not affected by lifestyle factors. In addition, as appropriate genetic IVs were screened by a large amount of genetic associations, the summary of data guaranteed the estimation of causal effect on the basis of the large sample. Besides, we evaluated the causal effect of five sleep traits on CAD in individuals with diabetes using a comprehensive analysis, which could greatly expand the scope of our discovery.

Several limitations of the current study should be pointed out. First, the selection of genetic instruments was based on the GWAS without hypothesis, which may lack a comprehensive understanding of the potential association mechanism between genetic variation and diseases. Second, it was impossible to completely exclude the potential effects of pleiotropy, thus the results may be affected. Third, our findings could not be generalized to other populations, as only European participants were included. Therefore, further studies were needed to investigate the causal association pattern in other populations. Forth, all the sleep traits were obtained through subjective description of patients, so it was difficult to avoid misclassification. Besides, due to lack of individual-level genetic data, stratification analysis was not available in the current study to assess the gender and/or age differences in the association between sleep and CAD risk. Likewise, we failed to perform the analysis on if the insomnia treatments influence the association with CAD, which needed to be further investigated.

## Conclusion

In conclusion, we provided consistent evidence for the causal effect of insomnia on the increased CAD risk in diabetic patients. The borderline associations between both sleep duration and snoring with the risk of CAD needed to be further investigated. Increased attention should be paid to sleep health and better prevention of sleep disorder, which may reduce the risk of CAD in diabetic patients.

## Data Availability

The datasets presented in this study can be found in online repositories. The names of the repository/repositories and accession number(s) can be found in the article/**Supplementary Material**.
