# Supplementary material for: Causal association between sleep traits and the risk of coronary artery disease in patients with diabetes
Source: Front Cardiovasc Med. 2023 Mar 3;10:1132281. doi: 10.3389/fcvm.2023.1132281 (PMC10020648; doi:10.3389/fcvm.2023.1132281)
Supplement: Supplementary file 1 [file Datasheet1.pdf]

Supplementary method

The diabetes and CAD were defined based on UK Biobank's baseline assessment verbal health interview, combined with linked data from hospital admissions and death registries. The average age at visit was 62.7±5.6 and 60.2±7.0 for CAD group and non-CAD group, respectively. 74.0% of CAD group and 60.2% of non-CAD group were male. In the CAD group, 268 (6.8%) individuals were with type I diabetes, while in the non-CAD group 945 (8.1%) individuals were with type I diabetes. The rest were with type II diabetes. Specifically, CAD was defined as having a recorded death or hospitalization with primary or secondary diagnosis recorded with the ICD version 10 codes I20, angina pectoris; I21, acute myocardial infarction; 122 subsequent ST elevation (STEMI) and non-ST elevation (NSTEMI) myocardial infarction; I23, certain current complications following ST elevation (STEMI) and non-ST elevation (NSTEMI) myocardial infarction; I24, other acute ischaemic heart diseases; or I25, chronic ischaemic heart disease and ICD-9 codes: 410, acute myocardial infarction; 411, other acute and subacute forms of ischaemic heart disease; 412, old myocardial infarction or 413 angina pectoris. Further, individuals were classified as CAD if they reported angina pectoris or myocardial infarction at the verbal interview. A trained nurse and a doctor examined and matched it to entries in the coding tree. Individuals not fulfilling the above criteria were defined as not having CAD.

Table S1. Genome-wide significant ($p<5\times10^{-8}$) single nucleotide polymorphisms that were used as instruments for insomnia.

SNP	Chr	Pos	EA	OA	EAF	Beta	Se	*P*-value
rs699844	1	74878253	A	G	0.92	0.060	0.011	4.1E-08
rs6702604	1	107190062	A	G	0.58	-0.037	0.006	1.3E-09
rs623025	1	201765094	T	C	0.26	-0.038	0.007	3.2E-08
rs5877	1	173878862	T	C	0.67	0.036	0.006	1.2E-08
rs2089358	1	37194103	T	C	0.70	-0.041	0.007	2.7E-10
rs1937447	1	66358242	C	G	0.76	-0.039	0.007	2.1E-08
rs1620977	1	72729142	A	G	0.27	0.052	0.007	2.3E-14
rs1289939	1	117944435	T	C	0.23	-0.041	0.007	6.0E-09
rs12030482	1	96961268	A	T	0.22	0.041	0.007	8.2E-09
rs11803128	1	190060095	A	G	0.65	-0.041	0.006	6.9E-11
rs11588755	1	57819204	A	G	0.52	-0.035	0.006	5.1E-09
rs11119409	1	210293333	T	C	0.59	-0.035	0.006	1.2E-08
rs10800992	1	190900576	T	C	0.44	0.042	0.006	3.8E-12
rs823247	2	2850540	T	C	0.48	-0.037	0.006	5.2E-10
rs7599697	2	239231477	T	C	0.36	-0.037	0.006	5.0E-09
rs7571486	2	176473295	A	G	0.25	-0.039	0.007	1.4E-08
rs75452188	2	67134426	A	G	0.88	0.052	0.009	1.6E-08
rs72820274	2	104412924	A	G	0.42	0.034	0.006	1.3E-08
rs6756610	2	147480394	C	G	0.63	0.037	0.006	1.1E-09
rs6734957	2	42813247	T	G	0.24	-0.042	0.007	1.8E-09
rs6545798	2	60521311	A	T	0.41	-0.041	0.006	1.2E-11
rs62213452	2	210380152	T	G	0.28	0.037	0.007	2.4E-08
rs62158170	2	114082175	A	G	0.79	0.066	0.007	1.2E-19
rs56097173	2	44262449	T	C	0.68	0.040	0.006	2.7E-10
rs55772859	2	208042581	A	C	0.31	0.042	0.006	4.8E-11
rs4664299	2	160570033	T	C	0.23	-0.041	0.007	5.0E-09
rs34967082	2	215382654	A	G	0.41	0.035	0.006	4.3E-09
rs1861412	2	58893065	A	G	0.43	0.038	0.006	1.7E-10
rs1530938	2	236900633	A	G	0.44	0.036	0.006	8.8E-10
rs1519102	2	66677816	C	G	0.69	-0.037	0.006	1.9E-08
rs13010288	2	51824512	T	G	0.13	-0.060	0.009	9.3E-12
rs12991815	2	68071990	C	G	0.42	0.040	0.006	3.0E-11
rs11679943	2	77724624	A	G	0.35	0.037	0.006	3.2E-09
rs116466468	2	159137557	T	C	0.76	0.044	0.007	2.1E-10
rs113851554	2	66750564	T	G	0.05	0.206	0.014	1.6E-51
rs10928256	2	146458738	T	C	0.42	0.034	0.006	1.6E-08
rs7625896	3	44062561	A	G	0.65	0.036	0.006	5.3E-09

rs7615602	3	18718055	C	G	0.27	-0.040	0.007	2.6E-09
rs694786	3	173112907	T	C	0.46	-0.044	0.006	2.0E-13
rs6808140	3	10581380	T	C	0.51	0.039	0.006	5.4E-11
rs62264767	3	117642005	A	C	0.85	0.065	0.008	1.6E-14
rs492858	3	155432229	T	C	0.08	-0.066	0.011	3.5E-09
rs4858708	3	25154112	A	T	0.53	-0.034	0.006	1.2E-08
rs4260410	3	178469932	T	C	0.33	0.034	0.006	4.9E-08
rs3774751	3	50209053	T	G	0.46	-0.041	0.006	7.3E-12
rs35110063	3	43066558	A	G	0.43	0.039	0.006	8.8E-11
rs2364921	3	158522463	T	C	0.47	-0.034	0.006	2.1E-08
rs2216427	3	180785697	C	G	0.65	0.035	0.006	1.6E-08
rs17025198	3	88001713	A	G	0.20	0.041	0.007	2.2E-08
rs1580173	3	107955515	A	G	0.56	0.033	0.006	2.3E-08
rs1567084	3	71435955	A	G	0.50	0.033	0.006	2.1E-08
rs10865954	3	49211989	T	C	0.33	0.042	0.006	1.9E-11
rs72657797	4	90820809	T	C	0.18	-0.056	0.008	1.5E-12
rs62301574	4	22050165	C	G	0.80	-0.042	0.007	1.4E-08
rs4699157	4	106055212	T	C	0.96	-0.081	0.015	4.0E-08
rs17005118	4	82288564	A	G	0.26	0.042	0.007	6.1E-10
rs16990210	4	34720226	T	C	0.85	-0.046	0.008	2.0E-08
rs13138995	4	148987430	A	G	0.39	0.034	0.006	2.0E-08
rs13135092	4	103198082	A	G	0.92	-0.089	0.011	2.5E-16
rs11722569	4	112822731	T	C	0.66	0.034	0.006	2.9E-08
rs8180457	5	107209814	T	C	0.16	-0.056	0.008	1.1E-11
rs701394	5	80296487	A	G	0.64	-0.036	0.006	6.8E-09
rs6888135	5	141254063	A	C	0.50	0.038	0.006	1.2E-10
rs6601080	5	179511043	A	G	0.68	0.035	0.006	2.2E-08
rs62383308	5	165460085	A	G	0.08	-0.060	0.011	4.0E-08
rs55972276	5	135653737	A	C	0.14	0.073	0.009	4.2E-17
rs4502882	5	153093998	T	C	0.66	-0.039	0.006	8.0E-10
rs35539975	5	91607148	A	G	0.78	0.042	0.007	4.5E-09
rs2431108	5	103947968	T	C	0.67	-0.053	0.006	7.8E-17
rs17367725	5	107112116	T	C	0.35	-0.036	0.006	9.3E-09
rs17223714	5	50492629	A	G	0.79	0.046	0.007	2.4E-10
rs17083297	5	92995477	A	C	0.18	-0.044	0.008	1.6E-08
rs16903122	5	87693561	T	C	0.25	0.055	0.007	9.0E-16
rs152555	5	106849674	A	G	0.85	-0.052	0.008	4.8E-10
rs12520974	5	61514611	T	C	0.48	-0.036	0.006	1.7E-09
rs12187443	5	102660400	T	C	0.67	0.040	0.006	1.6E-10
rs9394502	6	38452503	T	C	0.33	-0.054	0.006	7.8E-18

rs9373590	6	101212001	A	T	0.51	0.040	0.006	2.2E-11
rs728017	6	124292594	A	G	0.39	-0.035	0.006	9.5E-09
rs6457796	6	34828553	T	C	0.73	-0.039	0.007	1.1E-08
rs62429521	6	140324582	A	C	0.15	0.051	0.008	1.8E-09
rs4709655	6	163280204	T	C	0.12	-0.054	0.009	3.1E-09
rs314281	6	105400605	T	C	0.45	-0.043	0.006	6.0E-13
rs3131638	6	31475127	A	G	0.23	-0.044	0.007	7.9E-10
rs2388840	6	99598756	A	G	0.58	-0.037	0.006	1.4E-09
rs138678612	6	30932223	A	G	0.98	-0.117	0.020	1.4E-08
rs11756035	6	18843810	C	G	0.13	0.051	0.009	1.3E-08
rs117152417	6	166411281	A	G	0.01	-0.147	0.026	2.8E-08
rs1147852	6	147980909	A	G	0.31	0.039	0.006	9.9E-10
rs10947987	6	41754370	T	C	0.44	-0.033	0.006	4.1E-08
rs10947690	6	37631768	A	G	0.74	-0.047	0.007	4.0E-12
rs10947428	6	33647058	T	C	0.79	-0.068	0.007	9.1E-21
rs10944696	6	94498850	A	G	0.30	-0.038	0.007	8.0E-09
rs8180817	7	114047542	C	G	0.43	-0.049	0.006	1.8E-16
rs75932578	7	106844694	T	C	0.22	-0.040	0.007	4.1E-08
rs73671843	7	3520024	A	G	0.13	-0.056	0.009	5.5E-10
rs6978112	7	1966841	T	C	0.41	0.034	0.006	2.1E-08
rs6967168	7	132672192	T	G	0.75	-0.044	0.007	1.4E-10
rs670501	7	108625185	T	C	0.21	0.053	0.007	7.4E-13
rs6465151	7	88310899	T	C	0.11	0.056	0.009	1.9E-09
rs521484	7	49894349	A	G	0.77	-0.040	0.007	1.5E-08
rs2598293	7	133989882	T	C	0.48	0.035	0.006	2.5E-09
rs2030672	7	21687925	C	G	0.56	0.034	0.006	1.1E-08
rs190073	7	10985188	A	G	0.41	-0.034	0.006	2.9E-08
rs17520265	7	119674508	A	G	0.03	-0.091	0.016	2.9E-08
rs1731951	7	137075847	A	T	0.44	-0.035	0.006	1.4E-08
rs12666306	7	115082406	A	G	0.50	0.042	0.006	2.2E-12
rs874168	8	30849450	T	C	0.53	0.034	0.006	7.9E-09
rs871994	8	35190619	A	C	0.44	0.035	0.006	5.5E-09
rs671985	8	60914783	A	G	0.45	-0.038	0.006	2.8E-10
rs4588900	8	73890425	A	G	0.52	0.033	0.006	1.6E-08
rs28611339	8	10170037	T	G	0.13	0.058	0.009	8.5E-11
rs28552587	8	103356226	A	G	0.56	0.033	0.006	3.3E-08
rs2737240	8	116657235	A	G	0.71	0.036	0.007	3.4E-08
rs17643634	8	91650818	T	C	0.17	-0.060	0.008	1.3E-13
rs10955647	8	114154187	T	G	0.53	0.033	0.006	1.8E-08
rs77641763	9	140265782	T	C	0.12	0.071	0.009	6.5E-15

rs72773790	9	139109080	T	C	0.67	0.037	0.006	3.7E-09
rs7044885	9	81739348	C	G	0.44	-0.041	0.006	5.7E-12
rs7040224	9	134886837	A	G	0.32	0.037	0.006	4.2E-09
rs6597649	9	133786652	T	C	0.40	0.033	0.006	3.0E-08
rs4090240	9	77118987	T	C	0.28	-0.039	0.007	8.5E-09
rs2792990	9	125621610	C	G	0.86	0.054	0.008	1.2E-10
rs1927902	9	120518991	T	C	0.25	0.053	0.007	1.1E-14
rs118166957	9	8858043	T	C	0.16	0.068	0.008	2.0E-16
rs10761240	9	96361922	A	G	0.40	-0.043	0.006	2.1E-12
rs10758593	9	4292083	A	G	0.40	-0.036	0.006	4.9E-09
rs10756571	9	14534505	T	C	0.69	0.036	0.006	1.8E-08
rs7475916	10	77771194	C	G	0.35	-0.037	0.006	6.7E-09
rs224029	10	64519299	T	C	0.40	-0.039	0.006	2.5E-10
rs12251016	10	21821918	A	T	0.66	-0.039	0.006	3.9E-10
rs11001276	10	76825638	A	T	0.74	-0.038	0.007	2.5E-08
rs10825503	10	57177470	T	G	0.49	0.033	0.006	1.4E-08
rs72899452	11	45415577	T	C	0.06	0.074	0.012	1.0E-09
rs667730	11	83277325	T	C	0.58	0.033	0.006	2.3E-08
rs6589988	11	99126016	A	G	0.68	-0.038	0.006	4.7E-09
rs647905	11	121534938	T	C	0.54	0.033	0.006	2.9E-08
rs566673	11	66401373	T	G	0.54	-0.039	0.006	1.2E-10
rs56133505	11	72348039	A	G	0.54	0.041	0.006	5.6E-12
rs524859	11	66041079	A	G	0.36	-0.044	0.006	1.5E-12
rs4592425	11	62697813	T	G	0.70	0.040	0.006	4.3E-10
rs2221119	11	88598444	C	G	0.44	0.036	0.006	2.0E-09
rs214934	11	17193475	A	T	0.31	-0.038	0.006	3.2E-09
rs12790660	11	57667222	T	C	0.68	-0.040	0.006	4.5E-10
rs11605348	11	47606483	A	G	0.35	-0.045	0.006	7.0E-13
rs10898940	11	73455292	A	C	0.52	0.034	0.006	8.1E-09
rs1064939	11	118396331	A	T	0.98	0.130	0.020	2.2E-10
rs6606731	12	109982578	A	T	0.19	0.043	0.008	1.5E-08
rs61921611	12	66367726	T	C	0.69	-0.044	0.006	7.8E-12
rs4767645	12	118385788	T	G	0.46	-0.037	0.006	6.5E-10
rs324017	12	57487814	A	C	0.29	0.039	0.007	1.6E-09
rs28582096	12	123856998	A	G	0.21	-0.054	0.007	1.7E-13
rs2286729	12	6873818	A	G	0.09	0.070	0.011	5.4E-11
rs12310246	12	84700945	A	G	0.25	0.045	0.007	4.7E-11
rs1167132	12	43484487	T	C	0.39	0.035	0.006	8.7E-09
rs9563886	13	61720066	T	C	0.61	-0.034	0.006	3.1E-08
rs9540729	13	66947124	A	T	0.48	0.036	0.006	1.4E-09

rs9527083	13	53991125	A	G	0.67	-0.076	0.006	1.6E-32
rs7992992	13	54721699	A	G	0.13	0.051	0.009	1.2E-08
rs6562066	13	60532796	T	C	0.37	0.039	0.006	1.4E-10
rs2389631	13	96932868	A	C	0.67	-0.040	0.006	2.0E-10
rs1536053	13	111982291	T	C	0.32	-0.038	0.006	6.0E-09
rs11149313	13	85294881	A	G	0.73	0.040	0.007	2.4E-09
rs1031654	13	54382035	A	C	0.80	-0.051	0.007	3.9E-12
rs4981170	14	33412996	A	G	0.19	-0.054	0.008	7.3E-13
rs7402939	15	99183876	T	C	0.38	-0.036	0.006	5.2E-09
rs7168238	15	66709386	C	G	0.07	0.064	0.011	1.8E-08
rs715338	15	57215867	A	G	0.58	0.041	0.006	7.9E-12
rs4702	15	91426560	A	G	0.56	-0.048	0.006	6.8E-16
rs176644	15	89913632	T	G	0.40	0.035	0.006	9.5E-09
rs12917449	15	74331659	A	C	0.81	-0.042	0.008	3.0E-08
rs12912299	15	38897857	T	C	0.49	-0.043	0.006	4.4E-13
rs1038093	15	74012409	T	C	0.63	0.039	0.006	2.5E-10
rs9931543	16	56128782	T	C	0.74	0.048	0.007	1.1E-12
rs830716	16	12323509	C	G	0.71	0.045	0.007	8.7E-12
rs66674044	16	19904344	A	T	0.86	-0.060	0.009	2.2E-12
rs4788203	16	29978827	A	G	0.43	-0.035	0.006	6.3E-09
rs4238755	16	52746089	A	C	0.26	-0.043	0.007	2.3E-10
rs3902952	16	61647589	T	C	0.19	0.048	0.008	2.5E-10
rs35322724	16	77137324	A	C	0.58	0.049	0.006	3.8E-16
rs34214423	16	52303107	A	C	0.81	0.045	0.008	3.2E-09
rs3184470	16	715164	A	G	0.35	-0.038	0.006	9.7E-10
rs12924275	16	9191790	T	C	0.27	0.038	0.007	1.9E-08
rs1015438	16	51177517	A	G	0.19	0.058	0.008	2.5E-14
rs9889282	17	50259142	A	C	0.61	-0.042	0.006	4.7E-12
rs8076183	17	61024696	T	C	0.45	-0.038	0.006	2.8E-10
rs7214267	17	43157709	A	G	0.58	-0.044	0.006	5.1E-13
rs62068188	17	2400876	T	C	0.83	0.049	0.008	1.2E-09
rs4643373	17	47123423	T	C	0.70	0.041	0.007	1.6E-10
rs34490907	17	26933741	C	G	0.89	0.054	0.009	1.8E-08
rs11650304	17	46035001	C	G	0.93	0.067	0.012	1.2E-08
rs9964420	18	56824041	A	C	0.30	0.035	0.007	4.5E-08
rs60565673	18	52906830	T	G	0.62	-0.043	0.006	1.6E-12
rs12605642	18	31313965	T	G	0.49	0.035	0.006	2.1E-09
rs12454003	18	26315799	C	G	0.48	-0.035	0.006	4.9E-09
rs10502966	18	50748499	A	G	0.58	-0.039	0.006	8.5E-11
rs908668	19	56134038	T	C	0.21	0.050	0.007	1.4E-11

rs6510033	19	30710785	A	G	0.73	-0.037	0.007	4.7E-08
rs429358	19	45411941	T	C	0.85	0.046	0.008	2.1E-08
rs12983032	19	5073447	A	G	0.34	-0.043	0.006	1.1E-11
rs910187	20	45841052	A	G	0.37	-0.035	0.006	1.6E-08
rs76145129	20	62670427	T	G	0.12	-0.050	0.009	2.7E-08
rs742760	20	50985290	A	T	0.82	0.043	0.008	2.5E-08
rs6119267	20	31163914	C	G	0.69	-0.060	0.006	2.3E-20
rs6019663	20	47774512	T	C	0.29	0.040	0.007	6.5E-10
rs2867690	20	41972028	T	C	0.18	0.042	0.008	3.7E-08
rs2838787	21	46539725	A	G	0.39	-0.036	0.006	7.7E-09
rs11090039	22	41496800	A	G	0.29	0.039	0.007	1.8E-09

Note: SNP, single nucleotide polymorphism; Chr, chromosome; Pos, position; EA, effect allele; OA, other allele; EAF, effect allele frequence; Se, standard error.

Table S2. Genome-wide significant ($p<5\times10^{-8}$) single nucleotide polymorphisms that were used as instruments for sleep duration.

SNP	Chr	Pos	EA	OA	EAF	Beta	Se	*P*-value
rs915416	1	34731984	C	G	0.29	0.138	0.966	2.1E-12
rs269054	1	57864304	A	T	0.42	0.126	0.666	1.5E-07
rs61796569	1	66476437	T	C	0.27	0.144	0.828	4.5E-09
rs12567114	1	98527951	A	G	0.28	0.138	0.846	2.1E-09
rs7556815	2	114085785	A	G	0.22	0.150	2.352	1.8E-54
rs12611523	2	139195328	A	G	0.55	0.126	0.702	2.6E-08
rs4128364	2	147612734	C	T	0.34	0.132	0.780	3.9E-09
rs4538155	2	157040773	T	C	0.65	0.132	0.762	5.8E-09
rs11885663	2	166944004	T	C	0.25	0.144	0.864	3.5E-09
rs10173260	2	210377845	C	T	0.61	0.126	0.618	1.6E-06
rs374153	2	40382712	C	T	0.16	0.174	0.990	8.5E-09
rs75539574	2	58871658	C	A	0.09	0.174	1.548	3.0E-19
rs72804080	2	59358659	G	A	0.15	0.192	1.068	2.9E-08
rs62120041	2	9185564	T	C	0.93	0.270	1.380	3.2E-07
rs7644809	3	107564459	T	C	0.42	0.126	0.624	1.0E-06
rs13088093	3	135838598	G	T	0.34	0.132	0.906	8.4E-12
rs112230981	3	55879269	A	G	0.95	0.282	0.990	5.1E-04
rs17732997	3	70470834	C	G	0.57	0.126	0.636	5.2E-07
rs13109404	4	102896591	T	G	0.93	0.264	1.716	9.5E-11
rs2192528	4	18327896	A	G	0.48	0.126	0.666	9.9E-08
rs17427571	4	82254908	A	G	0.68	0.132	0.726	7.4E-08
rs35531607	4	92533225	C	T	0.47	0.126	0.708	2.2E-08
rs56372231	5	102321905	T	C	0.33	0.132	0.888	1.8E-11
rs180769	5	135615615	T	C	0.43	0.126	0.678	1.1E-07
rs365663	5	1428883	A	G	0.55	0.126	0.696	3.2E-08
rs151014368	5	176751059	A	G	0.21	0.150	0.756	4.1E-07
rs460692	5	3126584	C	T	0.14	0.204	1.206	3.2E-09
rs34556183	6	28584775	A	G	0.72	0.150	0.990	2.5E-11
rs80193650	6	33464363	G	A	0.16	0.180	0.774	2.3E-05
rs113113059	6	43160375	T	C	0.78	0.156	0.828	8.5E-08
rs9382445	6	54937974	T	C	0.62	0.132	0.846	6.3E-11
rs2231265	6	89790201	G	A	0.77	0.150	0.930	4.3E-10
rs9345234	6	93162639	C	A	0.58	0.126	0.714	2.1E-08
rs2079070	7	114126432	C	G	0.27	0.154	1.053	7.5E-12
rs7806045	7	132610266	T	C	0.76	0.144	0.894	1.1E-09
rs34731055	7	2106928	T	C	0.18	0.162	1.044	1.1E-10
rs73219758	8	14279446	G	A	0.71	0.138	0.906	4.6E-11

rs330088	8	9149746	C	T	0.55	0.126	-0.666	1.1E-07
rs1776776	9	140497072	T	C	0.87	0.198	1.104	2.7E-08
rs10973207	9	37100525	T	G	0.16	0.174	1.050	1.3E-09
rs11190970	10	103128332	G	A	0.80	0.162	0.864	6.5E-08
rs7915425	10	125016501	T	C	0.18	0.162	1.014	8.2E-10
rs12246842	10	21830580	A	G	0.46	0.126	0.726	7.2E-09
rs10761674	10	64618340	C	T	0.48	0.126	0.696	2.5E-08
rs1939455	11	101520886	G	T	0.88	0.198	1.050	1.7E-07
rs7115226	11	113408518	A	C	0.07	0.261	1.594	1.7E-09
rs1263056	11	116576415	A	G	0.52	0.126	0.768	1.3E-09
rs7951019	11	118358027	G	T	0.03	0.391	2.213	1.2E-08
rs1057703	11	122830251	G	T	0.15	0.186	0.984	9.3E-08
rs1517572	11	28829882	C	A	0.58	0.126	0.810	1.7E-10
rs4592416	11	43800474	G	A	0.46	0.126	0.798	2.3E-10
rs11602180	11	48162453	C	T	0.84	0.168	0.918	5.8E-08
rs174560	11	61581764	C	T	0.31	0.138	0.792	5.8E-09
rs12791153	11	80685181	T	A	0.08	0.234	1.320	2.6E-08
rs1553132	11	88297740	G	A	0.26	0.144	0.852	5.1E-09
rs4767550	12	117951150	G	A	0.41	0.126	0.822	1.1E-10
rs34354917	12	38764559	C	A	0.71	0.138	0.666	1.8E-06
rs6575005	14	26954078	T	C	0.76	0.144	0.858	4.3E-09
rs10483350	14	29816155	G	A	0.20	0.156	0.858	6.4E-08
rs61985058	14	60233841	T	C	0.14	0.180	0.936	2.1E-07
rs55658675	14	65554638	C	T	0.65	0.132	0.720	4.9E-08
rs11621908	14	78495761	C	T	0.92	0.228	1.266	3.4E-08
rs8038326	15	47989799	A	G	0.73	0.138	0.906	1.2E-10
rs11643715	16	23909538	G	C	0.29	0.144	0.660	5.6E-06
rs9940646	16	53800629	C	G	0.58	0.137	1.017	1.2E-13
rs8050478	16	56120461	G	A	0.50	0.126	0.948	3.4E-14
rs3095508	16	6550400	C	A	0.59	0.126	0.702	3.4E-08
rs205024	17	11227352	T	C	0.38	0.126	0.762	3.0E-09
rs1991556	17	44083402	G	A	0.77	0.163	0.994	1.0E-09
rs9903973	17	50571227	C	T	0.47	0.132	0.750	1.2E-08
rs7503199	17	8134275	C	T	0.73	0.138	0.810	6.9E-09
rs12607679	18	53059748	T	C	0.74	0.156	1.208	8.3E-15
rs10421649	19	9942262	A	T	0.56	0.126	0.906	5.8E-13
rs2072727	20	43538733	T	C	0.44	0.126	0.696	3.8E-08

Note: SNP, single nucleotide polymorphism; Chr, chromosome; Pos, position; EA, effect allele; OA, other allele; EAF, effect allele frequence; Se, standard error.

Table S3. Genome-wide significant ($p<5\times10^{-8}$) single nucleotide polymorphisms that were used as instruments for getting up.

SNP	Chr	Pos	EA	OA	EAF	Beta	Se	*P*-value
rs61773374	1	7858108	G	A	0.20	0.016	0.002	3.4E-14
rs301806	1	8482078	C	T	0.42	-0.010	0.002	4.7E-08
rs77576965	1	15916734	T	C	0.27	0.011	0.002	1.9E-08
rs12752290	1	21535330	C	T	0.44	0.012	0.002	1.0E-12
rs113240734	1	77699071	A	G	0.16	0.019	0.002	2.2E-16
rs75650221	1	174421994	T	C	0.04	0.039	0.005	3.9E-18
rs4652514	1	180529542	C	T	0.30	-0.011	0.002	9.9E-09
rs12736689	1	182549729	C	T	0.03	0.052	0.005	1.7E-24
rs76048411	2	4650079	T	C	0.48	0.011	0.002	8.5E-10
rs2053457	2	44572164	C	T	0.25	-0.016	0.002	1.3E-15
rs10180284	2	50716016	T	C	0.48	0.014	0.002	4.9E-08
rs4671328	2	58935282	T	G	0.45	0.011	0.002	1.1E-09
rs10175975	2	59429807	T	C	0.18	0.013	0.002	3.3E-09
rs13393656	2	70119255	A	C	0.24	0.011	0.002	2.1E-08
rs406952	2	76307345	C	T	0.38	0.010	0.002	1.2E-08
rs4853283	2	77156402	G	A	0.44	-0.014	0.002	1.8E-16
rs1606803	2	88933316	T	C	0.29	0.012	0.002	3.2E-10
rs116298301	2	239177686	T	C	0.03	-0.017	0.002	2.4E-11
rs4483990	2	239423098	C	A	0.15	-0.017	0.002	6.4E-13
rs13116306	4	10727697	T	C	0.42	-0.010	0.002	1.8E-08
rs10470887	4	92567442	G	A	0.49	-0.010	0.002	4.0E-08
rs9995419	4	158607383	A	G	0.24	0.011	0.002	1.5E-08
rs79751662	4	171014655	C	G	0.11	-0.016	0.003	2.3E-08
rs1459192	5	63944519	T	C	0.33	-0.010	0.002	2.5E-08
rs12515274	5	87655662	A	G	0.25	-0.012	0.002	5.4E-10
rs4958316	5	151942251	A	C	0.29	0.015	0.002	3.7E-15
rs553108	6	31840455	A	G	0.42	0.010	0.002	3.1E-08
rs2653349	6	55142337	A	G	0.21	0.023	0.002	8.5E-29
rs9399613	6	147986024	T	C	0.29	-0.011	0.002	1.3E-08
rs3735478	7	44800176	T	G	0.29	-0.011	0.002	1.6E-08
rs2944822	7	71795592	T	C	0.45	0.010	0.002	4.4E-09
rs16917522	8	53136442	C	T	0.18	0.014	0.002	1.1E-09
rs72663537	8	76700618	G	T	0.13	-0.014	0.003	2.8E-08
rs77641763	9	140265782	T	C	0.12	-0.018	0.003	2.0E-11
rs4962716	10	126685867	T	C	0.13	-0.016	0.003	1.8E-09
rs11229264	11	57909086	A	G	0.30	-0.012	0.002	1.4E-10
rs7297799	12	34260142	T	C	0.40	-0.013	0.002	1.2E-12

rs2193749	12	46134812	T	C	0.49	-0.010	0.002	1.8E-08
rs6581138	12	57744864	A	G	0.24	0.013	0.002	2.3E-10
rs1017168	12	107435405	A	C	0.36	-0.010	0.002	2.6E-08
rs74643199	12	116772891	T	A	0.15	-0.014	0.002	1.2E-08
rs4884166	13	55769145	A	G	0.20	-0.012	0.002	1.2E-08
rs7332608	13	77570677	G	A	0.03	-0.031	0.005	4.6E-11
rs6575012	14	89056699	A	G	0.48	-0.010	0.002	2.6E-08
rs3935182	15	78094807	G	C	0.42	0.012	0.002	6.8E-12
rs1420607	16	49149191	A	G	0.27	0.013	0.002	1.6E-11
rs11642015	16	53802494	T	C	0.40	0.011	0.002	6.2E-11
rs1949072	16	56006108	A	G	0.43	0.010	0.002	2.5E-08
rs17822102	16	60650143	G	A	0.34	0.011	0.002	9.4E-10
rs11643192	16	72214276	A	C	0.39	-0.011	0.002	1.3E-09
rs4790352	17	2578550	G	A	0.08	-0.018	0.003	8.8E-09
rs3760185	17	17401736	T	C	0.24	-0.011	0.002	4.5E-08
rs7222039	17	38165541	T	C	0.45	0.010	0.002	3.4E-08
rs12150229	17	44015446	G	A	0.22	0.012	0.002	3.5E-09
rs77556405	17	46463909	A	G	0.17	0.017	0.002	1.6E-13
rs12601968	17	50296459	T	G	0.33	-0.011	0.002	1.2E-09
rs4395148	18	31690668	T	A	0.31	-0.010	0.002	2.4E-08
rs8182491	19	42712024	T	C	0.10	-0.017	0.003	1.2E-09
rs3746601	20	30662805	C	A	0.36	0.010	0.002	2.9E-08
rs11697690	20	31477981	C	T	0.47	0.010	0.002	2.3E-08
rs74555583	20	51332268	A	G	0.09	-0.017	0.003	3.0E-08

Note: SNP, single nucleotide polymorphism; Chr, chromosome; Pos, position; EA, effect allele; OA, other allele; EAF, effect allele frequence; Se, standard error;getting up, The ease of getting up in the morning.

Table S4. Genome-wide significant ($p<5\times10^{-8}$) single nucleotide polymorphisms that were used as instruments for morningness.

SNP	Chr	Pos	EA	OA	EAF	Beta	Se	*P*-value
rs61773390	1	7884525	T	G	0.20	0.029	0.003	2.8E-27
rs12065331	1	14507831	T	C	0.31	-0.015	0.002	1.5E-10
rs17448682	1	15966713	T	C	0.23	0.018	0.003	4.0E-13
rs7543480	1	20014827	T	C	0.34	0.016	0.002	6.9E-13
rs12140153	1	62579891	T	G	0.09	-0.027	0.004	4.6E-12
rs11208844	1	66851147	A	G	0.14	-0.017	0.003	2.8E-08
rs11162296	1	77700196	C	G	0.16	0.036	0.003	1.5E-34
rs17416934	1	79874811	T	C	0.62	0.013	0.002	5.9E-09
rs74802342	1	91078046	A	G	0.11	-0.019	0.003	2.1E-08
rs72720396	1	91191582	A	G	0.77	-0.022	0.003	3.3E-18
rs7522677	1	96470261	T	C	0.21	0.015	0.003	7.9E-09
rs12139650	1	97598100	T	G	0.81	0.015	0.003	2.2E-08
rs10494041	1	110031990	C	G	0.83	0.020	0.003	1.0E-12
rs35461065	1	115060826	T	C	0.54	-0.012	0.002	8.4E-09
rs2794682	1	150320847	T	C	0.40	0.021	0.002	3.9E-22
rs75650221	1	174421994	T	C	0.04	0.034	0.006	8.9E-10
rs13306728	1	179312559	A	G	0.92	0.030	0.004	6.2E-14
rs509476	1	182573227	T	C	0.03	0.099	0.006	7.4E-56
rs12746073	1	183679912	T	C	0.53	0.012	0.002	1.7E-08
rs12025393	1	193341118	A	G	0.27	-0.014	0.002	1.8E-08
rs16839841	1	196353384	T	G	0.92	0.022	0.004	2.7E-08
rs13011556	2	4651923	C	G	0.76	-0.016	0.003	7.7E-11
rs2712056	2	23959131	T	C	0.18	0.018	0.003	1.6E-10
rs848552	2	36700580	C	G	0.47	-0.013	0.002	1.1E-09
rs62135536	2	44326028	T	C	0.03	0.035	0.006	1.4E-08
rs2592199	2	44710169	C	G	0.77	0.022	0.003	1.4E-14
rs10495976	2	49750698	A	T	0.61	-0.017	0.002	1.0E-13
rs1520524	2	50525628	T	C	0.10	0.025	0.004	2.7E-12
rs10193431	2	53725767	T	C	0.53	0.013	0.002	4.4E-09
rs17049270	2	58192905	T	C	0.10	-0.020	0.004	2.0E-08
rs10175975	2	59429807	T	C	0.18	0.018	0.003	1.9E-10
rs4672440	2	61616653	T	G	0.35	0.016	0.002	5.1E-13
rs113851554	2	66750564	T	G	0.05	-0.028	0.005	1.3E-08
rs2706762	2	70488470	T	C	0.15	-0.019	0.003	3.0E-10
rs7586062	2	77305160	C	G	0.47	-0.022	0.002	3.1E-24
rs10190053	2	77916762	A	C	0.37	-0.013	0.002	6.5E-09
rs75863239	2	105312975	T	C	0.91	-0.023	0.004	3.2E-08

rs62172117	2	144168667	A	G	0.36	-0.019	0.002	1.4E-17
rs7579662	2	161915810	A	G	0.39	-0.012	0.002	2.2E-08
rs13004345	2	174037347	T	C	0.65	-0.012	0.002	3.8E-08
rs11677484	2	191578172	T	G	0.25	0.015	0.002	6.2E-10
rs4850712	2	197301425	T	G	0.32	0.013	0.002	9.4E-09
rs6716898	2	198944271	A	G	0.48	0.021	0.002	2.3E-22
rs184033703	2	206956138	A	G	0.06	0.032	0.005	9.6E-10
rs35333999	2	239161957	T	C	0.04	-0.047	0.005	5.1E-19
rs77942338	2	239194693	T	C	0.97	-0.056	0.007	1.4E-16
rs11900963	2	239308049	A	T	0.91	0.045	0.004	3.2E-32
rs62182135	2	240267305	A	C	0.33	-0.013	0.002	7.8E-09
rs17786957	3	2550093	C	G	0.16	-0.018	0.003	6.1E-10
rs7428484	3	23330101	A	G	0.67	0.013	0.002	2.0E-08
rs62263597	3	50131691	A	G	0.08	0.028	0.004	4.8E-13
rs7652260	3	50571585	C	G	0.84	0.017	0.003	2.0E-08
rs67000219	3	71557581	T	C	0.11	-0.019	0.003	2.7E-08
rs9876864	3	77208521	A	T	0.41	0.016	0.002	9.2E-14
rs55753638	3	85644482	T	C	0.88	-0.022	0.003	2.9E-11
rs1800828	3	113891549	C	G	0.75	0.014	0.002	5.3E-09
rs6799356	3	123148362	A	C	0.75	0.014	0.003	4.1E-08
rs2699869	3	133032892	A	C	0.45	0.013	0.002	5.1E-09
rs1109088	3	138154795	A	G	0.57	0.013	0.002	2.6E-09
rs6769642	3	160763469	A	C	0.48	0.016	0.002	2.8E-13
rs3850174	3	172364093	A	T	0.25	-0.014	0.002	9.1E-09
rs6443788	3	181966190	A	C	0.29	-0.015	0.002	1.0E-09
rs6443810	3	182262672	C	G	0.69	0.015	0.002	2.5E-10
rs6778003	3	185991970	T	G	0.29	0.016	0.002	4.9E-12
rs7617588	3	186079990	T	C	0.86	-0.021	0.003	3.5E-11
rs9683585	4	2702804	C	G	0.56	-0.012	0.002	1.8E-08
rs56040212	4	16435084	A	G	0.24	-0.014	0.003	2.2E-08
rs28634184	4	62873419	T	C	0.25	-0.014	0.002	2.3E-08
rs57180764	4	83239773	A	G	0.77	0.017	0.003	3.2E-11
rs4241964	4	137053959	T	G	0.52	-0.015	0.002	3.5E-12
rs3797051	5	63860141	T	C	0.76	0.015	0.003	1.5E-09
rs12657776	5	87661593	A	G	0.75	0.019	0.002	3.5E-14
rs304137	5	88169652	A	G	0.52	0.016	0.002	3.1E-14
rs286808	5	107459376	T	C	0.47	0.013	0.002	3.9E-09
rs2910032	5	152540354	T	C	0.52	0.019	0.002	6.2E-18
rs42210	5	166408788	C	G	0.71	-0.014	0.002	3.5E-09
rs335433	5	176867207	T	C	0.46	-0.013	0.002	2.3E-09

rs9395520	6	13183523	T	C	0.30	0.020	0.002	1.6E-18
rs9295795	6	29145623	T	C	0.94	-0.026	0.005	1.9E-08
rs486416	6	31856070	A	G	0.64	-0.013	0.002	6.1E-09
rs734597	6	50836279	A	G	0.17	0.016	0.003	1.6E-08
rs2653349	6	55142337	A	G	0.21	0.031	0.003	1.0E-32
rs2881955	6	72479263	T	C	0.28	0.016	0.002	5.6E-11
rs9375352	6	98739938	A	T	0.27	0.014	0.002	2.0E-08
rs4557564	6	110232051	A	G	0.93	0.025	0.004	2.1E-09
rs6935086	6	128944677	T	C	0.89	0.020	0.003	5.9E-09
rs9479402	6	153135339	T	C	0.99	-0.102	0.01	1.3E-22
rs9348050	6	166263488	T	C	0.49	0.013	0.002	2.0E-09
rs16873715	7	8560454	A	T	0.76	-0.015	0.003	4.4E-08
rs56382918	7	24067993	T	C	0.74	0.018	0.002	1.1E-12
rs10236197	7	32291761	T	C	0.63	0.014	0.002	2.0E-10
rs56049037	7	32947201	A	G	0.29	-0.017	0.002	2.3E-12
rs4245555	7	50661409	T	C	0.59	-0.018	0.002	2.0E-15
rs2138759	7	71823436	A	G	0.30	0.014	0.002	1.7E-09
rs2922966	7	96455598	A	G	0.84	0.025	0.003	1.1E-17
rs202157	7	101637753	T	C	0.70	-0.018	0.002	1.2E-14
rs112613078	7	102433678	A	G	0.81	-0.025	0.003	1.5E-20
rs10262462	7	114180062	A	G	0.40	-0.015	0.002	3.2E-12
rs6978514	7	115684409	T	C	0.56	-0.013	0.002	4.9E-08
rs2971970	7	133643778	T	G	0.22	0.017	0.003	2.2E-11
rs35748596	8	4825443	T	G	0.64	-0.016	0.002	2.4E-12
rs34344642	8	31765586	T	G	0.08	-0.024	0.004	2.9E-09
rs12541362	8	33584661	A	T	0.65	-0.018	0.002	5.6E-15
rs1919346	8	35211457	A	G	0.53	0.012	0.002	1.3E-08
rs11988076	8	53128629	A	G	0.84	-0.019	0.003	1.8E-11
rs6472936	8	76639759	T	C	0.78	0.018	0.003	5.2E-12
rs1110275	8	86854332	T	C	0.91	0.020	0.004	4.1E-08
rs34578339	8	89440842	A	T	0.84	-0.016	0.003	2.8E-08
rs72673588	8	93356390	C	G	0.82	-0.017	0.003	1.3E-09
rs3100052	8	101967139	A	G	0.39	0.013	0.002	1.3E-08
rs2737245	8	116658583	T	G	0.28	0.018	0.002	2.9E-14
rs1323591	9	8456020	T	C	0.32	-0.016	0.002	2.1E-12
rs2291589	9	37079661	T	G	0.62	0.018	0.002	1.7E-16
rs77598468	9	76484268	A	C	0.03	-0.042	0.006	1.1E-12
rs4565536	9	85189088	A	C	0.50	-0.012	0.002	5.0E-08
rs10797119	9	92202495	T	C	0.46	-0.013	0.002	2.8E-09
rs28365587	9	131959615	A	G	0.51	-0.013	0.002	3.6E-09

rs10448340	9	139320069	T	G	0.68	-0.013	0.002	9.1E-09
rs28458909	9	140257189	T	C	0.12	-0.030	0.004	1.9E-16
rs1750785	10	776150	A	G	0.26	0.014	0.002	3.5E-09
rs9416744	10	60567937	A	C	0.26	0.017	0.002	1.5E-12
rs7910164	10	72852735	A	G	0.64	0.012	0.002	4.8E-08
rs76518095	10	131149976	T	C	0.08	0.023	0.004	1.0E-08
rs9795439	11	1483543	A	G	0.20	0.018	0.003	6.7E-09
rs925947	11	27667367	T	G	0.20	0.016	0.003	9.6E-10
rs12799529	11	30408552	T	C	0.76	0.018	0.003	5.1E-13
rs11032362	11	33759092	A	G	0.09	0.031	0.004	3.5E-17
rs34239319	11	43902681	T	G	0.10	0.024	0.004	6.5E-12
rs11039308	11	47622412	A	G	0.41	0.014	0.002	4.9E-11
rs3168135	11	58386177	A	G	0.24	-0.017	0.003	3.6E-12
rs4008953	11	66660949	A	G	0.71	-0.016	0.002	3.2E-11
rs4237555	11	92725803	T	C	0.53	0.012	0.002	4.2E-08
rs4936290	11	114009255	A	C	0.65	-0.013	0.002	1.2E-08
rs577924	11	122135107	T	C	0.53	0.012	0.002	2.1E-08
rs1174510	12	17093317	A	G	0.62	0.012	0.002	4.9E-08
rs11611435	12	24089322	T	C	0.55	0.012	0.002	4.0E-08
rs7313852	12	38908389	A	G	0.44	-0.025	0.002	1.0E-29
rs11183201	12	46170982	T	C	0.49	-0.017	0.002	1.6E-14
rs671255	12	52189566	A	G	0.75	-0.014	0.002	1.4E-08
rs7299922	12	54702519	A	G	0.63	0.013	0.002	8.6E-09
rs7138306	12	63634487	A	G	0.47	0.013	0.002	8.9E-10
rs7488974	12	90442001	A	G	0.40	0.016	0.002	1.8E-13
rs10861694	12	107405922	T	C	0.56	0.014	0.002	2.6E-10
rs4102203	12	120976371	T	C	0.89	0.024	0.003	7.8E-12
rs61963123	13	42512639	T	C	0.78	0.017	0.003	2.3E-10
rs9597250	13	56303709	A	C	0.19	-0.019	0.003	2.9E-12
rs2593487	13	69903058	A	G	0.34	-0.014	0.002	2.9E-10
rs9565309	13	77577027	T	C	0.97	0.066	0.006	1.3E-28
rs7337911	13	94111843	A	G	0.75	0.015	0.002	1.0E-09
rs9521184	13	109782568	T	C	0.49	0.013	0.002	8.3E-10
rs56376592	14	57378912	A	C	0.82	0.017	0.003	1.4E-09
rs4899502	14	74669893	A	G	0.69	-0.016	0.002	1.6E-11
rs12432176	14	101021218	A	C	0.38	0.013	0.002	1.5E-08
rs2701524	15	37383688	T	C	0.59	0.013	0.002	5.4E-09
rs59986227	15	48009263	C	G	0.75	-0.015	0.002	5.4E-09
rs12442008	15	53725112	T	C	0.25	0.014	0.002	3.4E-08
rs11852820	15	101150435	C	G	0.66	-0.014	0.002	5.8E-10

rs2304467	16	8988777	C	G	0.60	-0.012	0.002	3.9E-08
rs7196720	16	24534662	T	C	0.49	0.013	0.002	4.3E-09
rs12927162	16	52684916	A	G	0.72	0.028	0.002	2.1E-32
rs1421085	16	53800954	T	C	0.60	-0.023	0.002	1.4E-25
rs2398144	16	56352854	A	C	0.39	-0.021	0.002	2.4E-21
rs8044054	16	60628436	T	C	0.39	0.015	0.002	3.9E-12
rs17604349	16	72210865	A	G	0.18	-0.022	0.003	1.2E-15
rs2518022	17	8057367	T	C	0.09	0.032	0.004	1.5E-16
rs2232839	17	17399635	T	C	0.21	-0.025	0.003	2.4E-21
rs9915731	17	30599555	A	T	0.27	-0.013	0.002	2.8E-08
rs225289	17	33928945	T	C	0.17	0.016	0.003	1.2E-08
rs72828815	17	42994794	T	C	0.18	0.016	0.003	6.1E-09
rs117974417	17	46156510	C	G	0.85	-0.018	0.003	1.5E-09
rs6504758	17	50208366	A	G	0.46	-0.014	0.002	9.8E-11
rs9898091	17	54173599	T	C	0.96	0.038	0.006	6.9E-12
rs8072058	17	55734198	A	T	0.78	-0.015	0.003	1.4E-08
rs17682747	17	61181112	A	G	0.23	0.014	0.003	4.7E-08
rs2916142	17	65482064	T	C	0.46	0.015	0.002	7.6E-12
rs10491171	17	68414995	C	G	0.87	0.019	0.003	3.6E-09
rs487952	17	74898300	A	G	0.57	0.013	0.002	4.4E-09
rs974552	18	5186164	A	G	0.81	-0.022	0.003	1.4E-15
rs1013987	18	22630836	T	C	0.40	-0.015	0.002	1.4E-11
rs4239386	18	31664710	A	T	0.33	-0.019	0.002	2.1E-17
rs12969848	18	38152835	T	C	0.53	0.017	0.002	1.5E-15
rs989885	18	38464855	A	G	0.94	-0.024	0.004	3.8E-08
rs9956387	18	44773382	A	T	0.50	-0.014	0.002	1.4E-10
rs17596722	18	53154167	T	C	0.20	-0.018	0.003	3.2E-11
rs9964420	18	56824041	A	C	0.30	-0.022	0.002	4.0E-21
rs11152350	18	60240352	A	C	0.47	-0.015	0.002	1.1E-12
rs9958145	18	64363800	A	G	0.20	-0.015	0.003	4.3E-08
rs10402849	19	2695661	T	C	0.20	0.016	0.003	6.7E-09
rs3843751	19	10748121	T	C	0.66	0.013	0.002	6.3E-09
rs9636202	19	18449238	A	G	0.27	-0.013	0.002	3.5E-08
rs12481462	20	14728972	T	C	0.37	0.012	0.002	4.4E-08
rs78095690	20	16239683	T	C	0.56	-0.013	0.002	5.8E-09
rs6131942	20	17348608	A	G	0.42	-0.015	0.002	7.6E-12
rs1737893	20	31051699	T	C	0.38	-0.014	0.002	1.4E-09
rs2072727	20	43538733	T	C	0.44	0.012	0.002	3.5E-08
rs695459	22	28848278	T	C	0.39	-0.013	0.002	6.6E-09
rs28580373	22	35847288	A	G	0.23	0.014	0.003	3.3E-08

rs11705370	22	40555993	A	T	0.60	-0.019	0.002	3.4E-15
rs4822107	22	42704834	A	G	0.51	0.012	0.002	2.2E-08
rs2294203	22	45738487	A	G	0.68	0.013	0.002	3.0E-08

Note: SNP, single nucleotide polymorphism; Chr, chromosome; Pos, position; EA, effect allele; OA, other allele; EAF, effect allele frequence; Se, standard error.

Table S5. Genome-wide significant (p<5×10^{-8}) single nucleotide polymorphisms that were used as instruments for snoring.

SNP	Chr	Pos	EA	OA	EAF	Beta	Se	*P*-value
rs35915391	1	87781737	C	G	0.35	0.971	0.005	2.2E-05
rs35562935	1	96689205	A	G	0.07	1.055	0.009	1.7E-05
rs72906130	2	157076893	G	C	0.13	1.057	0.007	3.5E-14
rs9309771	3	77593064	A	G	0.45	1.030	0.005	5.7E-09
rs34811474	4	25408838	A	G	0.23	0.962	0.006	4.2E-08
rs6855873	4	42539270	T	C	0.24	1.033	0.006	2.1E-05
rs2307111	5	75003678	C	T	0.39	0.966	0.005	1.3E-08
rs10062026	5	90052289	A	G	0.36	0.971	0.005	2.1E-05
rs947612	6	73738661	G	A	0.25	1.034	0.006	9.9E-06
rs17060460	6	100827834	G	A	0.23	1.034	0.006	1.4E-08
rs9389081	6	133815465	A	T	0.08	0.950	0.009	4.4E-08
rs2981329	8	34985791	C	T	0.26	1.034	0.006	7.7E-09
rs7007887	8	71555913	T	C	0.43	1.038	0.005	9.9E-14
rs4523230	8	78234143	A	T	0.28	0.967	0.006	1.7E-09
rs1016013	9	97476484	A	G	0.40	0.969	0.005	7.2E-10
rs11256034	10	9086147	T	C	0.25	1.039	0.006	4.7E-11
rs2049045	11	27694241	C	G	0.19	0.959	0.006	8.9E-11
rs10878269	12	65791463	T	C	0.36	1.038	0.005	3.8E-13
rs12427782	13	40745813	T	G	0.49	0.971	0.005	5.8E-09
rs2762049	13	50822363	C	G	0.39	1.035	0.005	8.7E-09
rs592333	13	51340315	G	A	0.44	1.042	0.005	1.1E-13
rs2664299	14	99742187	C	T	0.42	0.967	0.005	4.5E-08
rs9933881	16	1740691	C	T	0.07	1.056	0.009	8.9E-09
rs732172	16	31050033	T	C	0.37	1.032	0.005	1.3E-09
rs8047587	16	53798622	T	G	0.44	1.031	0.005	2.1E-09
rs12449843	17	2058207	A	G	0.38	0.968	0.005	4.4E-10
rs1641511	17	7559677	G	A	0.24	1.034	0.006	1.4E-08
rs57222984	17	43758898	G	A	0.24	1.039	0.006	3.0E-11
rs2924251	17	46338677	A	G	0.33	1.031	0.005	5.2E-09
rs180107	17	67930772	A	T	0.41	0.971	0.005	3.9E-09
rs4987719	18	60960310	T	C	0.03	1.078	0.014	3.6E-08
rs10415992	19	32181118	G	C	0.09	0.952	0.009	8.1E-09
rs34107769	20	46306828	C	T	0.29	0.969	0.005	9.9E-09
rs6099273	20	55347828	T	C	0.25	1.033	0.006	1.2E-08

Note: SNP, single nucleotide polymorphism; Chr, chromosome; Pos, position; EA, effect allele; OA, other allele; EAF, effect allele frequence; Se, standard error.

TableS6. Genetic data for sleep traits and CAD in patients with diabetes.

Exposure	SNP	EA	OA	Exposure			Outcome		
				Beta	Se	*P*-value	Beta	Se	*P*-value
insomnia	rs9964420	A	C	0.04	0.01	4.5E-08	-0.01	0.03	7.5E-01
insomnia	rs9931543	T	C	0.05	0.01	1.1E-12	-0.04	0.03	2.0E-01
insomnia	rs9889282	A	C	-0.04	0.01	4.7E-12	0.00	0.03	8.8E-01
insomnia	rs9563886	T	C	-0.03	0.01	3.1E-08	-0.04	0.03	1.5E-01
insomnia	rs9540729	A	T	0.04	0.01	1.4E-09	0.01	0.03	7.7E-01
insomnia	rs9527083	A	G	-0.08	0.01	1.6E-32	-0.10	0.03	2.7E-04
insomnia	rs9394502	T	C	-0.05	0.01	7.8E-18	0.00	0.03	9.7E-01
insomnia	rs9373590	A	T	0.04	0.01	2.2E-11	0.01	0.03	6.1E-01
insomnia	rs910187	A	G	-0.03	0.01	1.6E-08	-0.03	0.03	2.6E-01
insomnia	rs908668	T	C	0.05	0.01	1.4E-11	0.04	0.03	2.3E-01
insomnia	rs874168	T	C	0.03	0.01	7.9E-09	0.00	0.03	9.0E-01
insomnia	rs871994	A	C	0.04	0.01	5.5E-09	0.00	0.03	8.9E-01
insomnia	rs830716	C	G	0.04	0.01	8.7E-12	0.03	0.03	3.1E-01
insomnia	rs823247	T	C	-0.04	0.01	5.2E-10	-0.01	0.03	6.1E-01
insomnia	rs8180817	C	G	-0.05	0.01	1.8E-16	-0.06	0.03	3.8E-02
insomnia	rs8180457	T	C	-0.06	0.01	1.1E-11	0.03	0.04	3.7E-01
insomnia	rs8076183	T	C	-0.04	0.01	2.8E-10	-0.01	0.03	6.7E-01
insomnia	rs7992992	A	G	0.05	0.01	1.2E-08	0.01	0.04	7.6E-01
insomnia	rs77641763	T	C	0.07	0.01	6.5E-15	0.02	0.04	6.2E-01
insomnia	rs7625896	A	G	0.04	0.01	5.3E-09	-0.01	0.03	7.7E-01
insomnia	rs7615602	C	G	-0.04	0.01	2.6E-09	-0.02	0.03	5.0E-01
insomnia	rs76145129	T	G	-0.05	0.01	2.7E-08	0.00	0.04	9.5E-01
insomnia	rs7599697	T	C	-0.04	0.01	5.0E-09	0.04	0.03	1.8E-01
insomnia	rs75932578	T	C	-0.04	0.01	4.1E-08	0.00	0.03	9.7E-01
insomnia	rs7571486	A	G	-0.04	0.01	1.4E-08	0.00	0.03	9.0E-01
insomnia	rs75452188	A	G	0.05	0.01	1.6E-08	0.08	0.04	5.6E-02
insomnia	rs7475916	C	G	-0.04	0.01	6.7E-09	0.03	0.03	3.7E-01
insomnia	rs742760	A	T	0.04	0.01	2.5E-08	-0.02	0.03	4.8E-01
insomnia	rs7402939	T	C	-0.04	0.01	5.2E-09	0.00	0.03	8.7E-01
insomnia	rs73671843	A	G	-0.06	0.01	5.5E-10	-0.07	0.04	8.5E-02
insomnia	rs72899452	T	C	0.07	0.01	1.0E-09	-0.05	0.05	3.7E-01
insomnia	rs72820274	A	G	0.03	0.01	1.3E-08	0.03	0.03	1.9E-01
insomnia	rs728017	A	G	-0.03	0.01	9.5E-09	0.00	0.03	8.6E-01
insomnia	rs72773790	T	C	0.04	0.01	3.7E-09	0.01	0.03	8.1E-01
insomnia	rs72657797	T	C	-0.06	0.01	1.5E-12	-0.01	0.03	8.6E-01
insomnia	rs7214267	A	G	-0.04	0.01	5.1E-13	-0.04	0.03	1.8E-01
insomnia	rs7168238	C	G	0.06	0.01	1.8E-08	0.06	0.05	2.3E-01

insomnia	rs715338	A	G	0.04	0.01	7.9E-12	0.00	0.03	8.9E-01
insomnia	rs7044885	C	G	-0.04	0.01	5.7E-12	0.01	0.03	7.2E-01
insomnia	rs7040224	A	G	0.04	0.01	4.2E-09	0.03	0.03	2.2E-01
insomnia	rs701394	A	G	-0.04	0.01	6.8E-09	0.00	0.03	9.7E-01
insomnia	rs699844	A	G	0.06	0.01	4.1E-08	-0.07	0.05	1.8E-01
insomnia	rs6978112	T	C	0.03	0.01	2.1E-08	0.00	0.03	9.0E-01
insomnia	rs6967168	T	G	-0.04	0.01	1.4E-10	0.00	0.03	8.8E-01
insomnia	rs694786	T	C	-0.04	0.01	2.0E-13	-0.01	0.03	7.5E-01
insomnia	rs6888135	A	C	0.04	0.01	1.2E-10	-0.01	0.03	6.9E-01
insomnia	rs6808140	T	C	0.04	0.01	5.4E-11	-0.02	0.03	4.8E-01
insomnia	rs6756610	C	G	0.04	0.01	1.1E-09	0.00	0.03	9.8E-01
insomnia	rs6734957	T	G	-0.04	0.01	1.8E-09	-0.01	0.03	8.2E-01
insomnia	rs671985	A	G	-0.04	0.01	2.8E-10	0.00	0.03	9.7E-01
insomnia	rs670501	T	C	0.05	0.01	7.4E-13	0.03	0.03	3.2E-01
insomnia	rs6702604	A	G	-0.04	0.01	1.3E-09	0.00	0.03	8.8E-01
insomnia	rs667730	T	C	0.03	0.01	2.3E-08	-0.02	0.03	5.7E-01
insomnia	rs66674044	A	T	-0.06	0.01	2.2E-12	-0.01	0.04	8.2E-01
insomnia	rs6606731	A	T	0.04	0.01	1.5E-08	-0.03	0.03	4.6E-01
insomnia	rs6601080	A	G	0.04	0.01	2.2E-08	-0.02	0.03	5.4E-01
insomnia	rs6597649	T	C	0.03	0.01	3.0E-08	0.01	0.03	6.6E-01
insomnia	rs6589988	A	G	-0.04	0.01	4.7E-09	0.04	0.03	1.3E-01
insomnia	rs6562066	T	C	0.04	0.01	1.4E-10	0.02	0.03	5.5E-01
insomnia	rs6545798	A	T	-0.04	0.01	1.2E-11	-0.02	0.03	5.4E-01
insomnia	rs6510033	A	G	-0.04	0.01	4.7E-08	0.00	0.03	9.2E-01
insomnia	rs647905	T	C	0.03	0.01	2.9E-08	0.01	0.03	7.3E-01
insomnia	rs6465151	T	C	0.06	0.01	1.9E-09	0.03	0.04	4.1E-01
insomnia	rs6457796	T	C	-0.04	0.01	1.1E-08	-0.02	0.03	4.8E-01
insomnia	rs62429521	A	C	0.05	0.01	1.8E-09	0.02	0.04	6.6E-01
insomnia	rs62383308	A	G	-0.06	0.01	4.0E-08	-0.05	0.05	3.3E-01
insomnia	rs623025	T	C	-0.04	0.01	3.2E-08	0.00	0.03	9.3E-01
insomnia	rs62301574	C	G	-0.04	0.01	1.4E-08	-0.03	0.03	3.1E-01
insomnia	rs62264767	A	C	0.06	0.01	1.6E-14	-0.01	0.04	7.0E-01
insomnia	rs62213452	T	G	0.04	0.01	2.4E-08	-0.01	0.03	6.7E-01
insomnia	rs62158170	A	G	0.07	0.01	1.2E-19	0.01	0.03	8.3E-01
insomnia	rs62068188	T	C	0.05	0.01	1.2E-09	0.04	0.04	2.9E-01
insomnia	rs61921611	T	C	-0.04	0.01	7.8E-12	0.05	0.03	9.2E-02
insomnia	rs6119267	C	G	-0.06	0.01	2.3E-20	-0.01	0.03	6.8E-01
insomnia	rs60565673	T	G	-0.04	0.01	1.6E-12	-0.04	0.03	1.4E-01
insomnia	rs6019663	T	C	0.04	0.01	6.5E-10	-0.03	0.03	3.7E-01
insomnia	rs5877	T	C	0.04	0.01	1.2E-08	0.03	0.03	2.8E-01

insomnia	rs566673	T	G	-0.04	0.01	1.2E-10	0.02	0.03	5.5E-01
insomnia	rs56133505	A	G	0.04	0.01	5.6E-12	0.00	0.03	8.7E-01
insomnia	rs56097173	T	C	0.04	0.01	2.7E-10	-0.04	0.03	1.8E-01
insomnia	rs55972276	A	C	0.07	0.01	4.2E-17	0.01	0.04	7.0E-01
insomnia	rs55772859	A	C	0.04	0.01	4.8E-11	0.01	0.03	6.7E-01
insomnia	rs524859	A	G	-0.04	0.01	1.5E-12	0.01	0.03	6.6E-01
insomnia	rs521484	A	G	-0.04	0.01	1.5E-08	0.03	0.03	4.1E-01
insomnia	rs4981170	A	G	-0.05	0.01	7.3E-13	-0.01	0.03	6.7E-01
insomnia	rs492858	T	C	-0.07	0.01	3.5E-09	-0.02	0.05	7.6E-01
insomnia	rs4858708	A	T	-0.03	0.01	1.2E-08	0.00	0.03	9.6E-01
insomnia	rs4788203	A	G	-0.03	0.01	6.3E-09	0.02	0.03	4.9E-01
insomnia	rs4767645	T	G	-0.04	0.01	6.5E-10	0.03	0.03	1.9E-01
insomnia	rs4709655	T	C	-0.05	0.01	3.1E-09	-0.03	0.04	4.2E-01
insomnia	rs4702	A	G	-0.05	0.01	6.8E-16	0.05	0.03	4.6E-02
insomnia	rs4699157	T	C	-0.08	0.02	4.0E-08	-0.03	0.07	6.9E-01
insomnia	rs4664299	T	C	-0.04	0.01	5.0E-09	-0.04	0.03	2.6E-01
insomnia	rs4643373	T	C	0.04	0.01	1.6E-10	0.07	0.03	1.5E-02
insomnia	rs4592425	T	G	0.04	0.01	4.3E-10	0.03	0.03	3.9E-01
insomnia	rs4588900	A	G	0.03	0.01	1.6E-08	0.02	0.03	5.6E-01
insomnia	rs4502882	T	C	-0.04	0.01	8.0E-10	-0.03	0.03	3.1E-01
insomnia	rs429358	T	C	0.05	0.01	2.1E-08	-0.10	0.04	5.3E-03
insomnia	rs4260410	T	C	0.03	0.01	4.9E-08	-0.04	0.03	1.5E-01
insomnia	rs4238755	A	C	-0.04	0.01	2.3E-10	-0.02	0.03	5.5E-01
insomnia	rs4090240	T	C	-0.04	0.01	8.5E-09	-0.01	0.03	7.5E-01
insomnia	rs3902952	T	C	0.05	0.01	2.5E-10	-0.07	0.03	4.9E-02
insomnia	rs3774751	T	G	-0.04	0.01	7.3E-12	0.00	0.03	9.8E-01
insomnia	rs35539975	A	G	0.04	0.01	4.5E-09	0.02	0.03	5.5E-01
insomnia	rs35322724	A	C	0.05	0.01	3.8E-16	-0.03	0.03	3.5E-01
insomnia	rs35110063	A	G	0.04	0.01	8.8E-11	0.04	0.03	1.4E-01
insomnia	rs34967082	A	G	0.04	0.01	4.3E-09	0.03	0.03	2.9E-01
insomnia	rs34490907	C	G	0.05	0.01	1.8E-08	0.01	0.04	8.2E-01
insomnia	rs34214423	A	C	0.04	0.01	3.2E-09	0.04	0.03	2.6E-01
insomnia	rs324017	A	C	0.04	0.01	1.6E-09	0.00	0.03	9.3E-01
insomnia	rs3184470	A	G	-0.04	0.01	9.7E-10	-0.01	0.03	6.3E-01
insomnia	rs314281	T	C	-0.04	0.01	6.0E-13	-0.05	0.03	5.3E-02
insomnia	rs3131638	A	G	-0.04	0.01	7.9E-10	-0.05	0.03	1.2E-01
insomnia	rs2867690	T	C	0.04	0.01	3.7E-08	-0.05	0.03	1.8E-01
insomnia	rs28611339	T	G	0.06	0.01	8.5E-11	0.05	0.04	1.9E-01
insomnia	rs28582096	A	G	-0.05	0.01	1.7E-13	-0.03	0.03	3.2E-01
insomnia	rs28552587	A	G	0.03	0.01	3.3E-08	-0.01	0.03	8.4E-01

insomnia	rs2838787	A	G	-0.04	0.01	7.7E-09	-0.01	0.03	6.2E-01
insomnia	rs2792990	C	G	0.05	0.01	1.2E-10	0.00	0.04	9.4E-01
insomnia	rs2737240	A	G	0.04	0.01	3.4E-08	0.01	0.03	8.0E-01
insomnia	rs2598293	T	C	0.04	0.01	2.5E-09	0.04	0.03	1.1E-01
insomnia	rs2431108	T	C	-0.05	0.01	7.8E-17	0.05	0.03	1.0E-01
insomnia	rs2389631	A	C	-0.04	0.01	2.0E-10	-0.02	0.03	4.5E-01
insomnia	rs2388840	A	G	-0.04	0.01	1.4E-09	-0.04	0.03	1.4E-01
insomnia	rs2364921	T	C	-0.03	0.01	2.1E-08	0.00	0.03	9.3E-01
insomnia	rs2286729	A	G	0.07	0.01	5.4E-11	0.04	0.05	4.2E-01
insomnia	rs224029	T	C	-0.04	0.01	2.5E-10	0.05	0.03	6.9E-02
insomnia	rs2221119	C	G	0.04	0.01	2.0E-09	0.04	0.03	1.8E-01
insomnia	rs2216427	C	G	0.04	0.01	1.6E-08	-0.03	0.03	2.4E-01
insomnia	rs214934	A	T	-0.04	0.01	3.2E-09	-0.04	0.03	1.2E-01
insomnia	rs2089358	T	C	-0.04	0.01	2.7E-10	-0.05	0.03	9.8E-02
insomnia	rs2030672	C	G	0.03	0.01	1.1E-08	0.00	0.03	9.4E-01
insomnia	rs1937447	C	G	-0.04	0.01	2.1E-08	-0.02	0.03	4.7E-01
insomnia	rs1927902	T	C	0.05	0.01	1.1E-14	-0.01	0.03	8.6E-01
insomnia	rs190073	A	G	-0.03	0.01	2.9E-08	-0.05	0.03	8.3E-02
insomnia	rs1861412	A	G	0.04	0.01	1.7E-10	0.04	0.03	1.1E-01
insomnia	rs176644	T	G	0.04	0.01	9.5E-09	0.01	0.03	6.6E-01
insomnia	rs17643634	T	C	-0.06	0.01	1.3E-13	0.02	0.04	6.8E-01
insomnia	rs17520265	A	G	-0.09	0.02	2.9E-08	0.11	0.07	1.3E-01
insomnia	rs17367725	T	C	-0.04	0.01	9.3E-09	-0.02	0.03	4.6E-01
insomnia	rs1731951	A	T	-0.03	0.01	1.4E-08	0.02	0.03	4.8E-01
insomnia	rs17223714	A	G	0.05	0.01	2.4E-10	-0.04	0.03	2.6E-01
insomnia	rs17083297	A	C	-0.04	0.01	1.6E-08	0.00	0.03	9.8E-01
insomnia	rs17025198	A	G	0.04	0.01	2.2E-08	-0.01	0.03	8.6E-01
insomnia	rs17005118	A	G	0.04	0.01	6.1E-10	0.00	0.03	9.0E-01
insomnia	rs16990210	T	C	-0.05	0.01	2.0E-08	-0.01	0.04	8.9E-01
insomnia	rs16903122	T	C	0.06	0.01	9.0E-16	-0.01	0.03	8.7E-01
insomnia	rs1620977	A	G	0.05	0.01	2.3E-14	-0.02	0.03	5.4E-01
insomnia	rs1580173	A	G	0.03	0.01	2.3E-08	0.01	0.03	7.9E-01
insomnia	rs1567084	A	G	0.03	0.01	2.1E-08	0.03	0.03	2.4E-01
insomnia	rs1536053	T	C	-0.04	0.01	6.0E-09	0.00	0.03	9.2E-01
insomnia	rs1530938	A	G	0.04	0.01	8.8E-10	0.04	0.03	1.1E-01
insomnia	rs152555	A	G	-0.05	0.01	4.8E-10	0.02	0.04	5.9E-01
insomnia	rs1519102	C	G	-0.04	0.01	1.9E-08	-0.03	0.03	2.7E-01
insomnia	rs138678612	A	G	-0.12	0.02	1.4E-08	0.07	0.08	4.1E-01
insomnia	rs13138995	A	G	0.03	0.01	2.0E-08	0.02	0.03	5.7E-01
insomnia	rs13135092	A	G	-0.09	0.01	2.5E-16	0.00	0.05	9.7E-01

insomnia	rs13010288	T	G	-0.06	0.01	9.3E-12	-0.03	0.04	5.0E-01
insomnia	rs12991815	C	G	0.04	0.01	3.0E-11	-0.04	0.03	1.5E-01
insomnia	rs12983032	A	G	-0.04	0.01	1.1E-11	0.03	0.03	2.2E-01
insomnia	rs12924275	T	C	0.04	0.01	1.9E-08	-0.01	0.03	8.5E-01
insomnia	rs12917449	A	C	-0.04	0.01	3.0E-08	0.01	0.03	8.1E-01
insomnia	rs12912299	T	C	-0.04	0.01	4.4E-13	0.02	0.03	5.0E-01
insomnia	rs1289939	T	C	-0.04	0.01	6.0E-09	-0.02	0.03	5.2E-01
insomnia	rs12790660	T	C	-0.04	0.01	4.5E-10	0.04	0.03	2.0E-01
insomnia	rs12666306	A	G	0.04	0.01	2.2E-12	0.02	0.03	4.5E-01
insomnia	rs12605642	T	G	0.04	0.01	2.1E-09	-0.03	0.03	2.4E-01
insomnia	rs12520974	T	C	-0.04	0.01	1.7E-09	0.01	0.03	8.3E-01
insomnia	rs12454003	C	G	-0.03	0.01	4.9E-09	-0.01	0.03	7.6E-01
insomnia	rs12310246	A	G	0.04	0.01	4.7E-11	-0.02	0.03	5.4E-01
insomnia	rs12251016	A	T	-0.04	0.01	3.9E-10	0.01	0.03	6.6E-01
insomnia	rs12187443	T	C	0.04	0.01	1.6E-10	0.01	0.03	6.3E-01
insomnia	rs12030482	A	T	0.04	0.01	8.2E-09	-0.02	0.03	5.4E-01
insomnia	rs118166957	T	C	0.07	0.01	2.0E-16	0.02	0.04	6.4E-01
insomnia	rs11803128	A	G	-0.04	0.01	6.9E-11	0.00	0.03	9.8E-01
insomnia	rs11756035	C	G	0.05	0.01	1.3E-08	-0.01	0.04	8.3E-01
insomnia	rs11722569	T	C	0.03	0.01	2.9E-08	0.00	0.03	9.1E-01
insomnia	rs117152417	A	G	-0.15	0.03	2.8E-08	0.15	0.11	1.8E-01
insomnia	rs11679943	A	G	0.04	0.01	3.2E-09	0.00	0.03	9.9E-01
insomnia	rs1167132	T	C	0.04	0.01	8.7E-09	0.02	0.03	4.6E-01
insomnia	rs11650304	C	G	0.07	0.01	1.2E-08	0.01	0.05	8.8E-01
insomnia	rs116466468	T	C	0.04	0.01	2.1E-10	0.00	0.03	9.6E-01
insomnia	rs11605348	A	G	-0.04	0.01	7.0E-13	0.00	0.03	9.6E-01
insomnia	rs11588755	A	G	-0.03	0.01	5.1E-09	-0.02	0.03	3.6E-01
insomnia	rs1147852	A	G	0.04	0.01	9.9E-10	-0.01	0.03	7.1E-01
insomnia	rs113851554	T	G	0.21	0.01	1.6E-51	0.03	0.06	6.6E-01
insomnia	rs11149313	A	G	0.04	0.01	2.4E-09	0.00	0.03	9.2E-01
insomnia	rs11119409	T	C	-0.03	0.01	1.2E-08	0.00	0.03	8.7E-01
insomnia	rs11090039	A	G	0.04	0.01	1.8E-09	-0.03	0.03	3.3E-01
insomnia	rs11001276	A	T	-0.04	0.01	2.5E-08	-0.05	0.03	7.0E-02
insomnia	rs10955647	T	G	0.03	0.01	1.8E-08	0.02	0.03	4.1E-01
insomnia	rs10947987	T	C	-0.03	0.01	4.1E-08	-0.04	0.03	1.4E-01
insomnia	rs10947690	A	G	-0.05	0.01	4.0E-12	0.00	0.03	9.5E-01
insomnia	rs10947428	T	C	-0.07	0.01	9.1E-21	-0.02	0.03	4.8E-01
insomnia	rs10944696	A	G	-0.04	0.01	8.0E-09	0.00	0.03	8.8E-01
insomnia	rs10928256	T	C	0.03	0.01	1.6E-08	0.06	0.03	3.0E-02
insomnia	rs10898940	A	C	0.03	0.01	8.1E-09	0.00	0.03	9.2E-01

insomnia	rs10865954	T	C	0.04	0.01	1.9E-11	0.04	0.03	1.2E-01
insomnia	rs10825503	T	G	0.03	0.01	1.4E-08	0.01	0.03	7.7E-01
insomnia	rs10800992	T	C	0.04	0.01	3.8E-12	0.02	0.03	4.7E-01
insomnia	rs10761240	A	G	-0.04	0.01	2.1E-12	0.01	0.03	8.2E-01
insomnia	rs10758593	A	G	-0.04	0.01	4.9E-09	-0.08	0.03	3.6E-03
insomnia	rs10756571	T	C	0.04	0.01	1.8E-08	-0.03	0.03	2.9E-01
insomnia	rs1064939	A	T	0.13	0.02	2.2E-10	0.05	0.09	6.0E-01
insomnia	rs10502966	A	G	-0.04	0.01	8.5E-11	-0.02	0.03	4.9E-01
insomnia	rs1038093	T	C	0.04	0.01	2.5E-10	0.07	0.03	1.6E-02
insomnia	rs1031654	A	C	-0.05	0.01	3.9E-12	-0.04	0.03	2.4E-01
insomnia	rs1015438	A	G	0.06	0.01	2.5E-14	-0.01	0.03	8.8E-01
sleep duration	rs269054	A	T	0.67	0.13	1.5E-07	-0.06	0.03	3.5E-02
sleep duration	rs61796569	T	C	0.83	0.14	4.5E-09	-0.03	0.03	3.9E-01
sleep duration	rs12567114	A	G	0.85	0.14	2.1E-09	-0.05	0.03	1.0E-01
sleep duration	rs11190970	G	A	0.86	0.16	6.5E-08	-0.01	0.03	6.7E-01
sleep duration	rs7915425	T	C	1.01	0.16	8.2E-10	0.05	0.04	1.6E-01
sleep duration	rs12246842	A	G	0.73	0.13	7.2E-09	0.02	0.03	4.3E-01
sleep duration	rs10761674	C	T	0.70	0.13	2.5E-08	-0.03	0.03	3.5E-01
sleep duration	rs1939455	G	T	1.05	0.20	1.7E-07	0.01	0.04	8.3E-01
sleep duration	rs7115226	A	C	1.59	0.26	1.7E-09	-0.03	0.05	5.0E-01
sleep duration	rs1263056	A	G	0.77	0.13	1.3E-09	0.01	0.03	7.9E-01
sleep duration	rs7951019	G	T	2.21	0.39	1.2E-08	-0.02	0.08	7.5E-01
sleep duration	rs1057703	G	T	0.98	0.19	9.3E-08	0.03	0.04	3.7E-01
sleep duration	rs1517572	C	A	0.81	0.13	1.7E-10	0.00	0.03	9.4E-01
sleep duration	rs4592416	G	A	0.80	0.13	2.3E-10	0.03	0.03	2.7E-01
sleep duration	rs11602180	C	T	0.92	0.17	5.8E-08	-0.02	0.04	5.0E-01
sleep duration	rs174560	C	T	0.79	0.14	5.8E-09	0.04	0.03	1.6E-01
sleep duration	rs12791153	T	A	1.32	0.23	2.6E-08	-0.04	0.05	4.7E-01
sleep duration	rs1553132	G	A	0.85	0.14	5.1E-09	0.00	0.03	9.1E-01
sleep duration	rs4767550	G	A	0.82	0.13	1.1E-10	-0.04	0.03	1.2E-01
sleep duration	rs34354917	C	A	0.67	0.14	1.8E-06	0.04	0.03	1.9E-01
sleep duration	rs6575005	T	C	0.86	0.14	4.3E-09	0.00	0.03	9.9E-01
sleep duration	rs10483350	G	A	0.86	0.16	6.4E-08	0.00	0.03	9.8E-01
sleep duration	rs61985058	T	C	0.94	0.18	2.1E-07	-0.03	0.04	5.0E-01
sleep duration	rs55658675	C	T	0.72	0.13	4.9E-08	0.01	0.03	8.2E-01
sleep duration	rs11621908	C	T	1.27	0.23	3.4E-08	0.01	0.05	9.1E-01
sleep duration	rs8038326	A	G	0.91	0.14	1.2E-10	0.06	0.03	3.3E-02
sleep duration	rs11643715	G	C	0.66	0.14	5.6E-06	-0.05	0.03	1.1E-01
sleep duration	rs9940646	C	G	1.02	0.14	1.2E-13	0.01	0.03	8.1E-01
sleep duration	rs8050478	G	A	0.95	0.13	3.4E-14	0.01	0.03	8.3E-01

sleep duration	rs3095508	C	A	0.70	0.13	3.4E-08	-0.04	0.03	1.3E-01
sleep duration	rs205024	T	C	0.76	0.13	3.0E-09	0.02	0.03	4.3E-01
sleep duration	rs1991556	G	A	0.99	0.16	1.0E-09	0.00	0.03	8.8E-01
sleep duration	rs9903973	C	T	0.75	0.13	1.2E-08	-0.02	0.03	5.4E-01
sleep duration	rs7503199	C	T	0.81	0.14	6.9E-09	0.05	0.03	7.3E-02
sleep duration	rs12607679	T	C	1.21	0.16	8.3E-15	-0.09	0.03	2.5E-03
sleep duration	rs10421649	A	T	0.91	0.13	5.8E-13	-0.01	0.03	6.3E-01
sleep duration	rs7556815	A	G	2.35	0.15	1.8E-54	-0.01	0.03	7.9E-01
sleep duration	rs12611523	A	G	0.70	0.13	2.6E-08	0.00	0.03	9.9E-01
sleep duration	rs4128364	C	T	0.78	0.13	3.9E-09	-0.02	0.03	4.8E-01
sleep duration	rs4538155	T	C	0.76	0.13	5.8E-09	-0.01	0.03	6.1E-01
sleep duration	rs11885663	T	C	0.86	0.14	3.5E-09	-0.03	0.03	3.6E-01
sleep duration	rs10173260	C	T	0.62	0.13	1.6E-06	0.02	0.03	4.3E-01
sleep duration	rs374153	C	T	0.99	0.17	8.5E-09	0.06	0.04	9.9E-02
sleep duration	rs75539574	C	A	1.55	0.17	3.0E-19	0.03	0.05	5.8E-01
sleep duration	rs72804080	G	A	1.07	0.19	2.9E-08	-0.05	0.04	1.5E-01
sleep duration	rs62120041	T	C	1.38	0.27	3.2E-07	-0.01	0.05	8.4E-01
sleep duration	rs2072727	T	C	0.70	0.13	3.8E-08	-0.02	0.03	4.7E-01
sleep duration	rs7644809	T	C	0.62	0.13	1.0E-06	0.02	0.03	4.2E-01
sleep duration	rs13088093	G	T	0.91	0.13	8.4E-12	0.01	0.03	7.9E-01
sleep duration	rs112230981	A	G	0.99	0.28	5.1E-04	-0.11	0.06	6.3E-02
sleep duration	rs17732997	C	G	0.64	0.13	5.2E-07	0.04	0.03	1.1E-01
sleep duration	rs13109404	T	G	1.72	0.26	9.5E-11	0.02	0.05	7.1E-01
sleep duration	rs2192528	A	G	0.67	0.13	9.9E-08	-0.03	0.03	2.8E-01
sleep duration	rs17427571	A	G	0.73	0.13	7.4E-08	0.01	0.03	8.2E-01
sleep duration	rs35531607	C	T	0.71	0.13	2.2E-08	0.01	0.03	6.3E-01
sleep duration	rs56372231	T	C	0.89	0.13	1.8E-11	-0.03	0.03	3.3E-01
sleep duration	rs180769	T	C	0.68	0.13	1.1E-07	-0.01	0.03	7.3E-01
sleep duration	rs365663	A	G	0.70	0.13	3.2E-08	-0.01	0.03	7.1E-01
sleep duration	rs151014368	A	G	0.76	0.15	4.1E-07	-0.03	0.03	4.3E-01
sleep duration	rs460692	C	T	1.21	0.20	3.2E-09	-0.01	0.04	8.9E-01
sleep duration	rs34556183	A	G	0.99	0.15	2.5E-11	0.00	0.03	9.9E-01
sleep duration	rs80193650	G	A	0.77	0.18	2.3E-05	-0.01	0.04	7.6E-01
sleep duration	rs113113059	T	C	0.83	0.16	8.5E-08	-0.06	0.03	4.9E-02
sleep duration	rs9382445	T	C	0.85	0.13	6.3E-11	0.08	0.03	6.4E-03
sleep duration	rs2231265	G	A	0.93	0.15	4.3E-10	0.00	0.03	8.9E-01
sleep duration	rs9345234	C	A	0.71	0.13	2.1E-08	-0.05	0.03	5.6E-02
sleep duration	rs2079070	C	G	1.05	0.15	7.5E-12	-0.06	0.03	4.7E-02
sleep duration	rs7806045	T	C	0.89	0.14	1.1E-09	-0.02	0.03	4.7E-01
sleep duration	rs34731055	T	C	1.04	0.16	1.1E-10	-0.04	0.04	2.3E-01

sleep duration	rs73219758	G	A	0.91	0.14	4.6E-11	-0.04	0.03	1.6E-01
sleep duration	rs330088	C	T	-0.67	0.13	1.1E-07	0.00	0.03	9.0E-01
sleep duration	rs1776776	T	C	1.10	0.20	2.7E-08	-0.06	0.04	1.1E-01
sleep duration	rs10973207	T	G	1.05	0.17	1.3E-09	0.00	0.04	9.2E-01
getting up	rs301806	C	T	-0.01	0.00	4.7E-08	-0.11	0.03	8.2E-05
getting up	rs77576965	T	C	0.01	0.00	1.9E-08	0.02	0.03	5.4E-01
getting up	rs12752290	C	T	0.01	0.00	1.0E-12	-0.01	0.03	8.2E-01
getting up	rs113240734	A	G	0.02	0.00	2.2E-16	-0.05	0.04	1.7E-01
getting up	rs75650221	T	C	0.04	0.00	3.9E-18	-0.08	0.07	2.7E-01
getting up	rs4652514	C	T	-0.01	0.00	9.9E-09	0.00	0.03	9.3E-01
getting up	rs12736689	C	T	0.05	0.01	1.7E-24	-0.02	0.08	8.5E-01
getting up	rs76048411	T	C	0.01	0.00	8.5E-10	0.02	0.03	4.2E-01
getting up	rs2053457	C	T	-0.02	0.00	1.3E-15	-0.03	0.03	2.6E-01
getting up	rs10180284	T	C	0.01	0.00	4.9E-08	-0.03	0.03	2.2E-01
getting up	rs4671328	T	G	0.01	0.00	1.1E-09	-0.05	0.03	9.5E-02
getting up	rs10175975	T	C	0.01	0.00	3.3E-09	-0.07	0.04	4.7E-02
getting up	rs13393656	A	C	0.01	0.00	2.1E-08	0.03	0.03	2.9E-01
getting up	rs406952	C	T	0.01	0.00	1.2E-08	0.01	0.03	8.4E-01
getting up	rs4853283	G	A	-0.01	0.00	1.8E-16	-0.05	0.03	7.5E-02
getting up	rs1606803	T	C	0.01	0.00	3.2E-10	0.02	0.03	4.7E-01
getting up	rs116298301	T	C	-0.02	0.00	2.4E-11	0.04	0.08	6.0E-01
getting up	rs4483990	C	A	-0.02	0.00	6.4E-13	0.03	0.04	4.1E-01
getting up	rs13116306	T	C	-0.01	0.00	1.8E-08	-0.02	0.03	3.7E-01
getting up	rs10470887	G	A	-0.01	0.00	4.0E-08	0.01	0.03	8.2E-01
getting up	rs9995419	A	G	0.01	0.00	1.5E-08	0.01	0.03	8.1E-01
getting up	rs79751662	C	G	-0.02	0.00	2.3E-08	-0.01	0.04	8.7E-01
getting up	rs1459192	T	C	-0.01	0.00	2.5E-08	-0.03	0.03	3.3E-01
getting up	rs12515274	A	G	-0.01	0.00	5.4E-10	0.00	0.03	9.1E-01
getting up	rs4958316	A	C	0.02	0.00	3.7E-15	0.09	0.03	2.6E-03
getting up	rs553108	A	G	0.01	0.00	3.1E-08	-0.04	0.03	1.5E-01
getting up	rs2653349	A	G	0.02	0.00	8.5E-29	-0.03	0.03	3.0E-01
getting up	rs9399613	T	C	-0.01	0.00	1.3E-08	-0.01	0.03	8.1E-01
getting up	rs3735478	T	G	-0.01	0.00	1.6E-08	0.05	0.03	1.0E-01
getting up	rs2944822	T	C	0.01	0.00	4.4E-09	-0.04	0.03	1.3E-01
getting up	rs16917522	C	T	0.01	0.00	1.1E-09	-0.01	0.03	7.9E-01
getting up	rs72663537	G	T	-0.01	0.00	2.8E-08	0.06	0.04	1.1E-01
getting up	rs77641763	T	C	-0.02	0.00	2.0E-11	0.02	0.04	6.2E-01
getting up	rs4962716	T	C	-0.02	0.00	1.8E-09	0.06	0.04	1.3E-01
getting up	rs11229264	A	G	-0.01	0.00	1.4E-10	-0.02	0.03	4.9E-01
getting up	rs7297799	T	C	-0.01	0.00	1.2E-12	-0.03	0.03	2.1E-01

getting up	rs2193749	T	C	-0.01	0.00	1.8E-08	-0.09	0.03	7.3E-04
getting up	rs6581138	A	G	0.01	0.00	2.3E-10	0.01	0.03	6.4E-01
getting up	rs1017168	A	C	-0.01	0.00	2.6E-08	-0.01	0.03	6.1E-01
getting up	rs74643199	T	A	-0.01	0.00	1.2E-08	0.03	0.04	4.4E-01
getting up	rs4884166	A	G	-0.01	0.00	1.2E-08	0.05	0.03	1.3E-01
getting up	rs7332608	G	A	-0.03	0.00	4.6E-11	-0.03	0.07	6.9E-01
getting up	rs6575012	A	G	-0.01	0.00	2.6E-08	0.03	0.03	2.7E-01
getting up	rs3935182	G	C	0.01	0.00	6.8E-12	-0.03	0.03	3.3E-01
getting up	rs1420607	A	G	0.01	0.00	1.6E-11	-0.02	0.03	5.1E-01
getting up	rs11642015	T	C	0.01	0.00	6.2E-11	-0.02	0.03	4.2E-01
getting up	rs1949072	A	G	0.01	0.00	2.5E-08	-0.02	0.03	4.9E-01
getting up	rs17822102	G	A	0.01	0.00	9.4E-10	-0.01	0.03	8.0E-01
getting up	rs11643192	A	C	-0.01	0.00	1.3E-09	0.02	0.03	5.4E-01
getting up	rs4790352	G	A	-0.02	0.00	8.8E-09	0.02	0.05	6.4E-01
getting up	rs3760185	T	C	-0.01	0.00	4.5E-08	0.01	0.03	6.4E-01
getting up	rs7222039	T	C	0.01	0.00	3.4E-08	-0.03	0.03	2.4E-01
getting up	rs12150229	G	A	0.01	0.00	3.5E-09	0.00	0.03	9.6E-01
getting up	rs77556405	A	G	0.02	0.00	1.6E-13	0.00	0.04	9.6E-01
getting up	rs12601968	T	G	-0.01	0.00	1.2E-09	0.00	0.03	9.6E-01
getting up	rs4395148	T	A	-0.01	0.00	2.4E-08	-0.04	0.03	2.1E-01
getting up	rs8182491	T	C	-0.02	0.00	1.2E-09	-0.05	0.05	3.0E-01
getting up	rs3746601	C	A	0.01	0.00	2.9E-08	-0.04	0.03	1.3E-01
getting up	rs11697690	C	T	0.01	0.00	2.3E-08	0.02	0.03	3.6E-01
getting up	rs74555583	A	G	-0.02	0.00	3.0E-08	-0.07	0.05	1.6E-01
morningness	rs12065331	T	C	-0.02	0.00	1.5E-10	0.03	0.03	3.7E-01
morningness	rs17448682	T	C	0.02	0.00	4.0E-13	0.01	0.03	7.2E-01
morningness	rs7543480	T	C	0.02	0.00	6.9E-13	-0.02	0.03	4.0E-01
morningness	rs12140153	T	G	-0.03	0.00	4.6E-12	-0.02	0.05	7.3E-01
morningness	rs11208844	A	G	-0.02	0.00	2.8E-08	0.07	0.04	5.6E-02
morningness	rs11162296	C	G	0.04	0.00	1.5E-34	-0.05	0.04	1.7E-01
morningness	rs17416934	T	C	0.01	0.00	5.9E-09	0.03	0.03	3.1E-01
morningness	rs74802342	A	G	-0.02	0.00	2.1E-08	-0.02	0.04	6.0E-01
morningness	rs72720396	A	G	-0.02	0.00	3.3E-18	-0.01	0.03	6.7E-01
morningness	rs7522677	T	C	0.02	0.00	7.9E-09	0.01	0.03	6.5E-01
morningness	rs12139650	T	G	0.02	0.00	2.2E-08	-0.01	0.03	7.6E-01
morningness	rs10494041	C	G	0.02	0.00	1.0E-12	0.01	0.04	8.9E-01
morningness	rs35461065	T	C	-0.01	0.00	8.4E-09	-0.01	0.03	5.8E-01
morningness	rs2794682	T	C	0.02	0.00	3.9E-22	0.05	0.03	5.3E-02
morningness	rs75650221	T	C	0.03	0.01	8.9E-10	-0.08	0.07	2.7E-01
morningness	rs13306728	A	G	0.03	0.00	6.2E-14	0.04	0.05	3.8E-01

morningness	rs509476	T	C	0.10	0.01	7.4E-56	-0.01	0.08	8.6E-01
morningness	rs12746073	T	C	0.01	0.00	1.7E-08	-0.06	0.03	3.5E-02
morningness	rs12025393	A	G	-0.01	0.00	1.8E-08	0.01	0.03	7.2E-01
morningness	rs16839841	T	G	0.02	0.00	2.7E-08	-0.01	0.05	8.8E-01
morningness	rs13011556	C	G	-0.02	0.00	7.7E-11	-0.04	0.03	2.6E-01
morningness	rs2712056	T	C	0.02	0.00	1.6E-10	-0.01	0.03	7.2E-01
morningness	rs848552	C	G	-0.01	0.00	1.1E-09	0.01	0.03	7.7E-01
morningness	rs62135536	T	C	0.04	0.01	1.4E-08	0.02	0.07	8.1E-01
morningness	rs2592199	C	G	0.02	0.00	1.4E-14	0.04	0.03	2.3E-01
morningness	rs10495976	A	T	-0.02	0.00	1.0E-13	0.00	0.03	9.0E-01
morningness	rs1520524	T	C	0.03	0.00	2.7E-12	-0.01	0.04	7.5E-01
morningness	rs10193431	T	C	0.01	0.00	4.4E-09	0.00	0.03	8.7E-01
morningness	rs17049270	T	C	-0.02	0.00	2.0E-08	-0.04	0.05	4.4E-01
morningness	rs10175975	T	C	0.02	0.00	1.9E-10	-0.07	0.04	4.7E-02
morningness	rs4672440	T	G	0.02	0.00	5.1E-13	-0.01	0.03	6.6E-01
morningness	rs113851554	T	G	-0.03	0.01	1.3E-08	0.03	0.06	6.6E-01
morningness	rs2706762	T	C	-0.02	0.00	3.0E-10	0.02	0.04	5.4E-01
morningness	rs7586062	C	G	-0.02	0.00	3.1E-24	-0.01	0.03	6.1E-01
morningness	rs10190053	A	C	-0.01	0.00	6.5E-09	0.03	0.03	3.2E-01
morningness	rs75863239	T	C	-0.02	0.00	3.2E-08	0.01	0.05	7.9E-01
morningness	rs62172117	A	G	-0.02	0.00	1.4E-17	-0.02	0.03	4.6E-01
morningness	rs7579662	A	G	-0.01	0.00	2.2E-08	0.05	0.03	6.9E-02
morningness	rs13004345	T	C	-0.01	0.00	3.8E-08	-0.01	0.03	8.0E-01
morningness	rs11677484	T	G	0.02	0.00	6.2E-10	-0.01	0.03	8.3E-01
morningness	rs4850712	T	G	0.01	0.00	9.4E-09	0.02	0.03	5.7E-01
morningness	rs6716898	A	G	0.02	0.00	2.3E-22	0.03	0.03	2.8E-01
morningness	rs184033703	A	G	0.03	0.01	9.6E-10	0.00	0.06	9.7E-01
morningness	rs35333999	T	C	-0.05	0.01	5.1E-19	0.01	0.06	9.2E-01
morningness	rs77942338	T	C	-0.06	0.01	1.4E-16	-0.04	0.08	6.4E-01
morningness	rs11900963	A	T	0.05	0.00	3.2E-32	-0.02	0.05	6.3E-01
morningness	rs62182135	A	C	-0.01	0.00	7.8E-09	-0.07	0.03	1.1E-02
morningness	rs17786957	C	G	-0.02	0.00	6.1E-10	0.04	0.04	2.8E-01
morningness	rs7428484	A	G	0.01	0.00	2.0E-08	0.02	0.03	4.8E-01
morningness	rs62263597	A	G	0.03	0.00	4.8E-13	-0.01	0.05	9.0E-01
morningness	rs7652260	C	G	0.02	0.00	2.0E-08	0.02	0.04	5.8E-01
morningness	rs67000219	T	C	-0.02	0.00	2.7E-08	0.04	0.04	3.8E-01
morningness	rs9876864	A	T	0.02	0.00	9.2E-14	-0.03	0.03	3.3E-01
morningness	rs55753638	T	C	-0.02	0.00	2.9E-11	-0.02	0.04	6.6E-01
morningness	rs1800828	C	G	0.01	0.00	5.3E-09	0.01	0.03	7.5E-01
morningness	rs6799356	A	C	0.01	0.00	4.1E-08	-0.01	0.03	6.6E-01

morningness	rs2699869	A	C	0.01	0.00	5.1E-09	0.04	0.03	1.5E-01
morningness	rs1109088	A	G	0.01	0.00	2.6E-09	-0.01	0.03	8.5E-01
morningness	rs6769642	A	C	0.02	0.00	2.8E-13	0.03	0.03	2.2E-01
morningness	rs3850174	A	T	-0.01	0.00	9.1E-09	0.02	0.03	5.9E-01
morningness	rs6443788	A	C	-0.02	0.00	1.0E-09	0.01	0.03	7.4E-01
morningness	rs6443810	C	G	0.02	0.00	2.5E-10	0.06	0.03	5.2E-02
morningness	rs6778003	T	G	0.02	0.00	4.9E-12	-0.04	0.03	1.8E-01
morningness	rs7617588	T	C	-0.02	0.00	3.5E-11	0.01	0.04	7.6E-01
morningness	rs9683585	C	G	-0.01	0.00	1.8E-08	-0.01	0.03	7.4E-01
morningness	rs56040212	A	G	-0.01	0.00	2.2E-08	-0.02	0.03	4.6E-01
morningness	rs28634184	T	C	-0.01	0.00	2.3E-08	0.00	0.03	9.2E-01
morningness	rs57180764	A	G	0.02	0.00	3.2E-11	0.09	0.03	7.6E-03
morningness	rs4241964	T	G	-0.02	0.00	3.5E-12	0.00	0.03	9.4E-01
morningness	rs3797051	T	C	0.02	0.00	1.5E-09	0.04	0.03	1.6E-01
morningness	rs12657776	A	G	0.02	0.00	3.5E-14	0.01	0.03	8.6E-01
morningness	rs304137	A	G	0.02	0.00	3.1E-14	-0.07	0.03	1.1E-02
morningness	rs286808	T	C	0.01	0.00	3.9E-09	0.00	0.03	9.5E-01
morningness	rs2910032	T	C	0.02	0.00	6.2E-18	0.01	0.03	6.9E-01
morningness	rs42210	C	G	-0.01	0.00	3.5E-09	0.03	0.03	3.0E-01
morningness	rs335433	T	C	-0.01	0.00	2.3E-09	-0.03	0.03	2.8E-01
morningness	rs9395520	T	C	0.02	0.00	1.6E-18	-0.04	0.03	1.5E-01
morningness	rs9295795	T	C	-0.03	0.01	1.9E-08	0.03	0.06	6.2E-01
morningness	rs486416	A	G	-0.01	0.00	6.1E-09	0.03	0.03	2.8E-01
morningness	rs734597	A	G	0.02	0.00	1.6E-08	0.05	0.03	1.4E-01
morningness	rs2653349	A	G	0.03	0.00	1.0E-32	-0.03	0.03	3.0E-01
morningness	rs2881955	T	C	0.02	0.00	5.6E-11	0.02	0.03	4.4E-01
morningness	rs9375352	A	T	0.01	0.00	2.0E-08	0.01	0.03	6.7E-01
morningness	rs4557564	A	G	0.03	0.00	2.1E-09	-0.02	0.05	7.3E-01
morningness	rs6935086	T	C	0.02	0.00	5.9E-09	0.02	0.04	6.1E-01
morningness	rs9479402	T	C	-0.10	0.01	1.3E-22	0.04	0.13	7.4E-01
morningness	rs9348050	T	C	0.01	0.00	2.0E-09	0.02	0.03	4.7E-01
morningness	rs16873715	A	T	-0.02	0.00	4.4E-08	0.06	0.03	6.6E-02
morningness	rs56382918	T	C	0.02	0.00	1.1E-12	0.02	0.03	4.9E-01
morningness	rs10236197	T	C	0.01	0.00	2.0E-10	-0.02	0.03	5.5E-01
morningness	rs56049037	A	G	-0.02	0.00	2.3E-12	-0.05	0.03	1.2E-01
morningness	rs4245555	T	C	-0.02	0.00	2.0E-15	0.03	0.03	2.0E-01
morningness	rs2138759	A	G	0.01	0.00	1.7E-09	-0.03	0.03	3.4E-01
morningness	rs2922966	A	G	0.03	0.00	1.1E-17	-0.04	0.04	2.5E-01
morningness	rs202157	T	C	-0.02	0.00	1.2E-14	0.00	0.03	9.9E-01
morningness	rs112613078	A	G	-0.03	0.00	1.5E-20	-0.01	0.03	7.9E-01

morningness	rs10262462	A	G	-0.02	0.00	3.2E-12	-0.06	0.03	2.1E-02
morningness	rs6978514	T	C	-0.01	0.00	4.9E-08	-0.01	0.03	8.4E-01
morningness	rs2971970	T	G	0.02	0.00	2.2E-11	0.07	0.03	2.4E-02
morningness	rs35748596	T	G	-0.02	0.00	2.4E-12	0.01	0.03	8.6E-01
morningness	rs34344642	T	G	-0.02	0.00	2.9E-09	0.00	0.05	9.7E-01
morningness	rs12541362	A	T	-0.02	0.00	5.6E-15	0.02	0.03	5.6E-01
morningness	rs1919346	A	G	0.01	0.00	1.3E-08	-0.01	0.03	8.1E-01
morningness	rs11988076	A	G	-0.02	0.00	1.8E-11	0.01	0.04	8.8E-01
morningness	rs6472936	T	C	0.02	0.00	5.2E-12	-0.03	0.03	3.4E-01
morningness	rs1110275	T	C	0.02	0.00	4.1E-08	-0.02	0.05	6.5E-01
morningness	rs34578339	A	T	-0.02	0.00	2.8E-08	-0.01	0.04	7.8E-01
morningness	rs72673588	C	G	-0.02	0.00	1.3E-09	0.03	0.03	3.9E-01
morningness	rs3100052	A	G	0.01	0.00	1.3E-08	-0.02	0.03	4.9E-01
morningness	rs2737245	T	G	0.02	0.00	2.9E-14	0.00	0.03	9.7E-01
morningness	rs1323591	T	C	-0.02	0.00	2.1E-12	-0.01	0.03	7.2E-01
morningness	rs2291589	T	G	0.02	0.00	1.7E-16	0.02	0.03	5.2E-01
morningness	rs77598468	A	C	-0.04	0.01	1.1E-12	-0.10	0.07	1.7E-01
morningness	rs4565536	A	C	-0.01	0.00	5.0E-08	0.05	0.03	9.0E-02
morningness	rs10797119	T	C	-0.01	0.00	2.8E-09	-0.02	0.03	5.6E-01
morningness	rs28365587	A	G	-0.01	0.00	3.6E-09	-0.04	0.03	1.4E-01
morningness	rs10448340	T	G	-0.01	0.00	9.1E-09	0.02	0.03	4.5E-01
morningness	rs28458909	T	C	-0.03	0.00	1.9E-16	0.02	0.04	6.0E-01
morningness	rs1750785	A	G	0.01	0.00	3.5E-09	-0.01	0.03	7.0E-01
morningness	rs9416744	A	C	0.02	0.00	1.5E-12	0.03	0.03	3.7E-01
morningness	rs7910164	A	G	0.01	0.00	4.8E-08	-0.01	0.03	7.1E-01
morningness	rs76518095	T	C	0.02	0.00	1.0E-08	0.02	0.05	6.3E-01
morningness	rs9795439	A	G	0.02	0.00	6.7E-09	0.05	0.03	1.8E-01
morningness	rs925947	T	G	0.02	0.00	9.6E-10	-0.03	0.03	4.3E-01
morningness	rs12799529	T	C	0.02	0.00	5.1E-13	0.05	0.03	1.2E-01
morningness	rs11032362	A	G	0.03	0.00	3.5E-17	-0.03	0.05	5.6E-01
morningness	rs34239319	T	G	0.02	0.00	6.5E-12	-0.14	0.04	1.9E-03
morningness	rs11039308	A	G	0.01	0.00	4.9E-11	0.01	0.03	7.0E-01
morningness	rs3168135	A	G	-0.02	0.00	3.6E-12	-0.03	0.03	3.2E-01
morningness	rs4008953	A	G	-0.02	0.00	3.2E-11	0.00	0.03	9.0E-01
morningness	rs4237555	T	C	0.01	0.00	4.2E-08	0.00	0.03	8.9E-01
morningness	rs4936290	A	C	-0.01	0.00	1.2E-08	-0.02	0.03	4.9E-01
morningness	rs577924	T	C	0.01	0.00	2.1E-08	0.02	0.03	5.4E-01
morningness	rs1174510	A	G	0.01	0.00	4.9E-08	-0.01	0.03	6.5E-01
morningness	rs11611435	T	C	0.01	0.00	4.0E-08	0.00	0.03	9.9E-01
morningness	rs7313852	A	G	-0.03	0.00	1.0E-29	-0.02	0.03	4.0E-01

morningness	rs11183201	T	C	-0.02	0.00	1.6E-14	-0.09	0.03	1.1E-03
morningness	rs671255	A	G	-0.01	0.00	1.4E-08	-0.04	0.03	1.8E-01
morningness	rs7299922	A	G	0.01	0.00	8.6E-09	0.01	0.03	5.9E-01
morningness	rs7138306	A	G	0.01	0.00	8.9E-10	0.03	0.03	2.3E-01
morningness	rs7488974	A	G	0.02	0.00	1.8E-13	-0.03	0.03	2.4E-01
morningness	rs10861694	T	C	0.01	0.00	2.6E-10	0.03	0.03	1.9E-01
morningness	rs4102203	T	C	0.02	0.00	7.8E-12	0.07	0.04	9.5E-02
morningness	rs61963123	T	C	0.02	0.00	2.3E-10	0.03	0.03	3.1E-01
morningness	rs9597250	A	C	-0.02	0.00	2.9E-12	0.04	0.03	2.9E-01
morningness	rs2593487	A	G	-0.01	0.00	2.9E-10	0.00	0.03	9.7E-01
morningness	rs9565309	T	C	0.07	0.01	1.3E-28	0.03	0.07	6.9E-01
morningness	rs7337911	A	G	0.02	0.00	1.0E-09	0.02	0.03	4.7E-01
morningness	rs9521184	T	C	0.01	0.00	8.3E-10	-0.01	0.03	7.3E-01
morningness	rs56376592	A	C	0.02	0.00	1.4E-09	0.05	0.04	1.2E-01
morningness	rs4899502	A	G	-0.02	0.00	1.6E-11	-0.01	0.03	8.1E-01
morningness	rs12432176	A	C	0.01	0.00	1.5E-08	0.00	0.03	9.2E-01
morningness	rs2701524	T	C	0.01	0.00	5.4E-09	0.03	0.03	3.5E-01
morningness	rs59986227	C	G	-0.02	0.00	5.4E-09	-0.07	0.03	2.0E-02
morningness	rs12442008	T	C	0.01	0.00	3.4E-08	-0.02	0.03	5.2E-01
morningness	rs11852820	C	G	-0.01	0.00	5.8E-10	-0.01	0.03	6.4E-01
morningness	rs2304467	C	G	-0.01	0.00	3.9E-08	0.03	0.03	2.9E-01
morningness	rs7196720	T	C	0.01	0.00	4.3E-09	0.03	0.03	2.2E-01
morningness	rs12927162	A	G	0.03	0.00	2.1E-32	-0.04	0.03	1.4E-01
morningness	rs1421085	T	C	-0.02	0.00	1.4E-25	0.02	0.03	4.4E-01
morningness	rs2398144	A	C	-0.02	0.00	2.4E-21	-0.01	0.03	8.0E-01
morningness	rs8044054	T	C	0.02	0.00	3.9E-12	0.01	0.03	6.1E-01
morningness	rs17604349	A	G	-0.02	0.00	1.2E-15	-0.03	0.04	4.6E-01
morningness	rs2518022	T	C	0.03	0.00	1.5E-16	0.09	0.05	7.7E-02
morningness	rs2232839	T	C	-0.03	0.00	2.4E-21	0.03	0.03	3.6E-01
morningness	rs9915731	A	T	-0.01	0.00	2.8E-08	0.01	0.03	7.5E-01
morningness	rs225289	T	C	0.02	0.00	1.2E-08	-0.02	0.04	5.2E-01
morningness	rs72828815	T	C	0.02	0.00	6.1E-09	-0.01	0.03	8.6E-01
morningness	rs117974417	C	G	-0.02	0.00	1.5E-09	0.03	0.04	4.9E-01
morningness	rs6504758	A	G	-0.01	0.00	9.8E-11	-0.02	0.03	4.0E-01
morningness	rs9898091	T	C	0.04	0.01	6.9E-12	-0.12	0.07	6.0E-02
morningness	rs8072058	A	T	-0.02	0.00	1.4E-08	0.01	0.03	7.1E-01
morningness	rs17682747	A	G	0.01	0.00	4.7E-08	-0.05	0.03	1.1E-01
morningness	rs2916142	T	C	0.02	0.00	7.6E-12	-0.02	0.03	5.3E-01
morningness	rs10491171	C	G	0.02	0.00	3.6E-09	-0.01	0.04	7.0E-01
morningness	rs487952	A	G	0.01	0.00	4.4E-09	-0.04	0.03	1.5E-01

morningness	rs974552	A	G	-0.02	0.00	1.4E-15	0.02	0.03	4.8E-01
morningness	rs1013987	T	C	-0.02	0.00	1.4E-11	-0.01	0.03	6.5E-01
morningness	rs4239386	A	T	-0.02	0.00	2.1E-17	-0.04	0.03	1.5E-01
morningness	rs12969848	T	C	0.02	0.00	1.5E-15	-0.02	0.03	4.2E-01
morningness	rs989885	A	G	-0.02	0.00	3.8E-08	0.04	0.06	5.2E-01
morningness	rs9956387	A	T	-0.01	0.00	1.4E-10	0.01	0.03	8.2E-01
morningness	rs17596722	T	C	-0.02	0.00	3.2E-11	-0.02	0.03	6.1E-01
morningness	rs9964420	A	C	-0.02	0.00	4.0E-21	-0.01	0.03	7.5E-01
morningness	rs11152350	A	C	-0.02	0.00	1.1E-12	0.03	0.03	2.9E-01
morningness	rs9958145	A	G	-0.02	0.00	4.3E-08	-0.01	0.03	6.6E-01
morningness	rs10402849	T	C	0.02	0.00	6.7E-09	-0.02	0.03	6.0E-01
morningness	rs3843751	T	C	0.01	0.00	6.3E-09	0.02	0.03	5.8E-01
morningness	rs9636202	A	G	-0.01	0.00	3.5E-08	-0.04	0.03	1.9E-01
morningness	rs12481462	T	C	0.01	0.00	4.4E-08	-0.02	0.03	5.0E-01
morningness	rs78095690	T	C	-0.01	0.00	5.8E-09	0.01	0.03	6.3E-01
morningness	rs6131942	A	G	-0.02	0.00	7.6E-12	-0.01	0.03	7.6E-01
morningness	rs1737893	T	C	-0.01	0.00	1.4E-09	0.03	0.03	3.4E-01
morningness	rs2072727	T	C	0.01	0.00	3.5E-08	-0.02	0.03	4.7E-01
morningness	rs695459	T	C	-0.01	0.00	6.6E-09	0.01	0.03	7.5E-01
morningness	rs28580373	A	G	0.01	0.00	3.3E-08	-0.01	0.03	7.6E-01
morningness	rs11705370	A	T	-0.02	0.00	3.4E-15	0.02	0.03	4.0E-01
morningness	rs4822107	A	G	0.01	0.00	2.2E-08	0.05	0.03	8.5E-02
morningness	rs2294203	A	G	0.01	0.00	3.0E-08	-0.03	0.03	3.6E-01
snoring	rs35562935	A	G	1.06	0.01	1.7E-05	0.08	0.05	9.7E-02
snoring	rs72906130	G	C	1.06	0.01	3.5E-14	0.04	0.04	3.4E-01
snoring	rs9309771	A	G	1.03	0.01	5.7E-09	0.00	0.03	9.2E-01
snoring	rs34811474	A	G	0.96	0.01	4.2E-08	-0.03	0.03	3.4E-01
snoring	rs6855873	T	C	1.03	0.01	2.1E-05	0.05	0.03	1.0E-01
snoring	rs2307111	C	T	0.97	0.01	1.3E-08	0.03	0.03	2.4E-01
snoring	rs10062026	A	G	0.97	0.01	2.1E-05	-0.01	0.03	6.6E-01
snoring	rs947612	G	A	1.03	0.01	9.9E-06	-0.01	0.03	8.2E-01
snoring	rs17060460	G	A	1.03	0.01	1.4E-08	0.02	0.03	4.6E-01
snoring	rs9389081	A	T	0.95	0.01	4.4E-08	0.01	0.05	8.1E-01
snoring	rs2981329	C	T	1.03	0.01	7.7E-09	0.00	0.03	8.8E-01
snoring	rs7007887	T	C	1.04	0.01	9.9E-14	0.00	0.03	9.8E-01
snoring	rs4523230	A	T	0.97	0.01	1.7E-09	0.02	0.03	5.5E-01
snoring	rs1016013	A	G	0.97	0.01	7.2E-10	0.02	0.03	4.2E-01
snoring	rs11256034	T	C	1.04	0.01	4.7E-11	0.06	0.03	6.1E-02
snoring	rs2049045	C	G	0.96	0.01	8.9E-11	-0.02	0.03	6.4E-01
snoring	rs10878269	T	C	1.04	0.01	3.8E-13	-0.05	0.03	9.1E-02

snoring	rs12427782	T	G	0.97	0.01	5.8E-09	-0.01	0.03	6.2E-01
snoring	rs2762049	C	G	1.04	0.01	8.7E-09	0.08	0.03	2.2E-03
snoring	rs592333	G	A	1.04	0.01	1.1E-13	-0.03	0.03	3.5E-01
snoring	rs2664299	C	T	0.97	0.01	4.5E-08	0.02	0.03	5.3E-01
snoring	rs9933881	C	T	1.06	0.01	8.9E-09	0.06	0.05	2.7E-01
snoring	rs732172	T	C	1.03	0.01	1.3E-09	0.01	0.03	7.8E-01
snoring	rs8047587	T	G	1.03	0.01	2.1E-09	-0.01	0.03	7.1E-01
snoring	rs12449843	A	G	0.97	0.01	4.4E-10	0.00	0.03	8.9E-01
snoring	rs1641511	G	A	1.03	0.01	1.4E-08	0.05	0.03	1.0E-01
snoring	rs57222984	G	A	1.04	0.01	3.0E-11	0.01	0.03	8.0E-01
snoring	rs2924251	A	G	1.03	0.01	5.2E-09	0.00	0.03	9.0E-01
snoring	rs180107	A	T	0.97	0.01	3.9E-09	-0.01	0.03	8.0E-01
snoring	rs4987719	T	C	1.08	0.01	3.6E-08	-0.07	0.08	3.3E-01
snoring	rs10415992	G	C	0.95	0.01	8.1E-09	-0.02	0.05	6.9E-01
snoring	rs34107769	C	T	0.97	0.01	9.9E-09	0.00	0.03	9.2E-01
snoring	rs6099273	T	C	1.03	0.01	1.2E-08	0.02	0.03	5.0E-01

Note: SNP, EA, effect allele; OA, other allele; EAF, effect allele frequence; Se, standard error; getting up, The ease of getting up in the morning.

Table S7. Sensitivity analyses of sleep traits with CAD with diabetes.

Sleep traits	Method	OR	95% CI	*p*-value
Insomnia	Weighted median	1.13	1.00 - 1.27	0.08
	MR-Egger	1.04	0.67 - 1.41	0.84
	MR-PRESSO	1.16	1.07 - 1.25	0.01
Sleep duration	Weighted median	1.00	0.98 - 1.01	0.56
	MR-Egger	0.99	0.96 - 1.02	0.45
	MR-PRESSO	1.00	0.98 - 1.00	0.08
Easy to get up	Weighted median	0.57	-0.39 - 1.53	0.25
	MR-Egger	0.27	-2.24 - 2.78	0.31
	MR-PRESSO	0.85	0.11 - 1.60	0.68
Morningness	Weighted median	0.92	0.55 - 1.28	0.64
	MR-Egger	0.70	-0.06 - 1.46	0.36
	MR-PRESSO	1.05	0.80 - 1.30	0.70
Snoring	Weighted median	1.01	1.00 - 1.02	0.54
	MR-Egger	1.18	0.88 - 1.47	0.28
	MR-PRESSO	1.01	1.00 - 1.02	0.01

Note: OR, odds ratio; CI, confidence interval;MR, Mendelian randomization; MR-PRESSO, MR-pleiotropy residual sum and outlier;Easy to get up, ease of getting up in the morning.

Figure Legend

FigureS1. Sensitivity analysis (A), scatter plot (B), forest plot (C), and funnel plot (D) of the causal effect of insomnia on CAD in patients with diabetes.

FigureS2. Sensitivity analysis (A), scatter plot (B), forest plot (C), and funnel plot (D) of the causal effect of sleep duration on CAD in patients with diabetes.

FigureS3. Sensitivity analysis (A), scatter plot (B), forest plot (C), and funnel plot (D) of the causal effect of getting up on CAD in patients with diabetes.

FigureS4. Sensitivity analysis (A), scatter plot (B), forest plot (C), and funnel plot (D) of the causal effect of morningness on CAD in patients with diabetes.

FigureS5. Sensitivity analysis (A), scatter plot (B), forest plot (C), and funnel plot (D) of the causal effect of snoring on CAD in patients with diabetes

FigureS1. Sensitivity analysis (A), scatter plot (B), forest plot (C), and funnel plot (D) of the causal effect of insomnia on CAD in patients with diabetes.

(A)

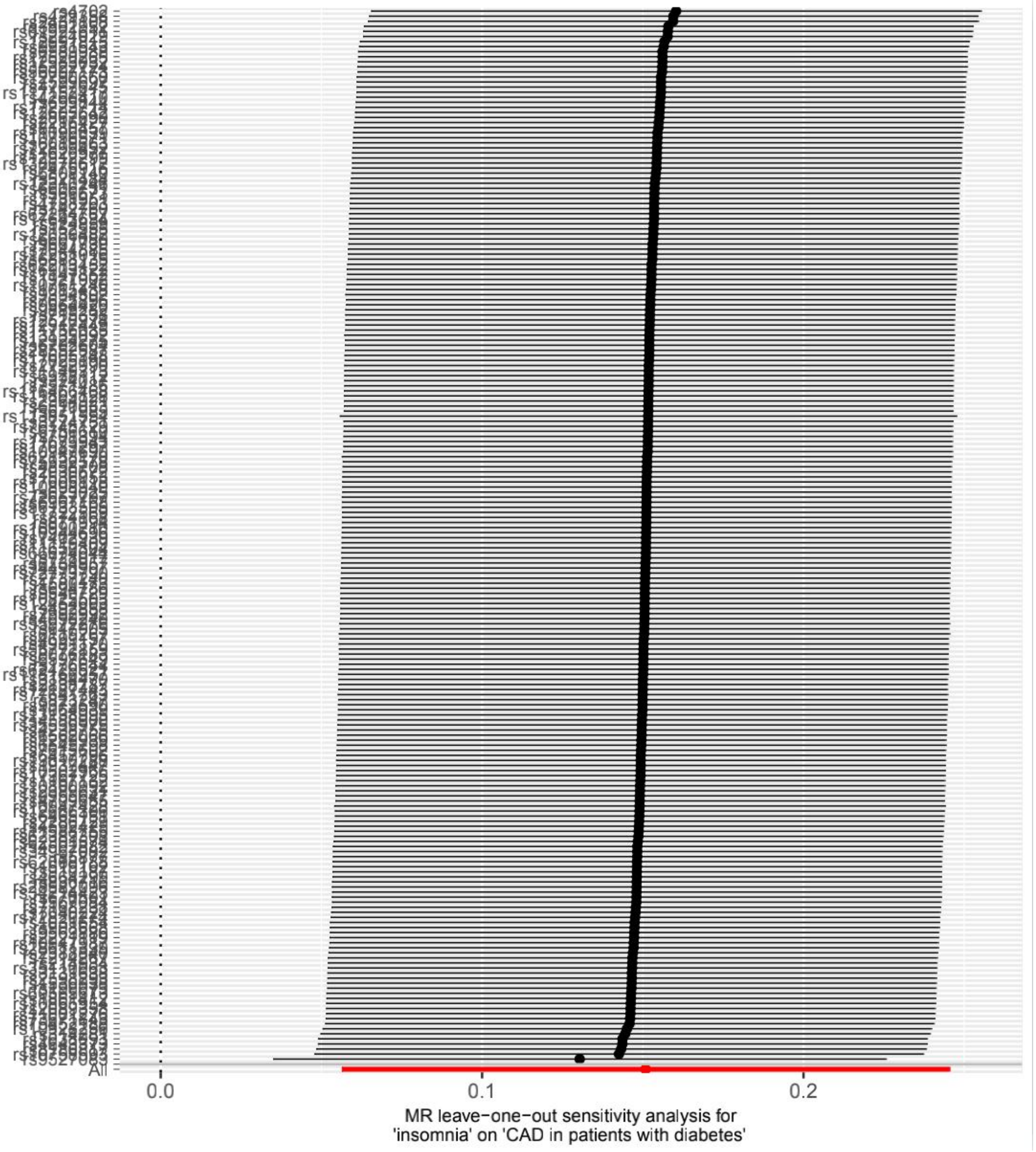

FigureS1.(B)

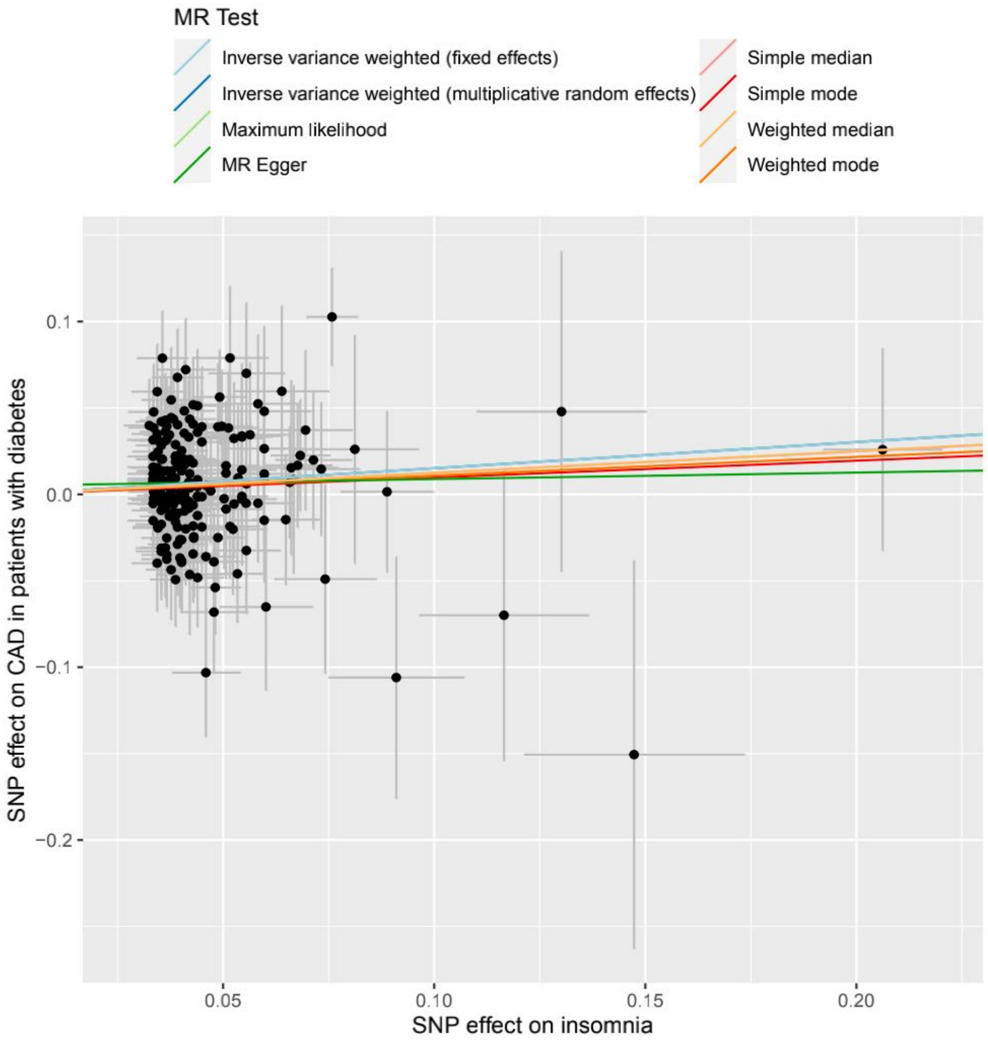
MR Test
Inverse variance weighted (fixed effects)
Inverse variance weighted (multiplicative random effects)
Maximum likelihood
MR Egger
Simple median
Simple mode
Weighted median
Weighted mode
0.1
0.0
−0.1
−0.2
0.05
0.10
0.15
0.20
SNP effect on CAD in patients with diabetes
SNP effect on insomnia

FigureS1.(C)

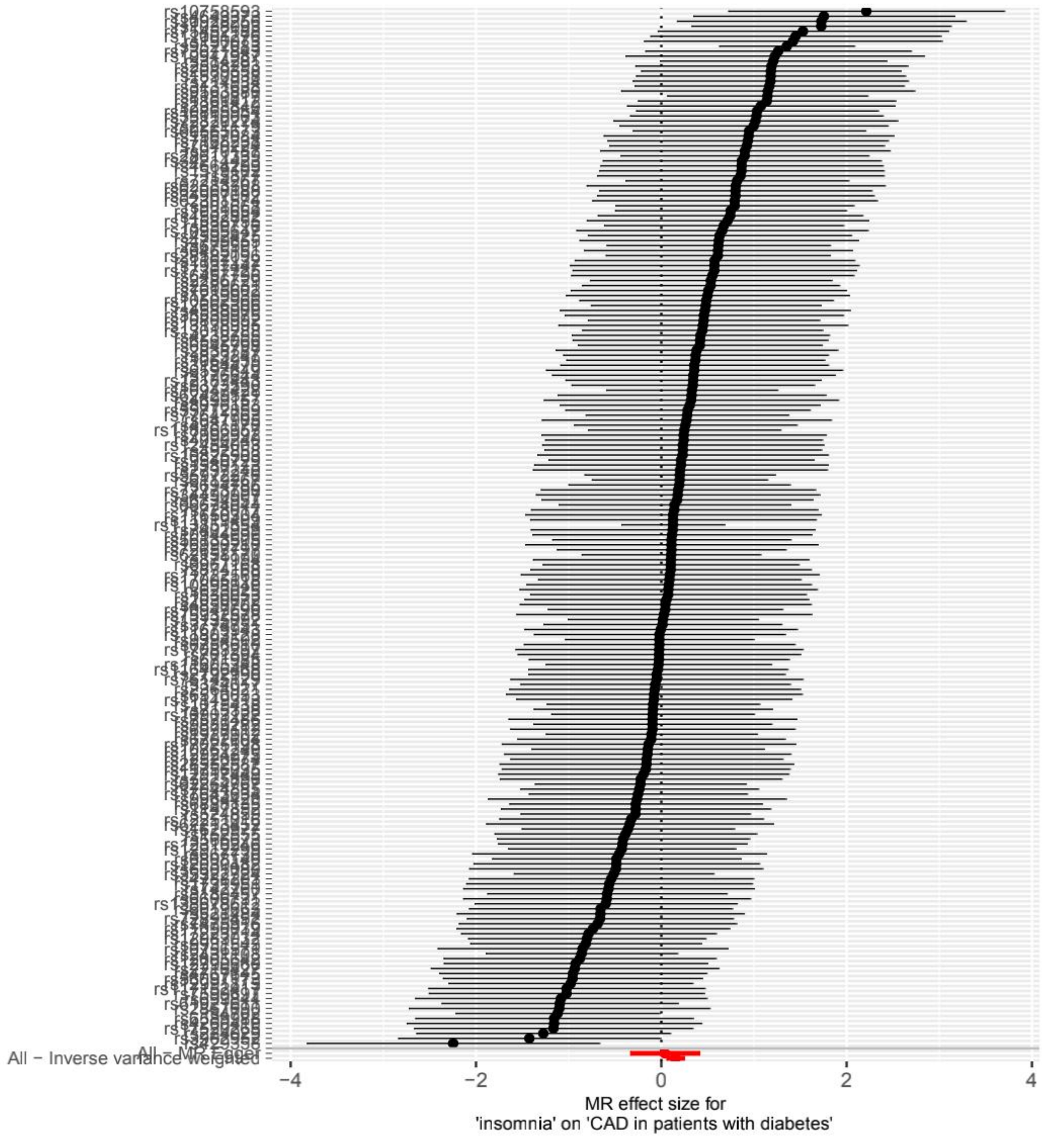
All − MR Egger
All − Inverse variance weighted
−4
−2
0
2
4
MR effect size for
'insomnia' on 'CAD in patients with diabetes'

FigureS1.(D)

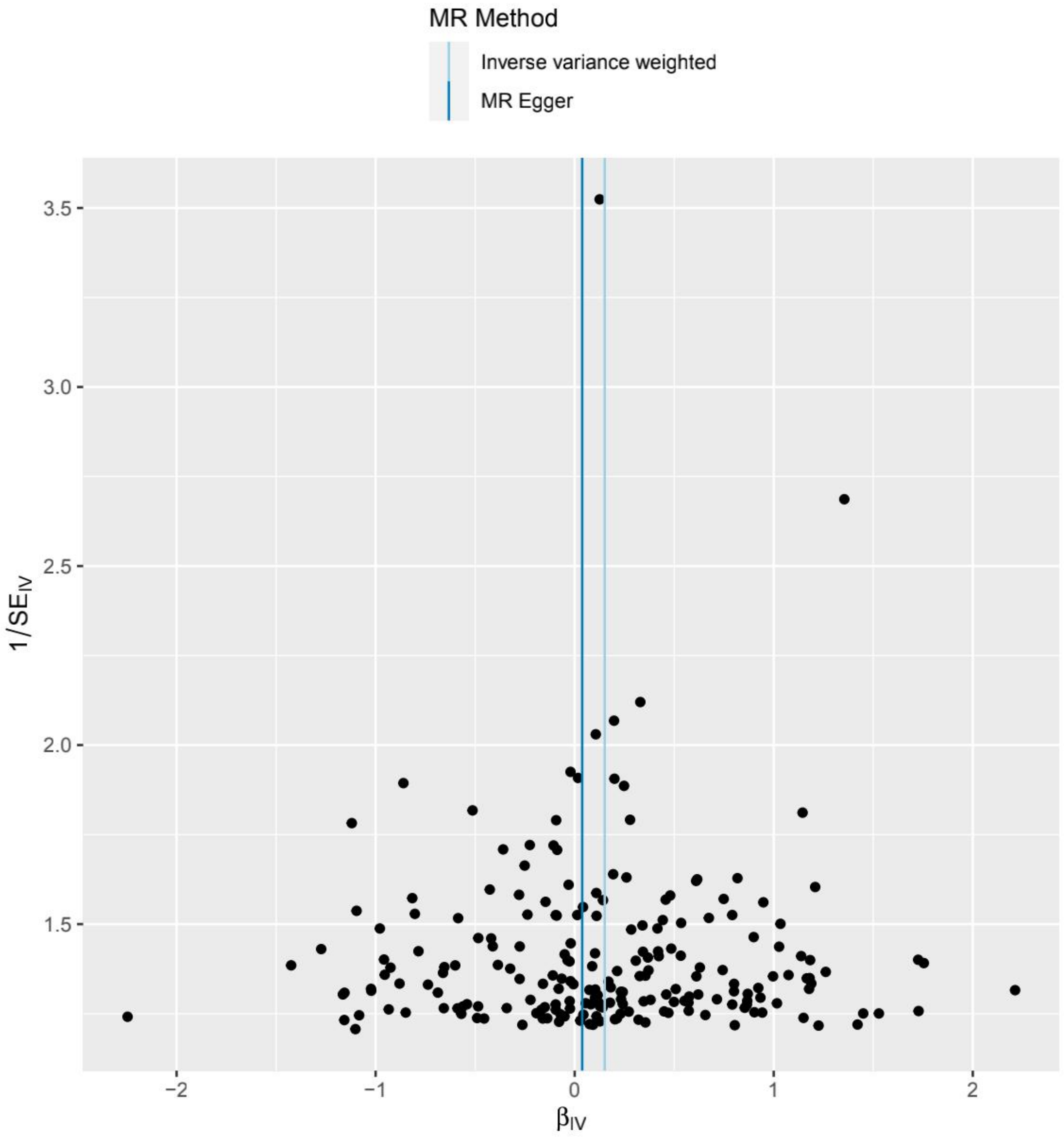

MR Method
Inverse variance weighted
MR Egger
3.5
3.0
2.5
2.0
1.5
1/SE_IV
−2
−1
0
1
2
β_IV

FigureS2. Sensitivity analysis (A), scatter plot (B), forest plot (C), and funnel plot (D) of the causal effect of sleep duration on CAD in patients with diabetes.

(A)

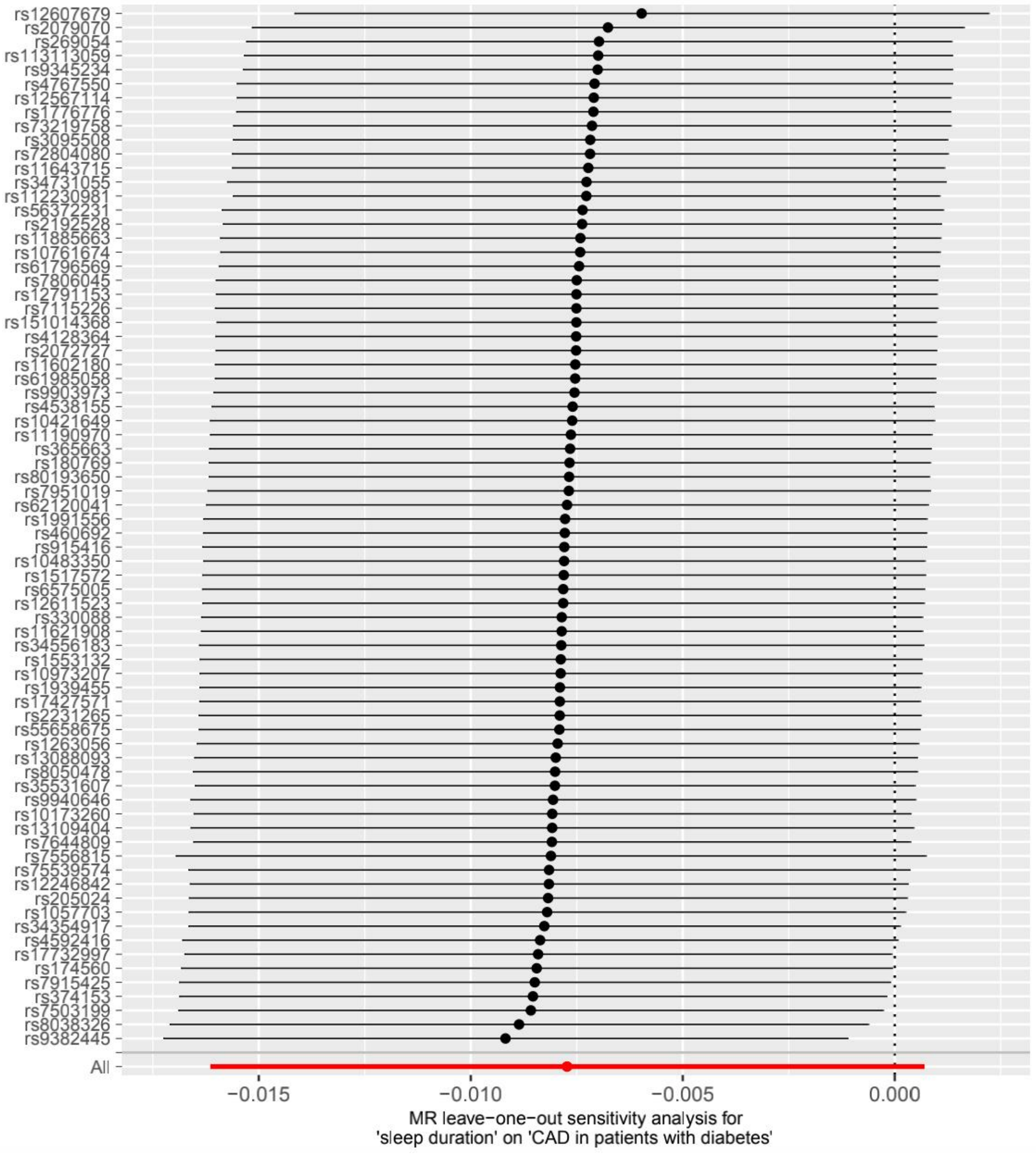

FigureS2.(B)

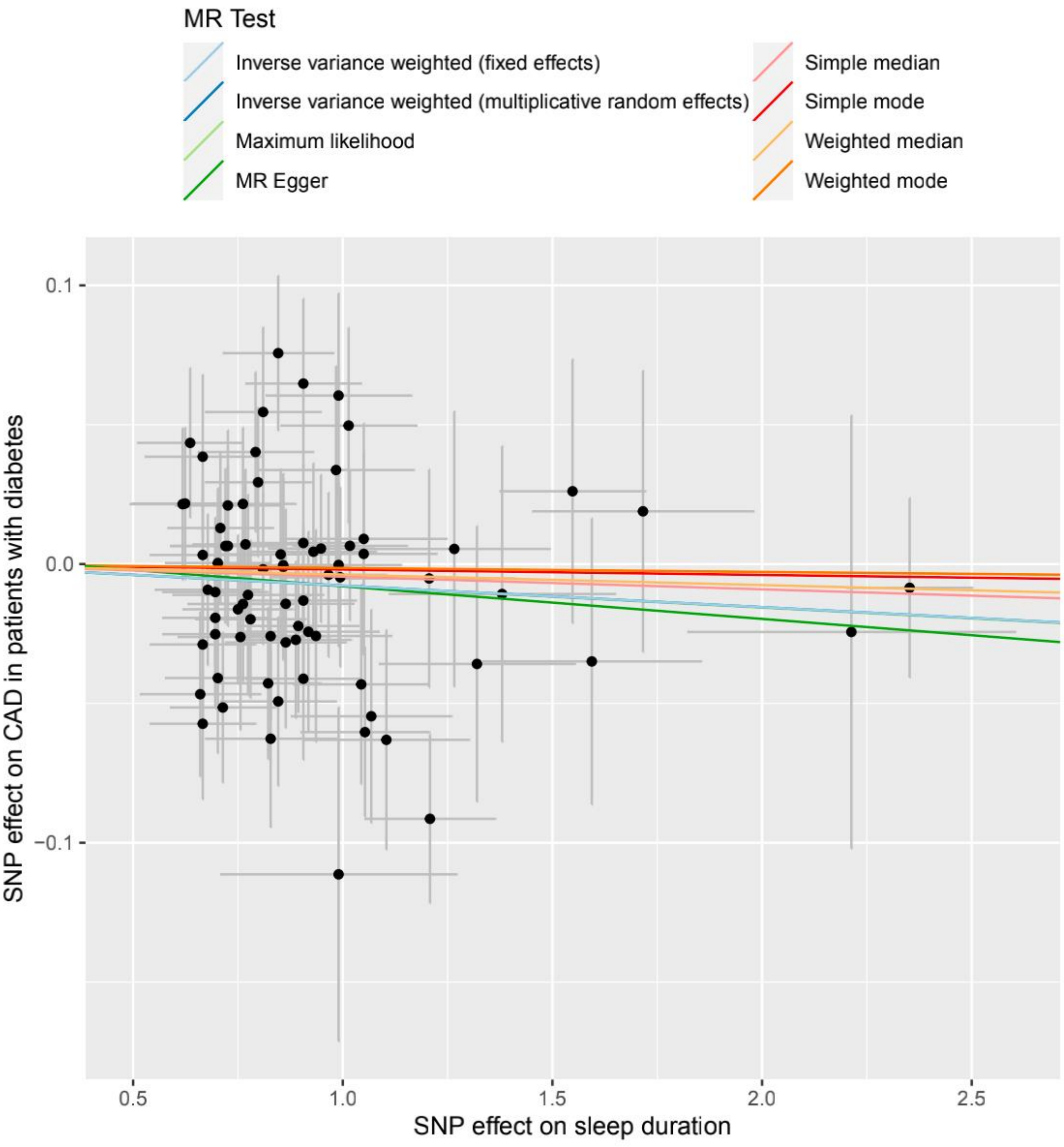

MR Test
Inverse variance weighted (fixed effects)
Inverse variance weighted (multiplicative random effects)
Maximum likelihood
MR Egger
Simple median
Simple mode
Weighted median
Weighted mode
0.1
0.0
−0.1
0.5
1.0
1.5
2.0
2.5
SNP effect on CAD in patients with diabetes
SNP effect on sleep duration

FigureS2.(C)

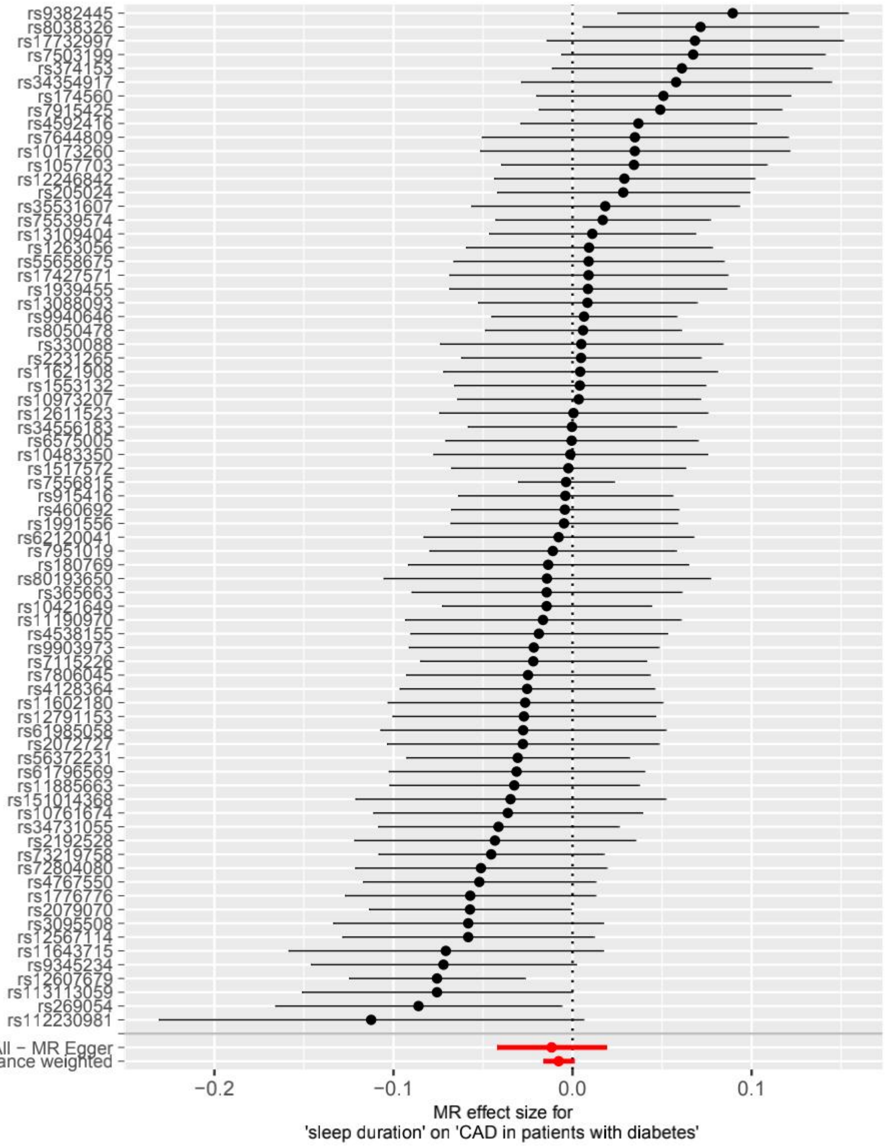

rs9382445
rs8038326
rs17732997
rs7503199
rs374153
rs34354917
rs174560
rs7915425
rs4592416
rs7644809
rs10173260
rs1057703
rs12246842
rs205024
rs35531607
rs75539574
rs13109404
rs1263056
rs55658675
rs17427571
rs1939455
rs13088093
rs9940646
rs8050478
rs330088
rs2231265
rs11621908
rs1553132
rs10973207
rs12611523
rs34556183
rs6575005
rs10483350
rs1517572
rs7556815
rs915416
rs460692
rs1991556
rs62120041
rs7951019
rs180769
rs80193650
rs365663
rs10421649
rs11190970
rs4538155
rs9903973
rs7115226
rs7806045
rs4128364
rs11602180
rs12791153
rs61985058
rs2072727
rs56372231
rs61796569
rs11885663
rs151014368
rs10761674
rs34731055
rs2192528
rs73219758
rs72804080
rs4767550
rs1776776
rs2079070
rs3095508
rs12567114
rs11643715
rs9345234
rs12607679
rs113113059
rs269054
rs112230981
All − MR Egger
All − Inverse variance weighted
−0.2
−0.1
0.0
0.1
MR effect size for
'sleep duration' on 'CAD in patients with diabetes'

FigureS2.(D)

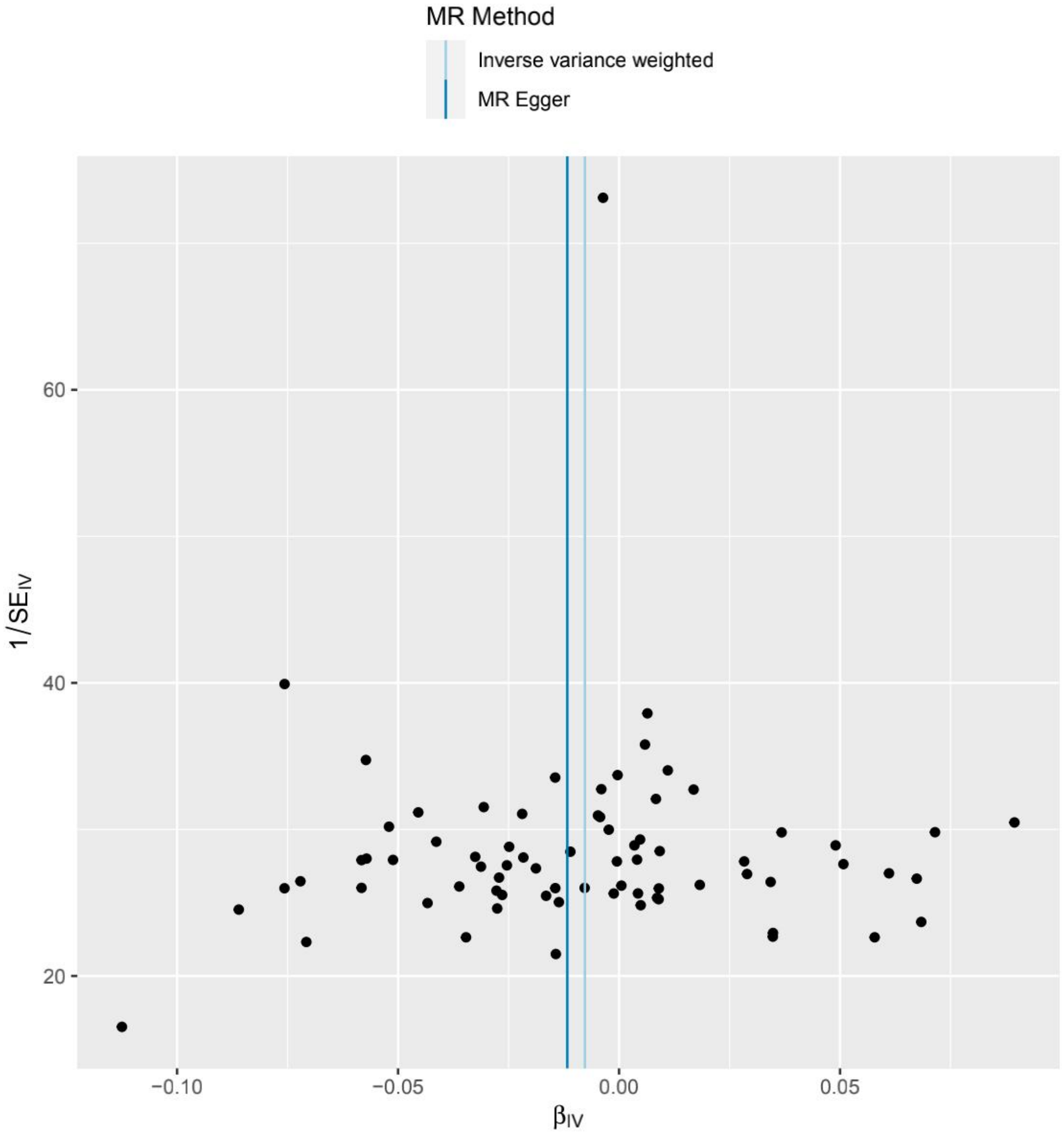
MR Method
Inverse variance weighted
MR Egger
60
40
20
1/SE$_{IV}$
−0.10
−0.05
0.00
0.05
β$_{IV}$

FigureS3. Sensitivity analysis (A), scatter plot (B), forest plot (C), and funnel plot (D) of the causal effect of getting up on CAD in patients with diabetes.

(A)

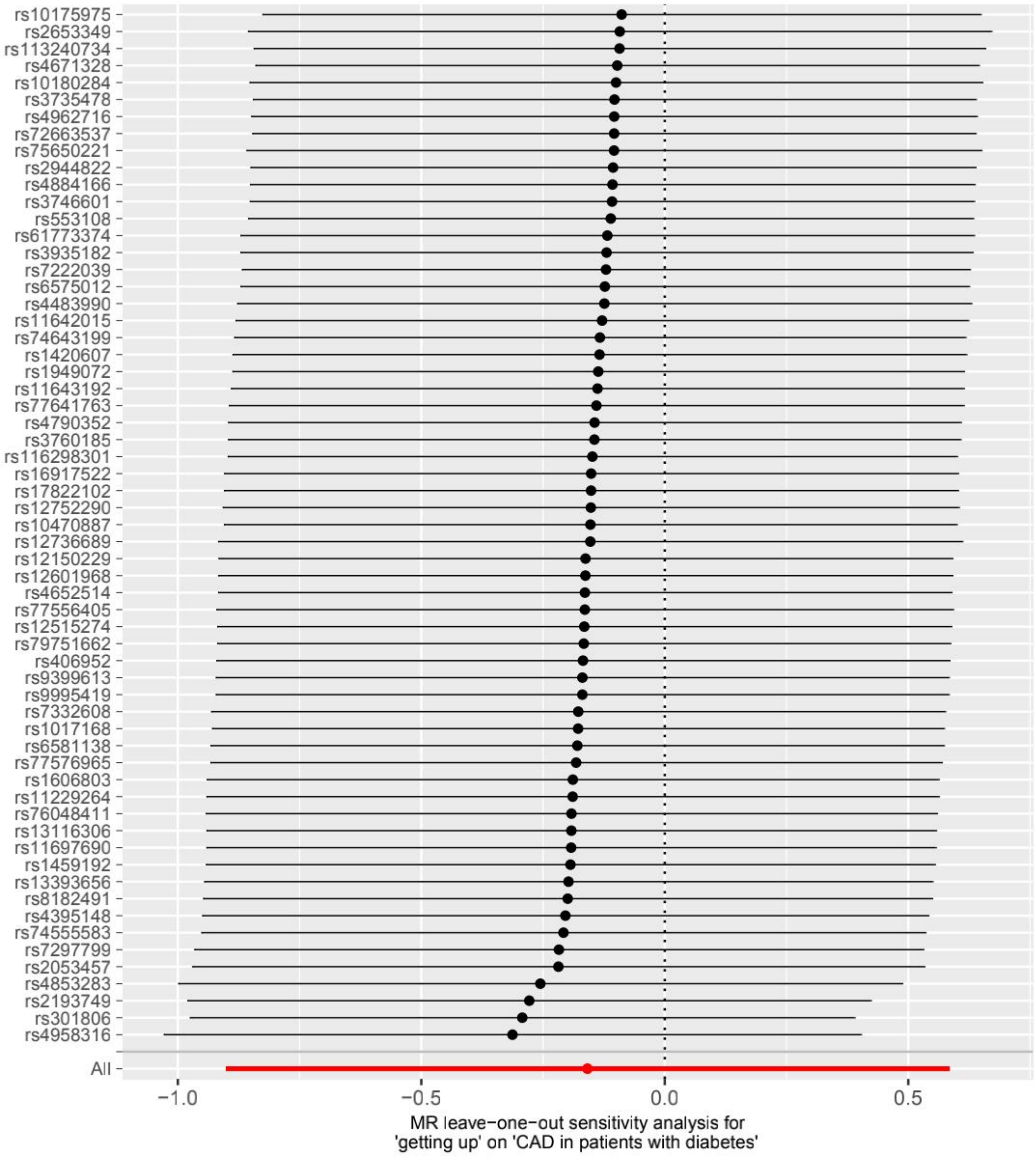

FigureS3.(B)

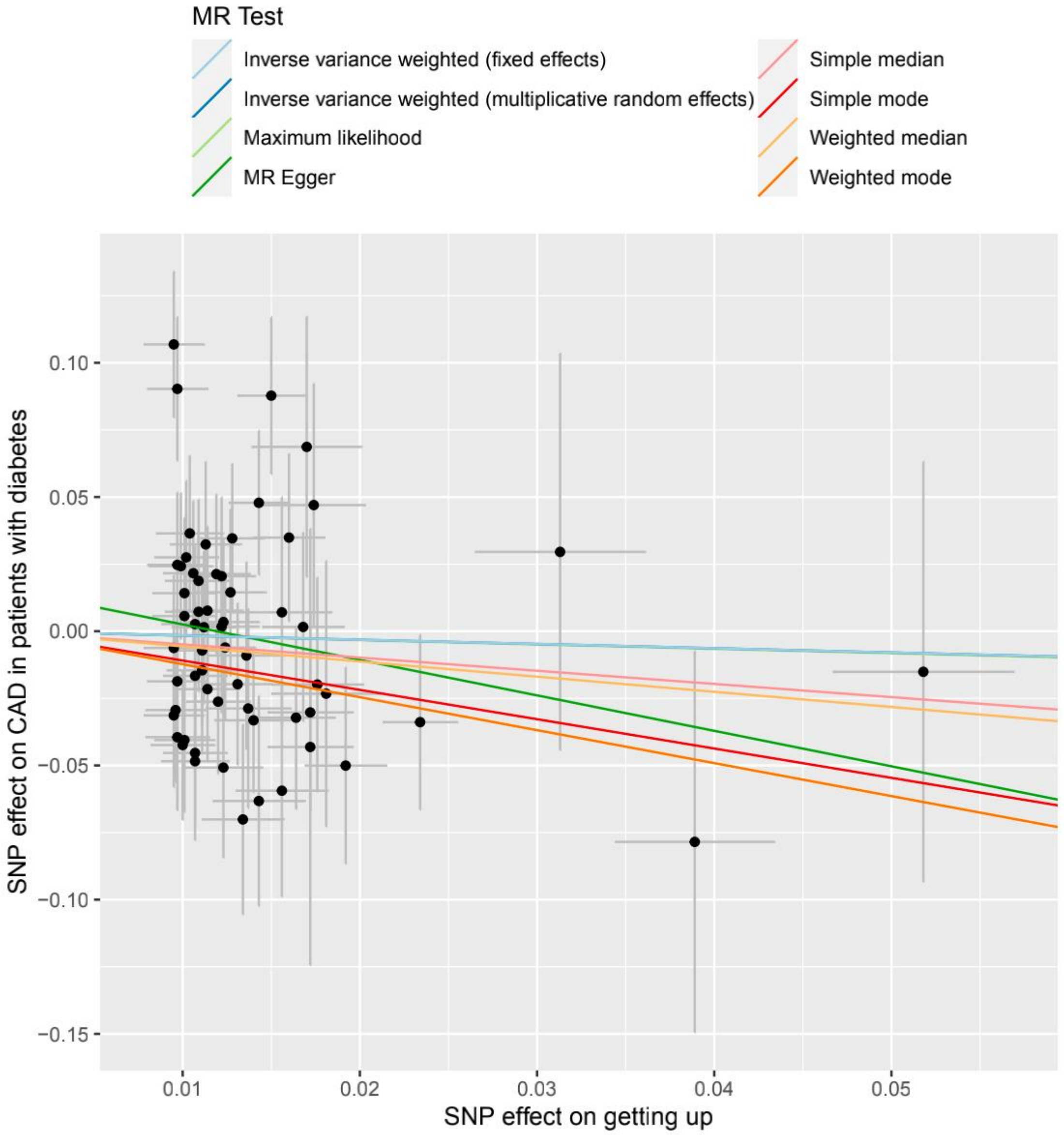

MR Test
Inverse variance weighted (fixed effects)
Inverse variance weighted (multiplicative random effects)
Maximum likelihood
MR Egger
Simple median
Simple mode
Weighted median
Weighted mode
0.10
0.05
0.00
−0.05
−0.10
−0.15
SNP effect on CAD in patients with diabetes
0.01
0.02
0.03
0.04
0.05
SNP effect on getting up

FigureS3.(C)

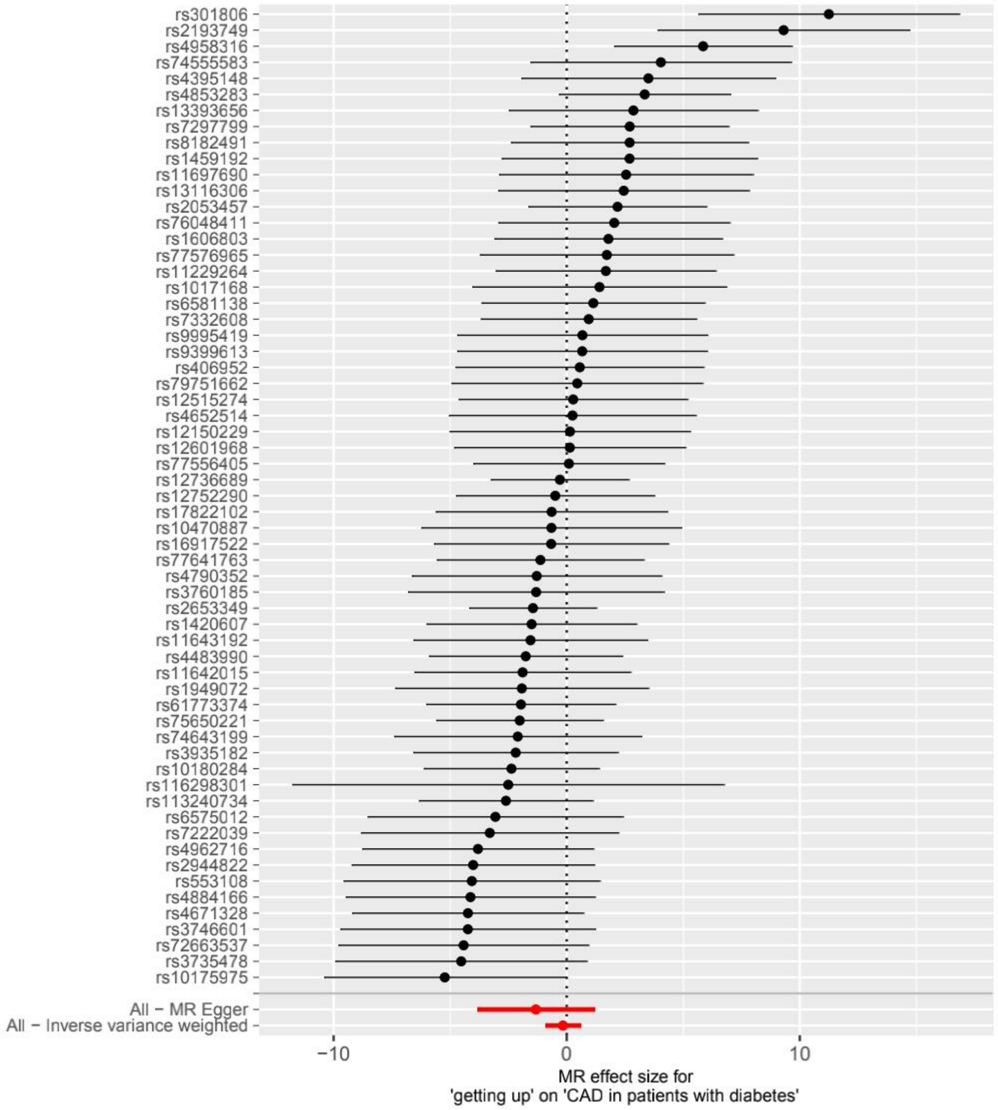
rs301806
rs2193749
rs4958316
rs74555583
rs4395148
rs4853283
rs13393656
rs7297799
rs8182491
rs1459192
rs11697690
rs13116306
rs2053457
rs76048411
rs1606803
rs77576965
rs11229264
rs1017168
rs6581138
rs7332608
rs9995419
rs9399613
rs406952
rs79751662
rs12515274
rs4652514
rs12150229
rs12601968
rs77556405
rs12736689
rs12752290
rs17822102
rs10470887
rs16917522
rs77641763
rs4790352
rs3760185
rs2653349
rs1420607
rs11643192
rs4483990
rs11642015
rs1949072
rs61773374
rs75650221
rs74643199
rs3935182
rs10180284
rs116298301
rs113240734
rs6575012
rs7222039
rs4962716
rs2944822
rs553108
rs4884166
rs4671328
rs3746601
rs72663537
rs3735478
rs10175975
All − MR Egger
All − Inverse variance weighted
−10
0
10
MR effect size for
'getting up' on 'CAD in patients with diabetes'

FigureS3.(D)

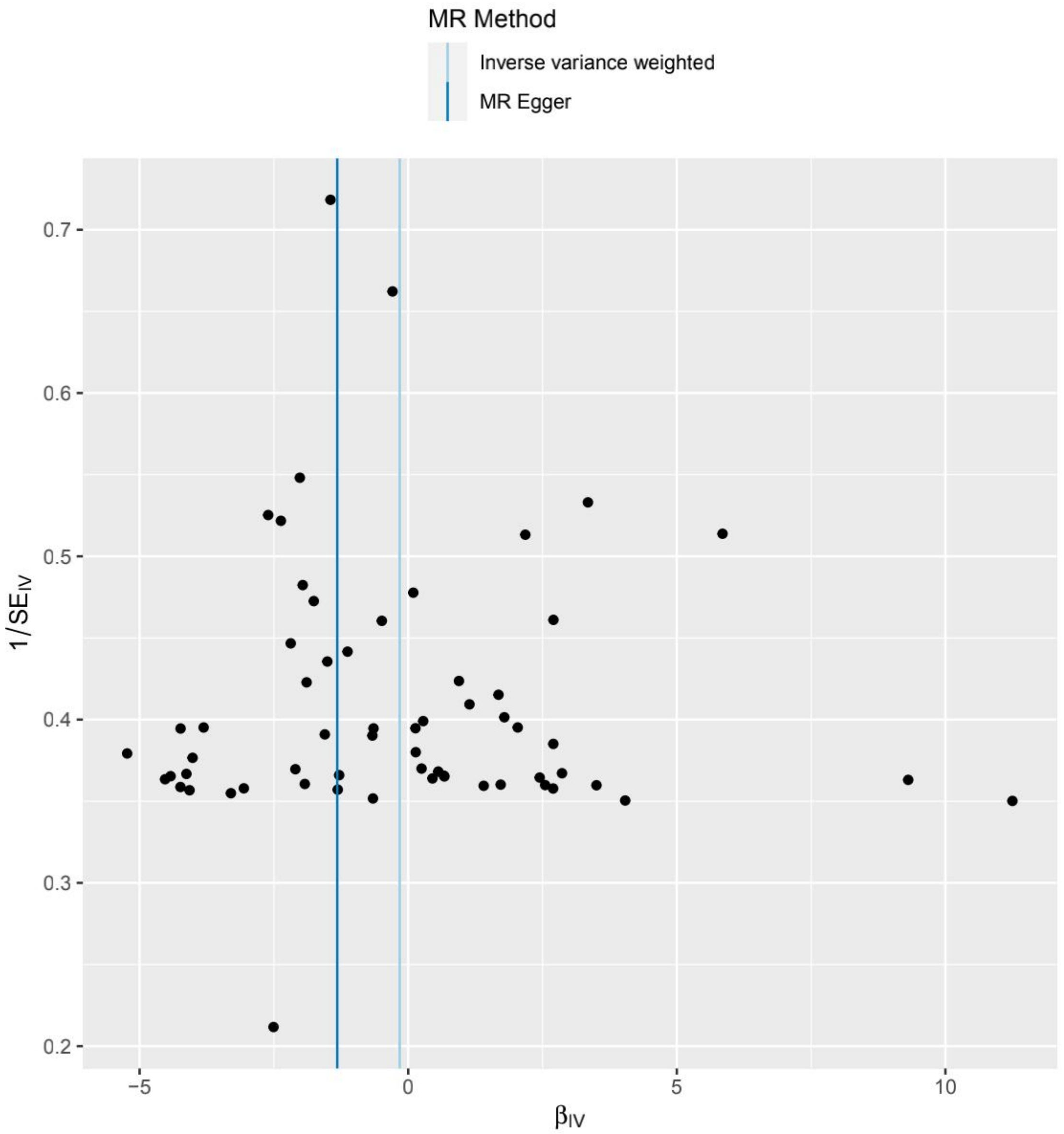
MR Method
Inverse variance weighted
MR Egger
0.7
0.6
0.5
0.4
0.3
0.2
1/SE$_{IV}$
−5
0
5
10
β$_{IV}$

FigureS4. Sensitivity analysis (A), scatter plot (B), forest plot (C), and funnel plot (D) of the causal effect of morningness on CAD in patients with diabetes.

(A)

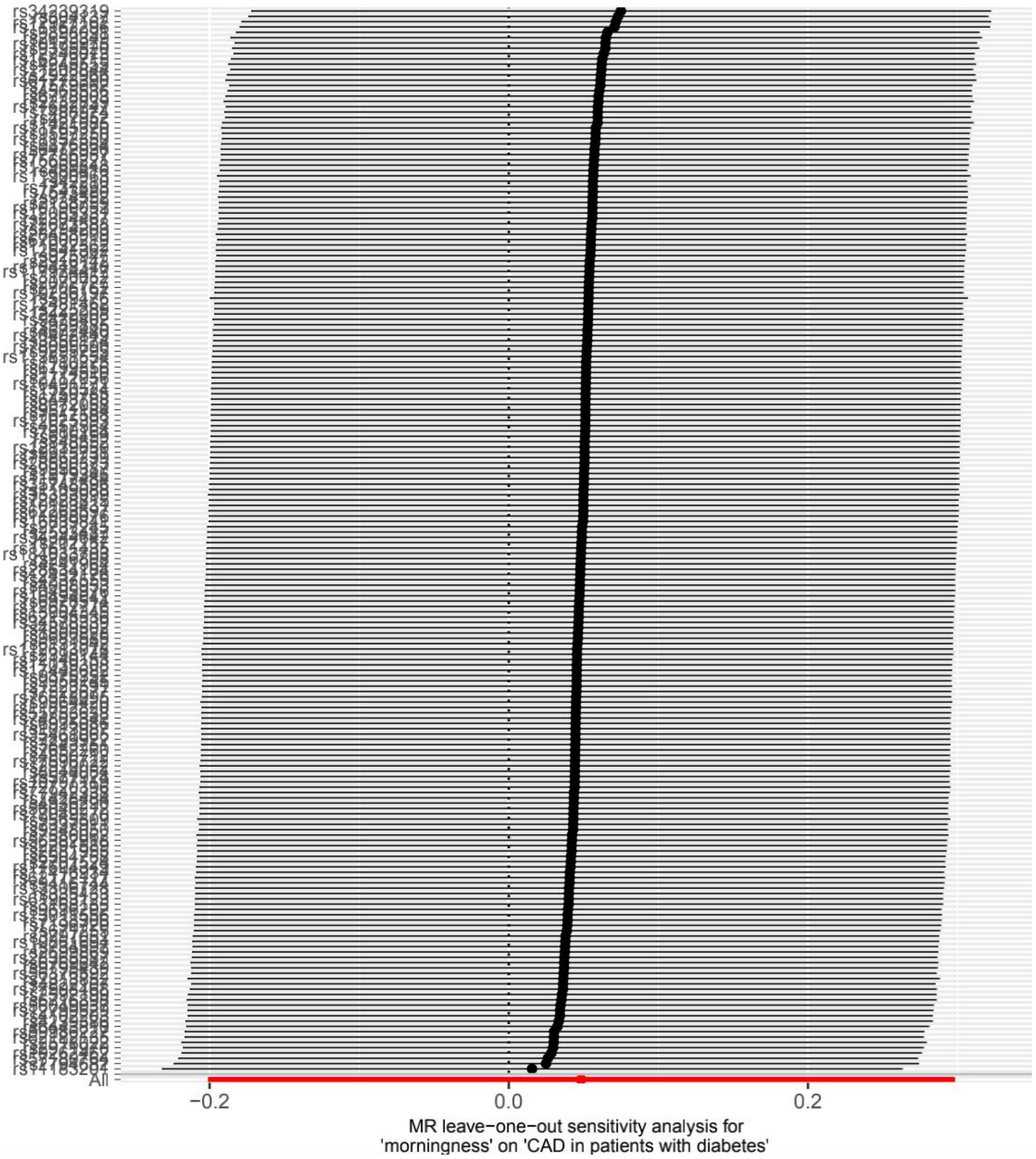

FigureS4.(B)

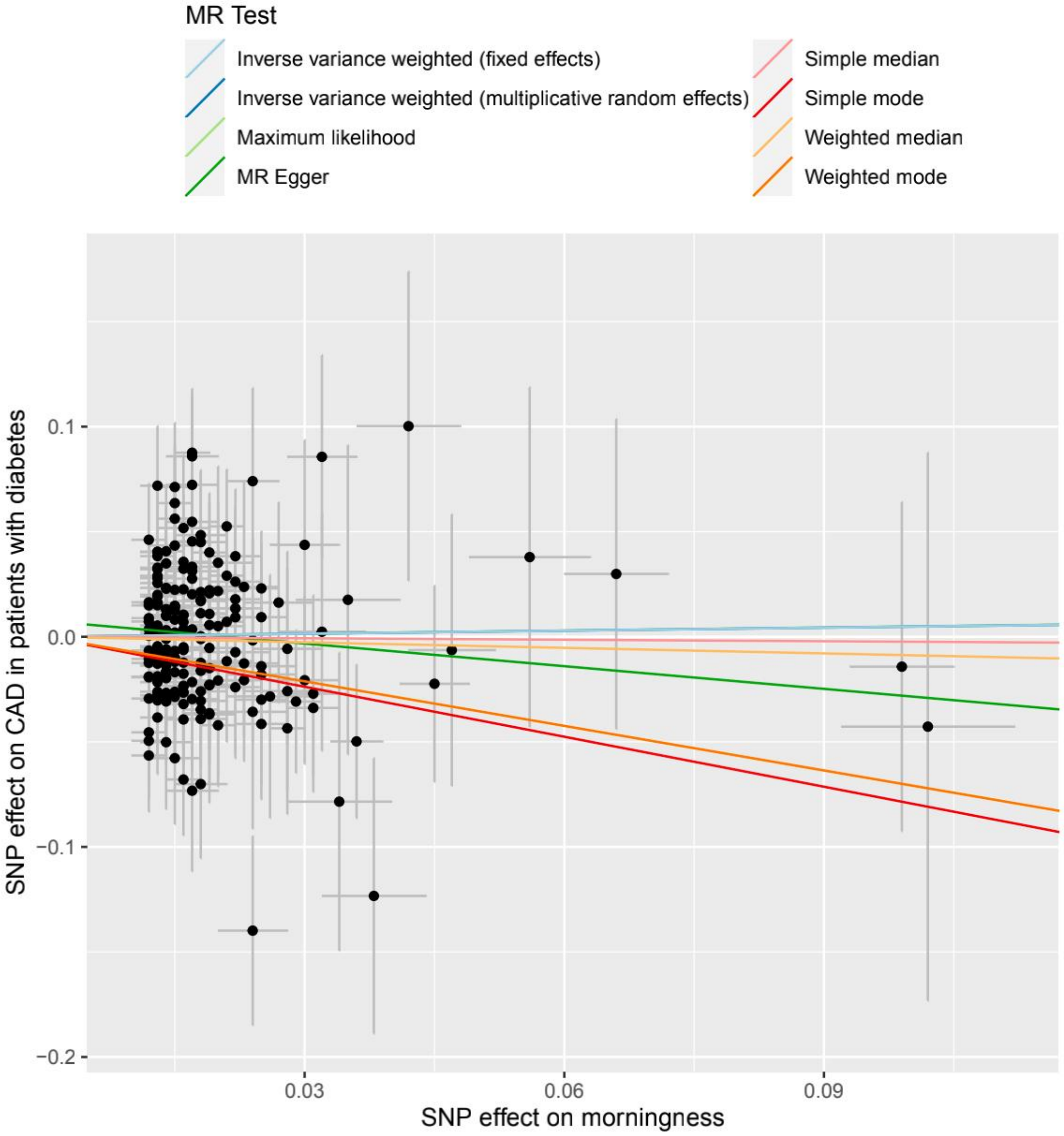

MR Test
Inverse variance weighted (fixed effects)
Inverse variance weighted (multiplicative random effects)
Maximum likelihood
MR Egger
Simple median
Simple mode
Weighted median
Weighted mode
0.1
0.0
−0.1
−0.2
SNP effect on CAD in patients with diabetes
0.03
0.06
0.09
SNP effect on morningness

FigureS4.(C)

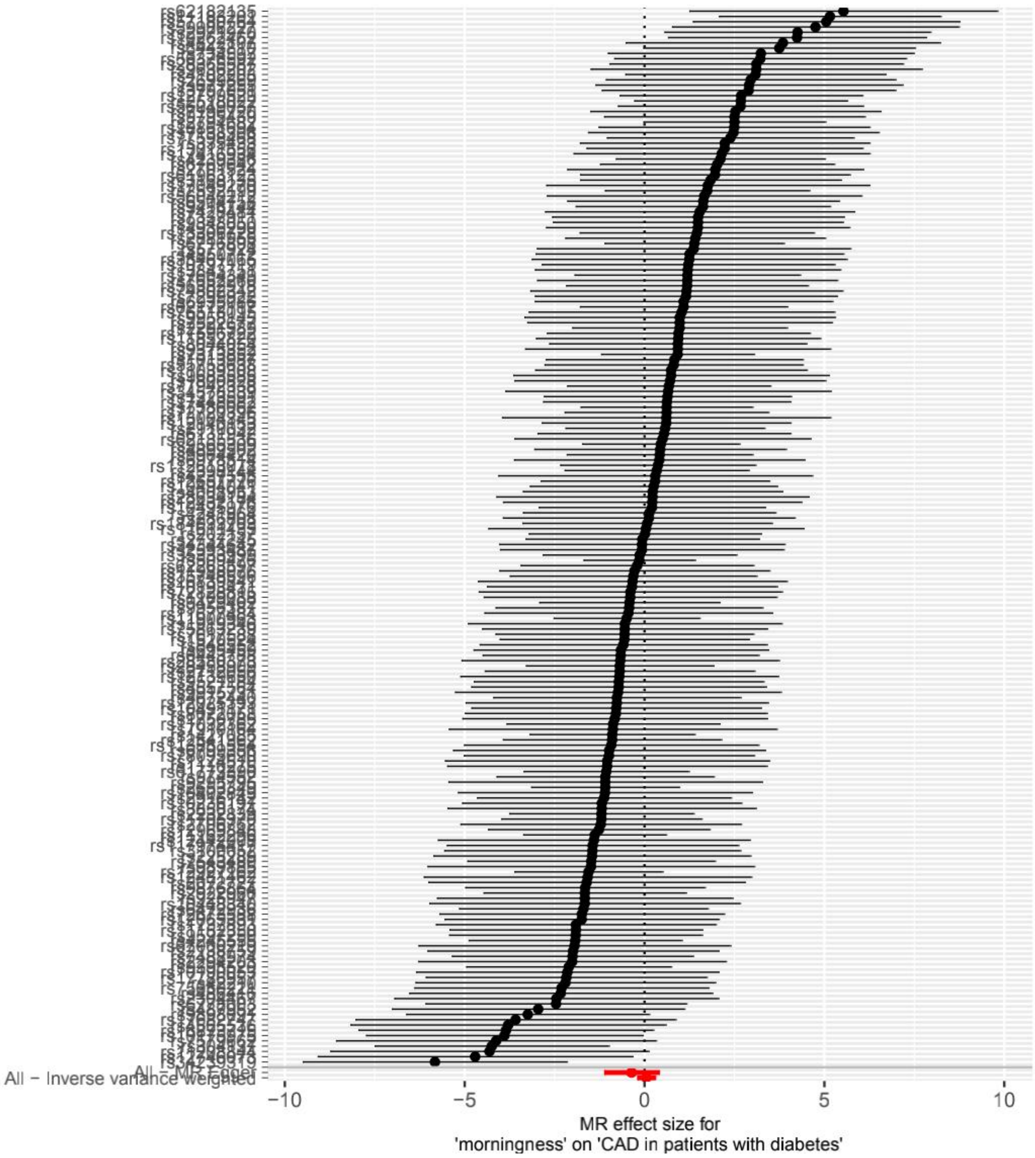

All − MR Egger
All − Inverse variance weighted
−10
−5
0
5
10
MR effect size for
'morningness' on 'CAD in patients with diabetes'

FigureS4.(D)

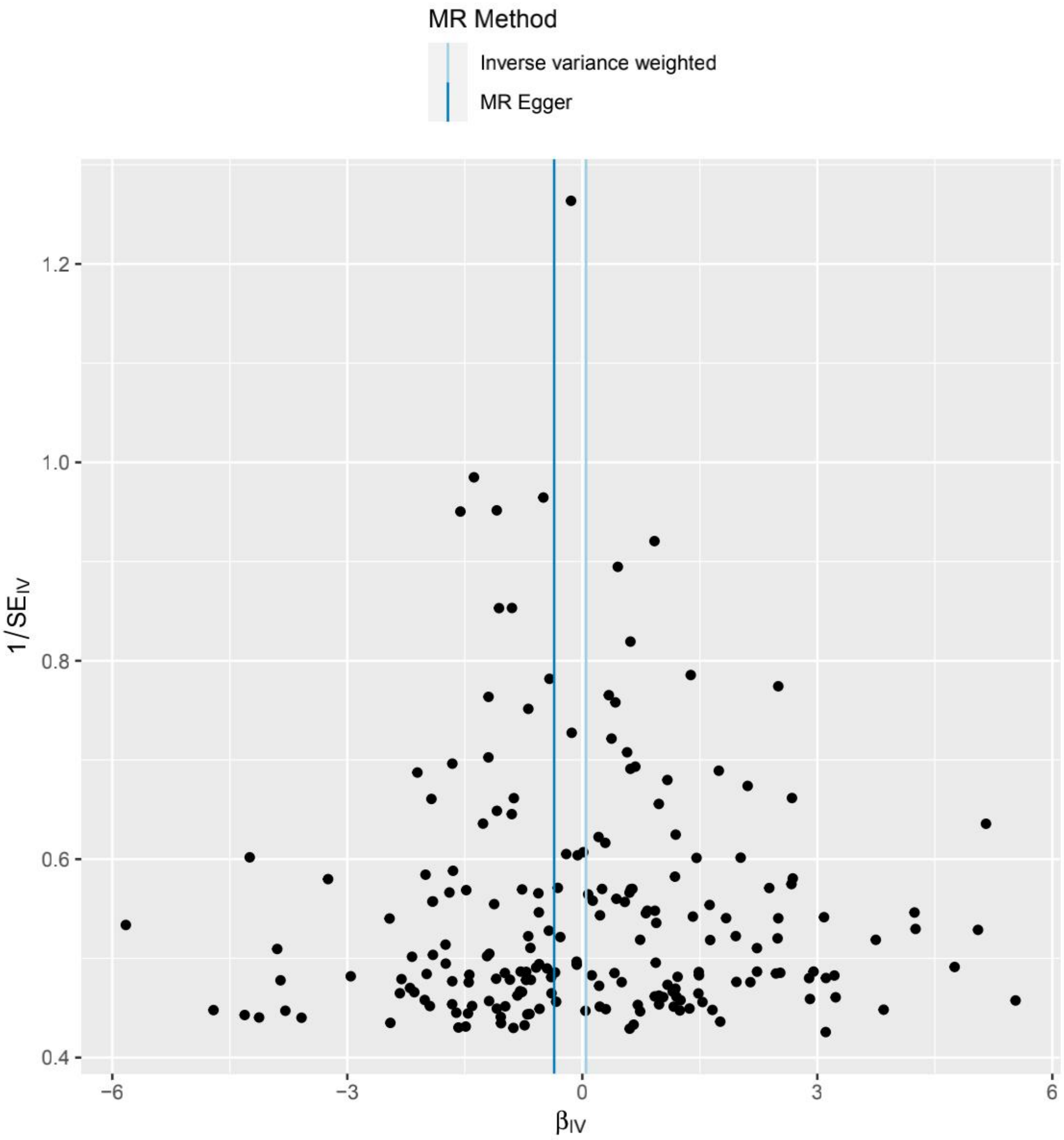
MR Method
Inverse variance weighted
MR Egger
1.2
1.0
0.8
0.6
0.4
1/SE_IV
−6
−3
0
3
6
β_IV

FigureS5. Sensitivity analysis (A), scatter plot (B), forest plot (C), and funnel plot (D) of the causal effect of snoring on CAD in patients with diabetes.

(A)

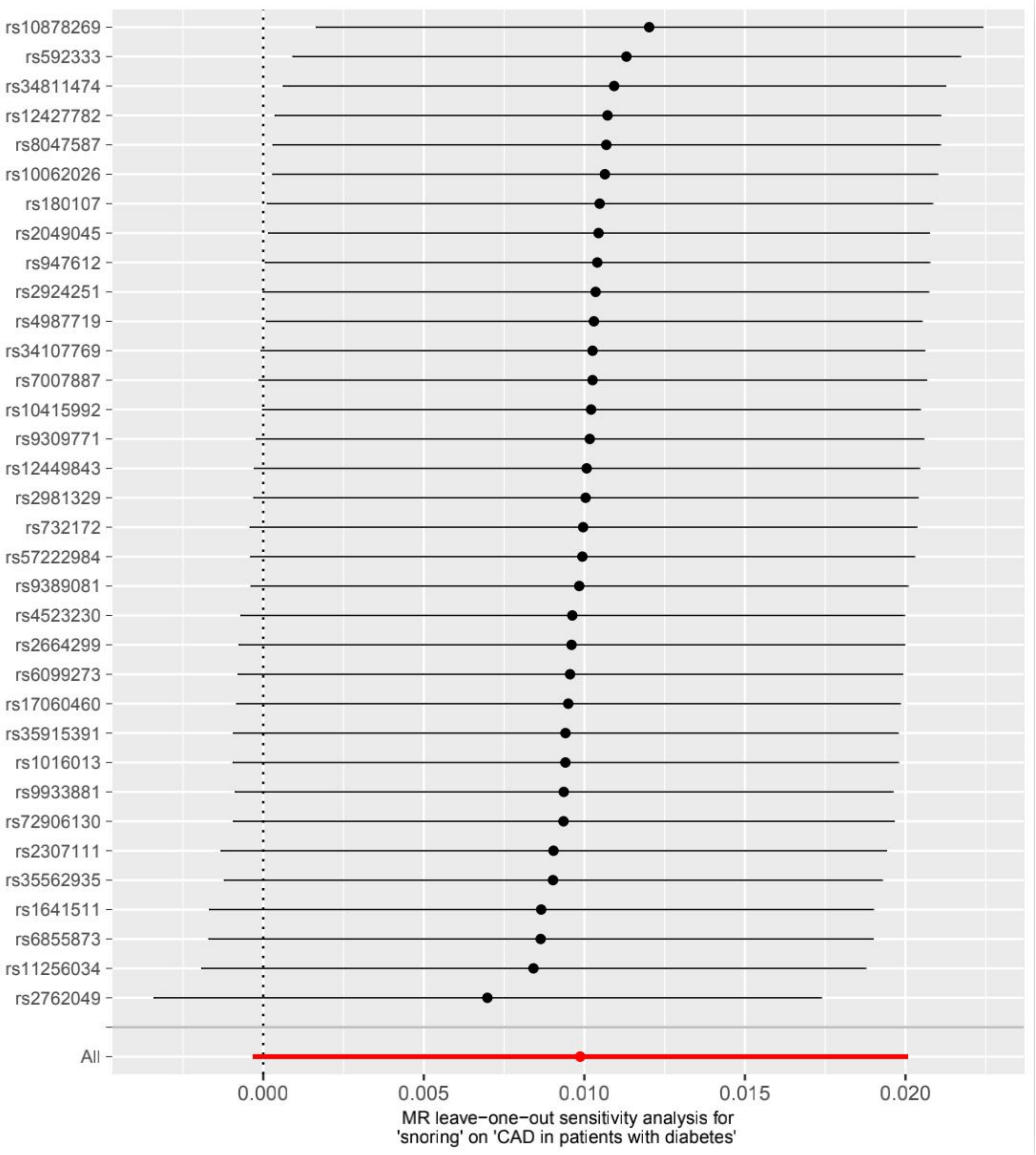

FigureS5.(B)

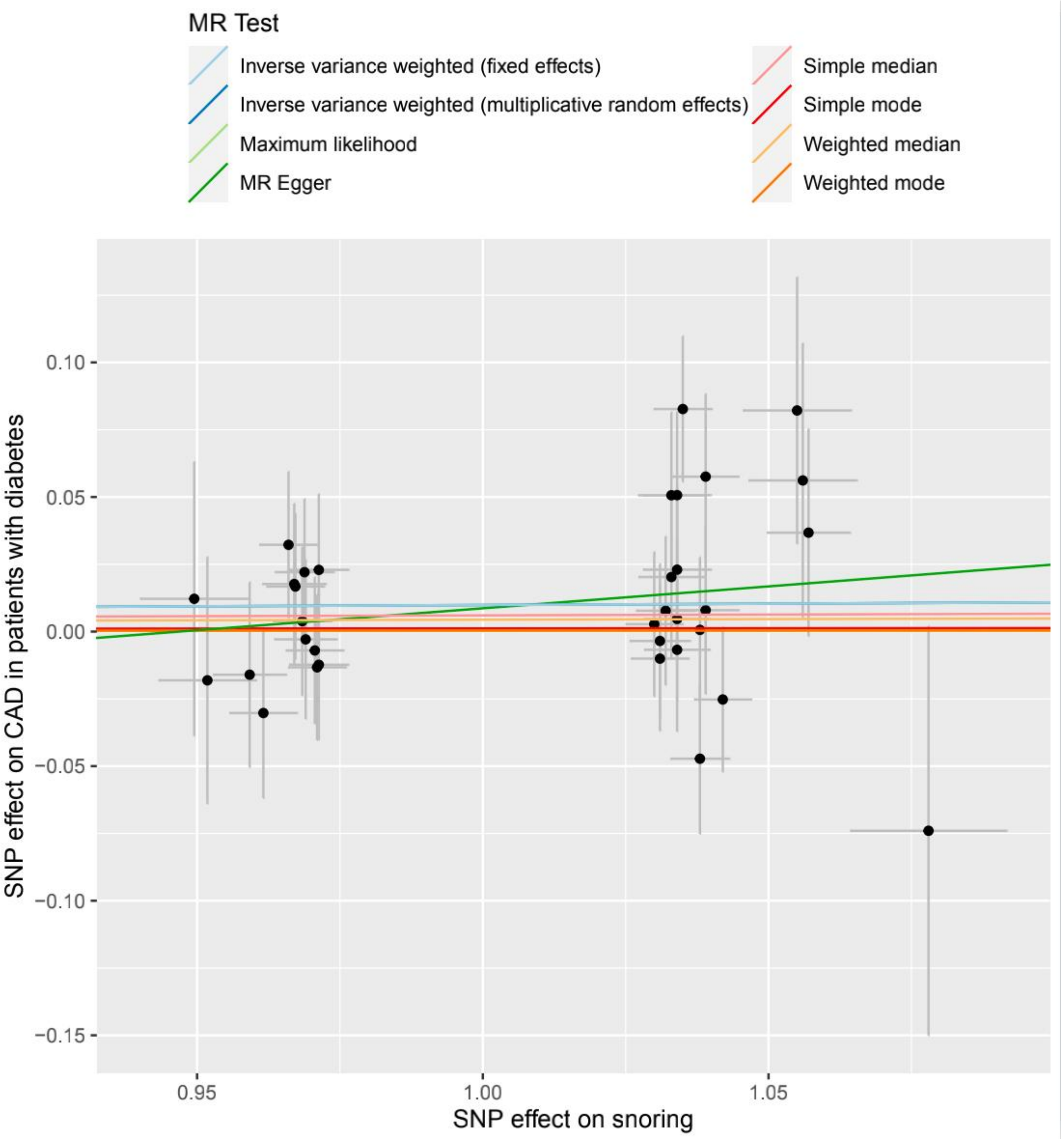

MR Test
Inverse variance weighted (fixed effects)
Inverse variance weighted (multiplicative random effects)
Maximum likelihood
MR Egger
Simple median
Simple mode
Weighted median
Weighted mode
0.10
0.05
0.00
−0.05
−0.10
−0.15
SNP effect on CAD in patients with diabetes
0.95
1.00
1.05
SNP effect on snoring

FigureS5. (C)

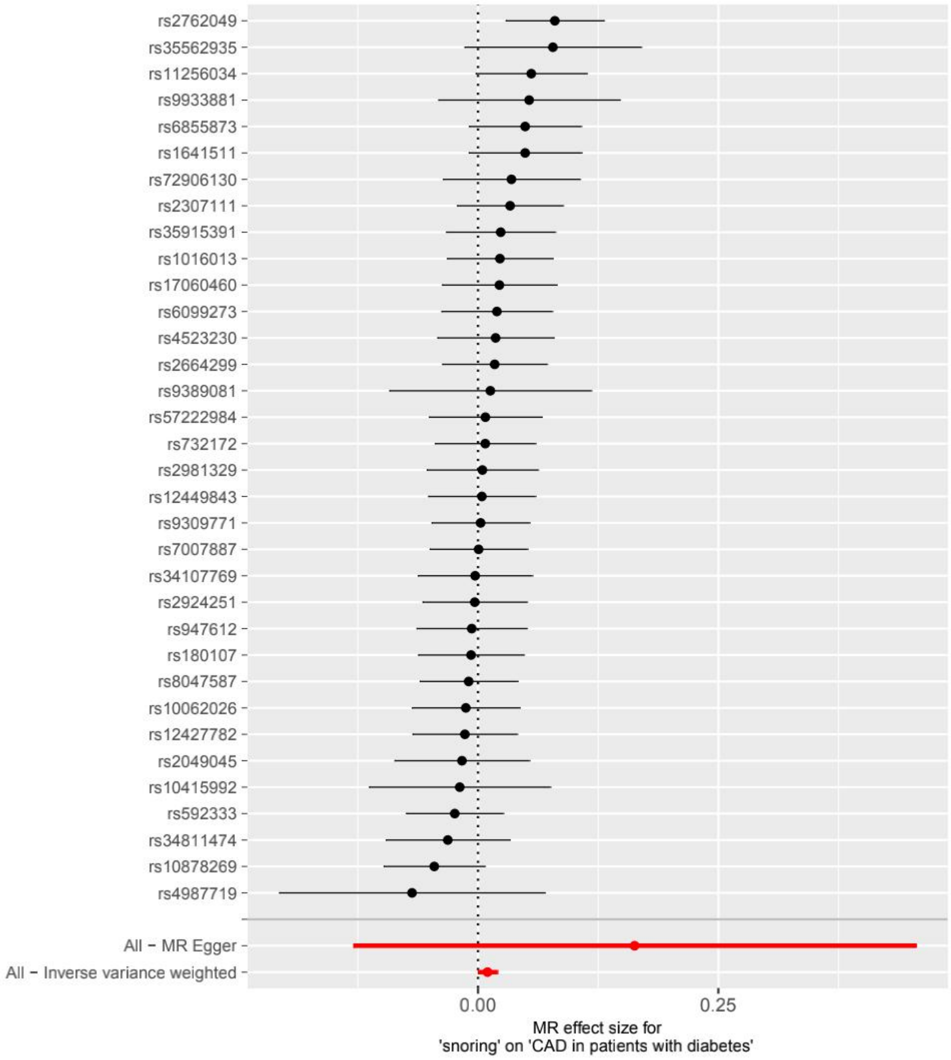
rs2762049
rs35562935
rs11256034
rs9933881
rs6855873
rs1641511
rs72906130
rs2307111
rs35915391
rs1016013
rs17060460
rs6099273
rs4523230
rs2664299
rs9389081
rs57222984
rs732172
rs2981329
rs12449843
rs9309771
rs7007887
rs34107769
rs2924251
rs947612
rs180107
rs8047587
rs10062026
rs12427782
rs2049045
rs10415992
rs592333
rs34811474
rs10878269
rs4987719
All − MR Egger
All − Inverse variance weighted
0.00
0.25
MR effect size for
'snoring' on 'CAD in patients with diabetes'

FigureS5. (D)

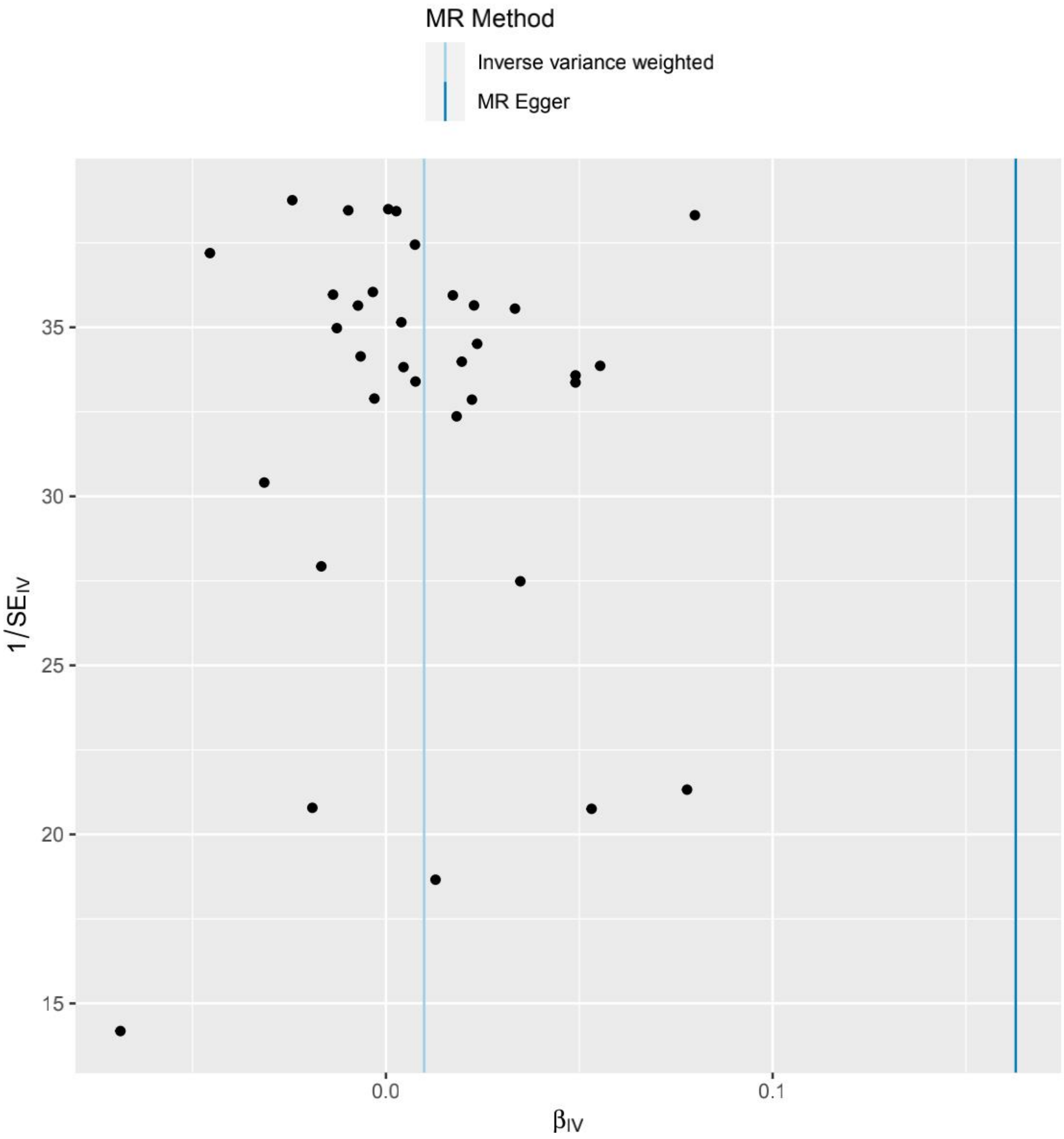

MR Method
Inverse variance weighted
MR Egger
35
30
25
20
15
1/SE$_{IV}$
0.0
0.1
β$_{IV}$
